# Soft ordered double quantitative approximations based three-way decisions and their applications

**DOI:** 10.1038/s41598-022-20982-2

**Published:** 2022-11-10

**Authors:** Gustavo Santos-García, Abbas Ali, Noor Rehman

**Affiliations:** 1 Instituto Multidisciplinar de la Empresa (IME), Universidad de Salamanca, 37007 Salamanca, Spain; 2 Department of Mathematics and Statistics, Riphah International University, Hajj Complex I-14, Islamabad, Pakistan; 3 Department of Mathematics and Statistics, Bacha Khan University, Charsadda, Khyber Pakhtunkhwa Pakistan

**Keywords:** Medical research, Mathematics and computing

## Abstract

The classical theory of rough set was established by Pawlak, which mainly focusses on the approximation of sets characterized by a single equivalence relation over the universe. However, most of the current single granulation structure models cannot meet the user demand or the target of solving problems. Multigranulation rough sets approach can better deal with the problems, where data might be spread over various locations. In this article, we present the idea of soft preference and soft dominance relation for the development of soft dominance rough set in an incomplete information system. Subsequently, several important structural properties and results of the proposed model are carefully analyzed. After employing soft dominance based rough set approach to it for any times, we can only get six different sets at most in an incomplete information system. That is to say, every rough set in a universe can be approximated by only six sets, where the lower and upper approximations of each set in the six sets are still lying among these six sets. The relationships among these six sets are established. Based on soft dominance relation, we introduce logical disjunction/conjunction soft dominance optimistic/pessimistic multigranulation decision theoretic rough approximations in an incomplete information. Meanwhile, to measure the uncertainty of soft dominance optimistic/pessimistic multigranulation decision theoretic rough approximation and some of their interesting properties are examined. Thereafter, a novel multi attribute with multi decision making problem approach based on logical disjunction/conjunction soft dominance optimistic/pessimistic multigranulation decision theoretic rough sets approach are developed to solve the selection of medicine to treat the coronavirus disease (COVID-19). The basic principle and the detailed steps of the decision making model (algorithms) are presented in detail. To demonstrate the applicability and potentiality of the proposed model, we present a practical example of a medical diagnosis is given to validate the practicality of the technique.

## Introduction

There are various problems in our daily life that include uncertainty in the data that is related to them. Such problems are prominent in economics, engineering, environmental sciences, medical sciences, and a variety of other disciplines that are dependent on modeling uncertainties that cannot be tackled by traditional mathematical techniques. In recent times, several researchers have endeavored to establish some suitable techniques, and mathematical theories to cope with these uncertainties, such as fuzzy set theory, rough set theory, decision making theory, etc., have been formulated to tackle these problems but have only been found partly effective^1–6^. These theories bridged the gap amongst conventional mathematical models and ambiguous real-world data.

### A brief review on rough set theory/soft set theory and their applications

The main concept of rough set theory was initiated by Pawlak^7^, at the beginning of the 9th decade in the 20th century. This theory depends on the lower and upper approximations to analyze the data. It works by classifying the data into three regions interior, exterior and boundary regions and which works on maximizing the interior region and minimizing the exterior region that concludes to minimize the boundary region. The first definition of rough set theory is built on an equivalence relation between variables. Based on binary relations, Abu-Donia introduced different kinds of approximations and their mutual relationships^8^. Yao^9^, investigated the relational interpretations of neighborhood operators and rough set approximation operators. Zhu^10^, applied binary relation to introduce generalized rough sets and their applications. Skowron and Stepaniuk^11^, discussed the concept of rough approximation based on tolerance relation and their applications. Abo Tabl^12^, used similarity relation for the comparison of two kinds of definitions of rough approximations and their mutual relationships. Slowinski and Vanderpooten^13^, introduced the concept of generalized rough approximations using similarity relation and their applications in data engineering. Dai et al.^14^, initiated the idea of multiple neighborhoods from a similarity relation and presented the idea of generalized rough set models. Based on fuzzy algorithms, Huang et al. determined the threshold value $\beta$ of variable precision rough sets^15^. Yao^16^, introduced the concept three-way decisions based on probabilistic rough sets. Ziarko^17^, presented the notion of variable precision rough set model which is more generalized idea than the Pawlak’s model. Bartol et al. initiated the concept of covering rough sets by replacing the equivalence classes by tolerance classes^18^. Bianucci et al.^19^, investigated some important properties of covering based rough sets and extended these to incomplete information systems. Based on boolean matrices, Ma gave the idea of covering rough sets and discussed some of their interesting properties^20^. Zhu applied topological approach for the notion of generalized rough set called covering rough sets and investigated some important properties related to covering rough sets^21^. Zhu and Wang developed a technique for the construction of reduction of attributes to apply covering rough sets and elaborated their applications^22^. As a consequence, Zhu and Wang proposed the idea of different classifications of generalized rough sets and discussed some of their properties^23^. Subsequently, Zhao investigate various classifications of covering rough sets by using the topological methodology and elaborated their applications^24^.

Dubois and Prade presented the idea of hybridized structures called rough fuzzy sets and fuzzy rough sets by combining of rough sets and fuzzy sets^25^. Also in the context of decision making, Slowinski and Stefanowski applied rough fuzzy sets for finding the best solutions of medical related problems^26^. Li et al.^27^, presented the notion of generalized fuzzy rough sets on the basis of fuzzy coverings and investigated their various properties for the developing of the study. D’eer et al. put forward a new technique for the development of fuzzy covering approximation theory based on fuzzy neighborhood operators and discussed their properties^28^. Ma^29^, introduced the notion of $\beta$-covering which is the generalization of fuzzy covering and studied the vital properties of $\beta$-covering such as fuzzy $\beta$-neighborhoods and fuzzy $\beta$-covering rough sets. Subsequently, Yang and Hu used the idea of $\beta$-covering to presented different types of fuzzy covering rough sets and their applications related to daily life problems^30^. Greco et al.^31–34^, presented the new idea of rough set by applying dominance relation to handle multi criteria group decision making problems. Blszczynski et al.^35^, presented the notion of new rough set model by applying dominance relation and discussed the monotonicity of a consistent variable. Huang^36^, introduced a new type of interval valued rough set technique by applying graded dominance relation. Further, Huang et al. initiated the idea of rough set model in intuitionistic fuzzy information environment by applying dominance relation^37^. Hu and Chen^38^, presented the concept of variable precision rough set model by using dominance relation and further they investigated their various properties for the development of this study. Inuiguchi et al.^39^, applied dominance relation for the construction of generalized rough set model and initiated a new technique for the reduction of attributes. Kotlwski et al.^40^, presented a new concept of rough set model based on stochastic dominance relation for the construction of ordinal classification. Yang et al.^41^, initiated the concept of $\alpha$-dominance relation and introduced a new rough set model in interval-valued fuzzy environment. Li and Li^42^, presented the incremental approximations by applying dominance relation and investigated their various properties for the development of this study. Based on dominance relation, Lingras et al.^43^, introduced a new idea of combining rough clustering technique with qualitative and quantitative crisp sets and discussed some of their applications.

Molodtsov^44^ initiated the concept of the soft set in 1999,  which can be viewed as an entirely new mathematical technique for modeling uncertainty; where a soft set is connected with an adequate set of attributes and therefore free from the difficulties. Unlike classical mathematics, which demands an exact solution to a mathematical model, soft set theory embraces an approximate description of an item as its initial point. The use of appropriate parameterization tools, like functions, linguistic phrases, numbers, etc., makes soft set theory highly practical and simple to implement in reality. Feng et al.^45^, introduced a new concept of hybrid structures called rough soft sets and soft rough fuzzy sets and discussed some of their basic properties. Further, Feng et al.^46^, combined the structures of soft sets and rough sets to introduce a new concept of hybrid structures called soft rough sets and discussed some of their basic properties. Meng et al.^47^, combined the notions of soft sets, fuzzy sets and rough sets to introduce a new concept of hybrid structures called soft rough fuzzy sets and soft fuzzy rough sets and investigated their various properties. Sun and Ma^48^, initiated the idea of new hybrid structure called soft fuzzy rough sets and discussed its application in fuzzy information environment. Zhan and Zhu^49^, applied the structure of Z-soft rough fuzzy sets on algebraic structure to present a decision making methodology.

### A short introduction to the multigranulation approximations

In practical life, we often need to describe the concept via multiple relations over the universe based on user requirement or the target of tackling the problem. Hence, Qian et al. in^50^ extended the single granulation rough set model to the multigranulation rough set model, which emerged as a prominent topic in artificial intelligence, attracting a wide range of researchers to both theoretical and application perspectives. Based on fuzzy information environment, Feng and Mi introduced the idea of generalized multigranulation fuzzy decision theoretic rough set model and presented its application in three way decision analysis^51^. Li et al.^52^, presented the notion of three types of double quantitative multigranulation decision theoretic rough fuzzy set models and discussed case study related to medical diagnosis problem. Also in the context of decision making, Lin et al. developed the idea of multigranulation rough sets by using neighborhood approach^53^. She and He^54^, discussed the topological structure and the lattice structure on the basis of three different kinds of definable sets obtained from multigranulation rough set model. Sun et al.^55^, established multigranulation approximation technique for two universes and applied it to group decision making. Xu et al.^56^, initiated the notions of support characteristic function and information level for the construction of generalized multigranulation approximation theory and discussed their application in granular selection. Xu et al.^57^, applied tolerance relations for the construction of multigranulation approximation theory. After that, Xu et al. described the idea of multigranulation approximations in fuzzy environment and investigated their various properties for the developing of this study^58^. Based on tolerance relations, Xu et al. established the notion of generalized fuzzy rough approximations in fuzzy environment with applications to decision making methodology^59^. Yang et al.^60^, established the relationship between three different hierarchical structures and different multiple granulation approximations and discussed some of their properties. You et al.^61^, presented the idea of multiple granulation approximations in the environment of neighborhood covering information by applying the notion of neighborhood of objects.

### The scientific gap and motivation of our research

Rough set model itself is relatively simple, and there are some shortcomings in the actual application process. For example, the data sets in practical application are often disturbed by noise and the data are not necessarily complete, which will affect the equivalent classification of data sets. The lower approximation set is based on strict inclusion and can not deal with some extent of inclusion and belonging. Therefore, variable precision rough set^17^ and graded rough set^62^ are proposed. Variable precision rough set model is mainly used to describe the quantitative index with precision. Precision can reflect the relative quantization information of the intersection relationship between knowledge equivalence class and concept set. The graded rough set model primarily considered the absolute quantitative information regarding the basic concepts and knowledge granules and is a generalization of the Pawlak model. Graded rough set model mainly considered absolute quantitative information between equivalence classes and the basic concept.

Variable precision rough set and graded rough set, as two useful expanded rough set models, can respectively reflect relative and absolute quantitative information about the degree of overlapping between equivalence classes and a basic set. The relative and absolute quantitative information are two distinctive objective sides that describe approximate space, and each has its own virtues and application environments, so none can be neglected. Though relative quantitative information and absolute quantitative information are two kinds of quantification methodologies in certain applications, people also tend to prefer the description of relative quantitative information. However, there are always two different sides in the same coin. Relative quantification and absolute quantification are two different aspects of objective things and the relationship between them is dialectical and interdependent. Therefore, people should also attach importance to absolute quantification. In fact, absolute quantitative information may play a leading role in some special fields and environments.

Precision and grade are two important quantitative indexes of data, and related to the relative and absolute quantitative information respectively. In real life, there are some issues that not only the relative quantitative information but also the absolute quantitative information should be considered. Meanwhile we need to describe concurrently a target concept through multi binary relations according to users requirements and targets of problem solving. Based on these considerations, in this paper we focus on combining variable precision rough set model with graded rough set model by using logical operators in multigranulation approximate space. We construct the multigranulation rough set model which considers the relative and absolute quantitative information about the degree of overlapping between soft dominance classes and a concept set.

In this article, we present the idea of soft preference and soft dominance relation for the development of soft dominance rough set in an incomplete information system. Subsequently, several important structural properties and results of the proposed model are analyzed. After employing soft dominance based rough set approach to it for any times, we can only get six different sets at most in an incomplete information system. That is to say, every rough set in a universe can be approximated by only six sets, where the lower and upper approximations of each set in the six sets are still lying among these six sets. The relationships among these six sets are established. Based on the practical logical requirements of precision and grade, we introduce four pairs of new logical disjunction/conjunction soft dominance optimistic/pessimistic multigranulation decision theoretic rough approximations in an incomplete information. Meanwhile, to measure the uncertainty of soft dominance optimistic/pessimistic multigranulation decision theoretic rough approximation and some of their interesting properties are examined. Thereafter, a novel multi attribute with multi decision making problem approach based on logical disjunction/conjunction soft dominance optimistic/pessimistic multigranulation decision theoretic rough sets approaches are developed to solve the selection of medicine to treat the coronavirus disease (COVID-19). The basic principle and the detailed steps of the decision making model (algorithms) are presented in detail. To demonstrate the applicability and potentiality of the developed model, we give a practical example of medical diagnosis is given to validate the practicality.

The arrangement of this article is as follows: “Problem statement” section focuses mainly on the problem statement. In “Proposed conflict analysis model” section we present the idea of soft preference and soft dominance relation in an incomplete information system to solve a multi agent conflict analysis for coronavirus disease (COVID-19). In “Logical multigranulation rough sets using soft dominance approach” section we focus our attention on the development of feasible consensus strategy for coronavirus disease (COVID-19) based on soft dominance based logical optimistic/pessimistic multigranulation rough sets with three way decision analysis. The idea of two new kinds of approximate precision, rough degree, approximate quality with the help of soft dominance based logical optimistic/pessimistic multigranulation rough sets is also given. Finally, the key findings of our study are summarized in “Conclusion” section.

## Problem statement

Currently, the emergence of a novel human coronavirus, (SARS-CoV-2) or (COVID-19), has become a global health concern which causes severe respiratory tract infections in humans. Human to human transmissions have been described with incubation times between 2-10 days, facilitating its spread via droplets, contaminated hands or surfaces. Human corona viruses can remain on inanimate surfaces for up to 9 days. A novel corona virus, (SARS-CoV-2) or (COVID-19), has recently emerged from China with a total of 45171 confirmed cases of pneumonia (as of February 12, 2020). The two impact factors for infections transmission namely “Contact with infected surfaces” and “Interactions with infected people” of the virus. It is concluded that the corona virus is a burning issue and needs mathematical formulation/technique for selection of medicine for treatment of the disease. In this paper, we introduce an application to show how our approaches are the best tool in decision making about the selection of medicine for COVID-19 using multigranulation rough sets.

The incomplete multi decision problems with preference relations have been studied in this paper. An incomplete decision problem may be considered as an ${\mathcal {I}}{\mathcal {S}}^{*}=\left( A,\ C,\ D,\ E\right)$, where *A* is a finite set of medicines $b_{i},i=1,2,3,...,\left| A\right| ,$ *C* is a finite set of symptoms of corona virus disease (COVID-19) (conditional attributes) $C_{j},j=1,2,...,\left| C\right| ,$ *D* is a finite set of doctors (decision attributes) $D_{k},k=1,2,...,\left| D\right|$ and *E* is a finite set of the domain for the information functions $f\left( b_{i},C_{j}\right)$ and $g\left( b_{i},D_{k}\right) .$ It may happen that some symptoms (attribute) values of an medicine are missing. To indicate such a situation, a distinguished value is often assigned. We use special symbol “$*$” to indicate the missing attribute value. For an information system, if there exist $b_{i}\in A,$ $C_{j}\in C$ and $D_{k}\in D$ such that $f\left( b_{i},C_{j}\right) =*$ or $g\left( b_{i},D_{k}\right) =*.$

In order to show the incomplete decision problem clearly, an example of a conflict situation for corona virus disease (COVID-19) is presented in Table 1. There are five symptoms $$\left( \text {conditional attributes}\right) $$ and four doctors $$\left( \text {decision attributes}\right) $$ with twelve medicines $$A=\left\{ b_{i}:i=1,2,...,12\right\} $$. The symptoms may be $$ C_{1}=$$ Inability to arouse (wake up from sleep), $$C_{2}=$$ difficulty breathing or shortness of breath, $$C_{3}=$$ chest pain or pressure, $$C_{4}=$$ loss of speech or movement and $$C_{5}=$$ loss of taste or smell, while $$ D_{1}, $$ $$D_{2},$$ $$D_{3}$$ and $$D_{4}$$ are doctors to handle the conflict situation for corona virus disease (COVID-19). The association of the integers are defined as follows: 0-bad, 1-average,  2-good,  3 -excellent.

**Table 1:** Incomplete multi criteria and multi decision information table.

*A*	$C_{1}$	$C_{2}$	$C_{3}$	$C_{4}$	$C_{5}$	$D_{1}$	$D_{2}$	$D_{3}$	$D_{4}$
$b_{1}$	1	0	1	$*$	2	2	$*$	1	$*$
$b_{2}$	1	$*$	1	1	2	1	3	0	1
$b_{3}$	1	0	2	0	2	3	1	0	3
$b_{4}$	1	1	0	0	2	1	1	$*$	2
$b_{5}$	$*$	1	1	0	2	0	2	0	1
$b_{6}$	1	1	2	1	2	1	0	2	0
$b_{7}$	1	1	2	2	2	3	2	3	2
$b_{8}$	1	2	$*$	0	2	0	$*$	0	2
$b_{9}$	1	2	1	1	2	2	2	1	$*$
$b_{10}$	1	2	0	0	$*$	$*$	3	0	3
$b_{11}$	1	2	1	0	1	0	1	2	0
$b_{12}$	1	2	1	1	1	2	1	1	1

## Proposed conflict analysis model

Analysis of conflict described in^63^ is restricted to outermost conclusions, such as finding the most conflicting attributes or the coalitions of agents if more than two take part in the conflict. Because in the Pawlak’s model the reason of the conflict cannot be determined, there is no way to specify the situation to avoid the conflict. Moreover, we cannot be sure that the issues the agents vote represent the issues each agent takes care of. Though the Pawlak’s conflict analysis model has proven to be an effective method in practice, yet Deja in^64^, put forward three basic (below given) questions which are not answered by the Pawlak’s conflict analysis model:

(i) *What are the intrinsic reasons for the conflict*?
(ii) *How can a feasible consensus strategy be found*?
(iii) *Is it possible to satisfy all the agents*?

Let ${\mathcal {P}}_{C_{j}}$ be an outranking relation on a universe *A* with reference to criteria $C_{j}\in C$ such that $b_{i}C_{j}b_{j}$ which means “$b_{i}$ is at least as good as $b_{j}$ with respect to criteria $C_{j}.$” Suppose that ${\mathcal {P}}_{C_{j}}$ is a complete preorder, We employed dominance relation for the study of the problem as follows: denote $b_{i} \succeq b_{j}$ by $f\left( b_{i},C_{j}\right) \ge f\left( b_{j},C_{j}\right)$ according to increasing preference, where $C_{j}\in C$ and $b_{i},b_{j}\in A.$ For any subset of the conditional attributes ${\mathcal {C}}\subseteq C$, $b_{i} \succeq _{ {\mathcal {C}}}b_{j}$ means that $b_{i} \succeq _{C_{j}}b_{j}$ for all $C_{j}\in {\mathcal {C}};$ that is, $b_{i}$ dominates $b_{j}$ with respect to all attributes in ${\mathcal {C}}.$ The intersection of complete preorders is a partial preorder and ${\mathcal {P}}_{{\mathcal {C}}}=\bigcap \nolimits _{C_{j}\in {\mathcal {C}}}{\mathcal {P}}_{C_{j}},$ the dominance relation ${\mathcal {P}}_{ {\mathcal {C}}}$ is a partial preorder.

### Definition 1

Let ${\mathcal {I}}{\mathcal {S}}^{*}=\left( A,C,D,E\right)$ be an incomplete information system and ${\mathcal {T}}_{k}^{*}:C\rightarrow {\mathcal {P}}\left( A\times A\right)$ be a set valued mapping from *C* to ${\mathcal {P}}\left( A\times A\right) .$ If ${\mathcal {T}}_{k}^{*}\left( C_{j}\right)$ is a preference relation for all $C_{j}\in C$ then $\left( {\mathcal {T}}_{k}^{*},C\right)$ is called a soft preference relation corresponding to the decision attributes $D_{k},k=1,2,...,\left| D\right| ,$ where

$$\begin{aligned} {\mathcal {T}}_{k}^{*}\left( C_{j}\right) =\left\{ \begin{array}{c} b_{m} \succeq b_{n}\mid f\left( b_{m},C_{j}\right) \ge f\left( b_{n},C_{j}\right) \,\text {or}\,f\left( b_{m},C_{j}\right) =*\,\text {or}\, f\left( b_{n},C_{j}\right) =*\\ \,\text {and}\,g\left( b_{m},D_{k}\right) \ge g\left( b_{n},D_{k}\right) \,\text {or}\,g\left( b_{m},D_{k}\right) =*\,\text {or}\,g\left( b_{n},D_{k}\right) =*\end{array}\right\} . \end{aligned}$$

Denote $b_{m} \succeq b_{n}$ by $\left( b_{m},b_{n}\right)$. In an incomplete multi attributes with incomplete multi decisions information system ${\mathcal {S}}^{*}=\left( A,C,D,E\right) ,$ we answer to the first question of Deja’s which is related to conflict analysis model, that is,“*What are the intrinsic reasons for the conflict* ?” This means which symptoms are focused/treated by every medicine and have different attitudes in a conflict corresponding to a specific doctor. For any symptom $C_{j}\in C,$ we obtain attitude information for every medicine with respect to symptom $C_{j}\in C$ by using ${\mathcal {T}}_{k}^{*}\left( C_{j}\right)$ which is a preference relation corresponding to doctor $D_{k},$ where $j=1,2,...,\left| C\right|$ and $k=1,2,...,\left| D\right| .$ Thus we have answered the first question of Deja’s which is related to conflict analysis model.

### Definition 2

Let ${\mathcal {I}}{\mathcal {S}}^{*}=\left( A,C,D,E\right)$ be an incomplete information system and $\left( {\mathcal {T}}_{k}^{*},C\right)$ be a soft preference relation corresponding to the doctor $D_{k},k=1,2,...,\left| D\right|$ over *A*. Then there is dominance relation associated with which can be denoted by ${\mathcal {D}}ominan\left( {\mathcal {T}}_{k}^{*},C\right)$ and is defined by:

$$\begin{aligned} {\mathcal {D}}ominan\left( {\mathcal {T}}_{k}^{*},C\right) =\bigcap _{C_{j}\in C}{\mathcal {T}}_{k}^{*}\left( C_{j}\right) \end{aligned}$$

where $j=1,2,...,\left| C\right|$ and $k=1,2,...,\left| D\right| .$ It is worth mentioning that in an incomplete information system ${\mathcal {I}}{\mathcal {S}}^{*},$ the ${\mathcal {D}}ominan\left( {\mathcal {T}}_{k}^{*},C\right)$ depicts that which medicine(s) is/are preferred by a doctor $\left( D_{k}\right)$ on all symptoms by other medicine. But still we do not have sufficient information about the medicine(s) on which all doctors(s) are agree. So by Deja’s second question which is related to conflict analysis, *“How can a feasible consensus strategy be found” ?* which means that to find medicine(s) on which all the doctors or majority of doctors agreed.

### Definition 3

Let ${\mathcal {I}}{\mathcal {S}}^{*}=\left( A,C,D,E\right)$ be an incomplete information system and ${\mathcal {D}}ominan\left( {\mathcal {T}} _{k}^{*}, C\right)$ be a soft dominance relation corresponding to $D_{k}$ . For any $b\in A,$ define the soft dominance classes by

$$\begin{aligned} \left[ b\right] _{{\mathcal {D}}ominan\left( {\mathcal {T}}_{k}^{*}, C\right) }^{+}=\left\{ b_{i}\in A:b_{i}\succeq _{{\mathcal {D}}ominan\left( {\mathcal {T}}_{k}^{*},C\right) }b\right\} \end{aligned}$$

and

$$\begin{aligned} \left[ b\right] _{{\mathcal {D}}ominan\left( {\mathcal {T}}_{k}^{*}, C\right) }^{-}=\left\{ b_{i}\in A:b_{i}\preceq _{{\mathcal {D}}ominan\left( {\mathcal {T}}_{k}^{*},C\right) }b\right\} \end{aligned}$$

which represent $\left( {\mathcal {T}}_{k}^{*}, C\right)$-dominating set and $\left( {\mathcal {T}}_{k}^{*}, C\right)$-dominated set with respect to *b*, over all symptoms *C* corresponding to doctors $D_{k},$ $k=1,2,...,\left| D\right|$ of ${\mathcal {I}}{\mathcal {S}}^{*}$ respectively. The class $\left[ b\right] _{{\mathcal {D}}ominan\left( {\mathcal {T}} _{k}^{*},C\right) }^{+}$ describes the set of medicine that dominate ‘*b* ’ and $\left[ b\right] _{{\mathcal {D}}ominan\left( {\mathcal {T}}_{k}^{*}, C\right) }^{-}$ gives the set of medicine that dominated by ‘*b* ’ in terms of ${\mathcal {D}}ominan\left( {\mathcal {T}}_{k}^{*}, C\right) .$ We now discuss same properties and applications of $\left( {\mathcal {T}}_{k}^{*},C\right)$-dominating and $\left( {\mathcal {T}} _{k}^{*},C\right)$-dominated sets, where

$$\begin{aligned} {\mathcal {D}}ominan\left( {\mathcal {T}}_{k}^{*},C\right) ^{+} =\left\{ b\in A:\left[ b\right] _{{\mathcal {D}}ominan \left( {\mathcal {T}}_{k}^{*},C\right) }^{+}\right\} \end{aligned}$$

and

$$\begin{aligned} {\mathcal {D}}ominan\left( {\mathcal {T}}_{k}^{*},C\right) ^{-} =\left\{ b\in A:\left[ b\right] _{{\mathcal {D}}ominan \left( {\mathcal {T}}_{k}^{*},C\right) }^{-}\right\} . \end{aligned}$$

### Theorem 1

Let ${\mathcal {I}}{\mathcal {S}}^{*}=\left( A,C,D,E\right)$ be the given incomplete information system and ${\mathcal {D}}ominan\left( {\mathcal {T}} _{k}^{*},C\right)$ be a soft dominance relation corresponding to $D_{k},k=1,2,...,\left| D\right|$ over *A*. Then the following hold:

1. If $$C_{1}\subseteq C,$$ then $${\mathcal {D}}ominan\left( {\mathcal {T}}_{k}^{*},C\right) \subseteq {\mathcal {D}}ominan\left( {\mathcal {T}}_{k}^{*},C_{1}\right) ;$$
2. If $$b_{n}\in \left[ b_{m}\right] _{{\mathcal {D}}ominan\left( {\mathcal {T}}_{k}^{*},C\right) }^{+},$$ then $$\left[ b_{n}\right] _{{\mathcal {D}}ominan\left( {\mathcal {T}}_{k}^{*},C\right) }^{+}\subseteq \left[ b_{m}\right] _{{\mathcal {D}}ominan\left( {\mathcal {T}}_{k}^{*},C\right) }^{+};$$
3. $$\left[ b_{m}\right] _{{\mathcal {D}}ominan\left( {\mathcal {T}}_{k}^{*},C\right) }^{+}=\cup \left\{ \left[ b_{n}\right] _{ {\mathcal {D}}ominan\left( {\mathcal {T}}_{k}^{*},C\right) }^{+}:b_{n}\in \left[ b_{m}\right] _{{\mathcal {D}}ominan\left( {\mathcal {T}}_{k}^{*},C\right) }^{+}\right\} ;$$
4. $\left[ b_{m}\right] _{{\mathcal {D}}ominan\left( {\mathcal {T}}_{k}^{*},C\right) }^{+}=\left[ b_{n}\right] _{{\mathcal {D}}om inan\left( {\mathcal {T}}_{k}^{*},C\right) }^{+}$ iff $f\left( b_{m},C_{j}\right) =f\left( b_{n},C_{j}\right)$ or $f\left( b_{m},C_{j}\right) =*$ or $f\left( b_{n},C_{j}\right) =*$ and $g\left( b_{m},D_{k}\right) =g\left( b_{n},D_{k}\right)$ or $g\left( b_{m},D_{k}\right) =*$ or $g\left( b_{n},D_{k}\right) =*$ for all $C_{j}\in C$ and $D_{k}\in D.$

### Definition 4

Let ${\mathcal {I}}{\mathcal {S}}^{*}=\left( A,C,D,E\right)$ be an incomplete information system. We give the lower and upper approximations of the ${\mathcal {D}}ominan\left( {\mathcal {T}}_{k}^{*},C\right) ^{+}$ dominating set and ${\mathcal {D}}ominan\left( {\mathcal {T}}_{k}^{*},C\right) ^{-}$ dominated set over the incomplete information system. Then for any $S^{\Diamond }\subseteq A$ the lower and upper approximations with respect to ${\mathcal {D}}ominan\left( {\mathcal {T}}_{k}^{*},C\right) ^{+}$ dominating set and ${\mathcal {D}}ominan\left( {\mathcal {T}}_{k}^{*},C\right) ^{-}$ dominated set are:

$$\begin{aligned}&\left( \underline{S^{\Diamond }}\right) _{{\mathcal {D}}ominan\left( {\mathcal {T}}_{k}^{*},C\right) ^{+}}=\cup \left\{ \left[ b\right] _{ {\mathcal {D}}ominan\left( {\mathcal {T}}_{k}^{*},C\right) }^{+}: \left[ b\right] _{{\mathcal {D}}ominan\left( {\mathcal {T}}_{k}^{*},C\right) }^{+}\subseteq S^{\Diamond }\right\} \\&\left( \overline{S^{\Diamond }}\right) _{{\mathcal {D}}ominan\left( {\mathcal {T}}_{k}^{*},C\right) ^{+}}=\left( \left( \underline{S^{\Diamond c}}\right) _{{\mathcal {D}}ominan\left( {\mathcal {T}}_{k}^{*},C\right) ^{+}}\right) ^{c}. \end{aligned}$$

If $\left( \overline{S^{\Diamond }}\right) _{{\mathcal {D}}ominan\left( {\mathcal {T}}_{k}^{*},C\right) ^{+}}=\left( \underline{ S^{\Diamond }}\right) _{{\mathcal {D}}ominan\left( {\mathcal {T}} _{k}^{*},C\right) ^{+}},$ then it is called soft definable, otherwise soft rough set. Similarly

$$\begin{aligned}&\left( \underline{S^{\Diamond }}\right) _{{\mathcal {D}}ominan\left( {\mathcal {T}}_{k}^{*},C\right) ^{-}}=\cup \left\{ \left[ b\right] _{ {\mathcal {D}}ominan\left( {\mathcal {T}}_{k}^{*},C\right) }^{-}: \left[ b\right] _{{\mathcal {D}}ominan\left( {\mathcal {T}}_{k}^{*},C\right) }^{-}\subseteq S^{\Diamond }\right\} \\&\left( \overline{S^{\Diamond }}\right) _{{\mathcal {D}}ominan\left( {\mathcal {T}}_{k}^{*},C\right) ^{-}}=\left( \left( \underline{S^{\Diamond c}}\right) _{{\mathcal {D}}ominan\left( {\mathcal {T}}_{k}^{*},C\right) ^{-}}\right) ^{c}. \end{aligned}$$

If $\left( \overline{S^{\Diamond }}\right) _{{\mathcal {D}}ominan\left( {\mathcal {T}}_{k}^{*},C\right) ^{-}}=\left( \underline{ S^{\Diamond }}\right) _{{\mathcal {D}}ominan\left( {\mathcal {T}} _{k}^{*},C\right) ^{-}},$ then it is called soft definable, otherwise it is called soft rough set, where $S^{\Diamond }$ denote the complementary set $A-S^{\Diamond }$ of $S^{\Diamond }\subseteq A.$

### Theorem 2

Let ${\mathcal {I}}{\mathcal {S}}^{*}=\left( A,C,D,E\right)$ be an incomplete information system and $S^{\Diamond }\subseteq A.$ Then

1. $$\left( \overline{\left( \overline{S^{\Diamond }}\right) } \right) _{{\mathcal {D}}ominan\left( {\mathcal {T}}_{k}^{*},C\right) ^{+}}=\left( \overline{S^{\Diamond }}\right) _{{\mathcal {D}}ominan \left( {\mathcal {T}}_{k}^{*},C\right) ^{+}}$$
2. $$\left( \underline{\left( \underline{S^{\Diamond }} \right) }\right) _{{\mathcal {D}}ominan\left( {\mathcal {T}}_{k}^{*},C\right) ^{+}}=\left( \underline{S^{\Diamond }}\right) _{{\mathcal {D}}om inan\left( {\mathcal {T}}_{k}^{*},C\right) ^{+}}.$$

In classical rough set theory, if the lower approximation $R\left( \underline{S^{\Diamond }}\right)$ and upper approximation $R\left( \overline{S^{\Diamond }}\right)$ of the set $S^{\Diamond }$ are equal to $S^{\Diamond },$ then $S^{\Diamond }$ is called definable, otherwise $S^{\Diamond }$ is considered a rough set. So both the approximations $R\left( \underline{S^{\Diamond }}\right)$ and $R\left( \overline{ S^{\Diamond }}\right)$ of the set $S^{\Diamond }$ in Pawlak’s rough set model are definable sets. That is, $R\left( \underline{R\left( \underline{ S^{\Diamond }}\right) }\right) =R\left( \underline{S^{\Diamond }}\right) =R\left( \overline{R\left( \underline{S^{\Diamond }}\right) }\right) ,$ $R\left( \underline{R\left( \overline{S^{\Diamond }}\right) }\right) =R\left( \overline{S^{\Diamond }}\right) =R\left( \overline{R\left( \overline{ S^{\Diamond }}\right) }\right) .$ But in soft dominance based rough sets, the lower and upper approximations are hardly definable sets. In general, they are still rough sets. That is, $\left( \underline{\left( \underline{ S^{\Diamond }}\right) }\right) _{{\mathcal {D}}ominan\left( {\mathcal {T}} _{k}^{*},C\right) ^{+}}=\left( \underline{S^{\Diamond }}\right) _{ {\mathcal {D}}ominan\left( {\mathcal {T}}_{k}^{*},C\right) ^{+}}\ne \left( \overline{\left( \underline{S^{\Diamond }}\right) }\right) _{{\mathcal {D}}ominan\left( {\mathcal {T}}_{k}^{*},C\right) ^{+}},$ $\left( \overline{\left( \overline{S^{\Diamond }}\right) }\right) _{{\mathcal {D}}om inan\left( {\mathcal {T}}_{k}^{*},C\right) ^{+}}=\left( \overline{ S^{\Diamond }}\right) _{{\mathcal {D}}ominan\left( {\mathcal {T}} _{k}^{*},C\right) ^{+}}\ne \left( \underline{\left( \overline{ S^{\Diamond }}\right) }\right) _{{\mathcal {D}}ominan\left( {\mathcal {T}} _{k}^{*},C\right) ^{+}}.$ We now give an example to show this fact.

### Example 1

Let ${\mathcal {I}}{\mathcal {S}}^{*}:=\left( A,C,D,E\right)$ be an incomplete multi attributes with incomplete multi decisions information system. For the corona virus (COVID-19) conflict situation, with five symptoms $C_{j}$ where $j=1,...,\left| C\right| =5$ and four doctors $D_{k},$ $k=1,2,...,\left| D\right| =4$. The system comprises of twelve medicines $b_{i},$ $i=1,2,...,\left| A\right| =12$ pre-established by experts or obtained by other approaches and that every agent has given its preference in advance for every medicine. The results are presented in Table 1. Construct soft preference relations for the symptoms corresponding to doctors, $\left( {\mathcal {T}}_{1}^{*},C\right)$ corresponding to $D_{1}.$ Applying Definitions 1 and 2 to construct soft dominance relations.

$${\mathcal {D}}ominan\left( {\mathcal {T}}_{1}^{*},C\right) =\left\{ \begin{array}{c} \left( b_{1},b_{1}\right) ,\ \left( b_{2},b_{2}\right) ,\ \left( b_{3},b_{3}\right) ,\ \left( b_{4},b_{4}\right) ,\ \left( b_{5},b_{5}\right) ,\ \left( b_{6},b_{6}\right) , \\ \ \left( b_{7},b_{7}\right) ,\left( b_{8},b_{8}\right) ,\ \left( b_{9},b_{9}\right) ,\ \left( b_{10},b_{10}\right) ,\ \left( b_{11},b_{11}\right) ,\ \left( b_{12},b_{12}\right) , \\ \left( b_{1},b_{2}\right) ,\ \left( b_{2},b_{4}\right) ,\ \left( b_{2},b_{8}\right) ,\ \left( b_{2},b_{10}\right) ,\ \left( b_{3},b_{1}\right) ,\ \left( b_{3},b_{4}\right) , \\ \left( b_{3},b_{5}\right) ,\ \left( b_{3},b_{6}\right) ,\ \left( b_{3},b_{8}\right) ,\ \left( b_{3},b_{10}\right) ,\ \left( b_{3},b_{11}\right) ,\ \left( b_{6},b_{2}\right) , \\ \left( b_{6},b_{4}\right) ,\ \left( b_{7},b_{1}\right) ,\ \left( b_{7},b_{2}\right) ,\ \left( b_{7},b_{3}\right) ,\ \left( b_{7},b_{4}\right) ,\ \left( b_{7},b_{5}\right) , \\ \left( b_{7},b_{6}\right) ,\left( b_{8},b_{5}\right) ,\ \left( b_{8},b_{10}\right) ,\left( b_{8},b_{11}\right) ,\ \left( b_{9},b_{1}\right) ,\ \left( b_{9},b_{2}\right) , \\ \left( b_{9},b_{4}\right) ,\ \left( b_{9},b_{5}\right) ,\ \left( b_{9},b_{8}\right) ,\ \left( b_{9},b_{10}\right) ,\ \left( b_{9},b_{11}\right) ,\ \left( b_{9},b_{12}\right) , \\ \left( b_{10},b_{4}\right) ,\ \left( b_{10},b_{8}\right) ,\ \left( b_{11},b_{10}\right) ,\ \left( b_{12},b_{10}\right) ,\ \left( b_{12},b_{11}\right) \end{array}\right\} .$$

The soft dominance classes from ${\mathcal {D}}ominan\left( {\mathcal {T}} _{1}^{*},C\right)$ are given in the following Table 2.

For $S^{\Diamond }=\left\{ b_{1},b_{2},b_{3},b_{4},b_{6},b_{7},b_{9},b_{10},b_{12}\right\} ,$ the approximations $\left( \underline{S^{\Diamond }}\right) _{{\mathcal {D}}om inan\left( {\mathcal {T}}_{k}^{*},C\right) ^{+}}=\left( \underline{ \left( \underline{S^{\Diamond }}\right) }\right) _{{\mathcal {D}}ominan\left( {\mathcal {T}}_{k}^{*},C\right) ^{+}}=\left\{ b_{1},b_{2},b_{3},b_{4},b_{6},b_{7},b_{9},b_{10},b_{12}\right\}$ and $\left( \overline{\left( \underline{S^{\Diamond }}\right) }\right) _{{\mathcal {D}}ominan\left( {\mathcal {T}}_{k}^{*},C\right) ^{+}}=\left\{ b_{1},b_{2},b_{3},b_{4},b_{5},b_{6},b_{7},b_{8},b_{9},b_{10},b_{11,}b_{12} \right\} .$

Therefore $\left( \underline{\left( \underline{S^{\Diamond }}\right) } \right) _{{\mathcal {D}}ominan\left( {\mathcal {T}}_{k}^{*},C\right) ^{+}}=\left( \underline{S^{\Diamond }}\right) _{{\mathcal {D}}ominan \left( {\mathcal {T}}_{k}^{*},C\right) ^{+}}\ne \left( \overline{\left( \underline{S^{\Diamond }}\right) }\right) _{{\mathcal {D}}ominan\left( {\mathcal {T}}_{k}^{*},C\right) ^{+}}.$ Similarly we can easily find that

$$\left( \overline{\left( \overline{S^{\Diamond }}\right) }\right) _{{\mathcal {D}}ominan\left( {\mathcal {T}}_{k}^{*},C\right) ^{+}}=\left( \overline{S^{\Diamond }}\right) _{{\mathcal {D}}ominan\left( {\mathcal {T}}_{k}^{*},C\right) ^{+}}\ne \left( \underline{\left( \overline{ S^{\Diamond }}\right) }\right) _{{\mathcal {D}}ominan\left( {\mathcal {T}} _{k}^{*},C\right) ^{+}}.$$

**Table 2:** Dominating classes $\left[ b_{i}\right] _{{\mathcal {D}}ominan \left( {\mathcal {T}}_{1}^{*},C\right) }^{+}$ and Dominated classes $\left[ b_{i}\right] _{{\mathcal {D}}omian \left( {\mathcal {T}}_{1}^{*},C\right) }^{-}$.

$b_{i}$	$\left[ b_{i}\right] _{{\mathcal {D}}ominan \left( {\mathcal {T}}_{1}^{*},C\right) }^{+}$	$\left[ b_{i}\right] _{{\mathcal {D}}ominan\left( {\mathcal {T}} _{1}^{*},C\right) }^{-}$
$b_{1}$	$\left\{ b_{1},b_{3},b_{7},b_{9}\right\}$	$\left\{ b_{1},b_{2}\right\}$
$b_{2}$	$\left\{ b_{1,}b_{2},b_{6},b_{7},b_{9}\right\}$	$\left\{ b_{2},b_{4},b_{8},b_{10},b_{11}\right\}$
$b_{3}$	$\left\{ b_{3},b_{7}\right\}$	$\left\{ b_{1},b_{3},b_{4},b_{5},b_{6},b_{8},b_{10},b_{11}\right\}$
$b_{4}$	$\left\{ b_{2},b_{3},b_{4},b_{6},b_{7},b_{9},b_{10}\right\}$	$\left\{ b_{4}\right\}$
$b_{5}$	$\left\{ b_{3},b_{5},b_{7},b_{8},b_{9}\right\}$	$\left\{ b_{5}\right\}$
$b_{6}$	$\left\{ b_{6},b_{7}\right\}$	$\left\{ b_{2},b_{4},b_{6}\right\}$
$b_{7}$	$\left\{ b_{7}\right\}$	$\left\{ b_{1},b_{2},b_{3},b_{4},b_{5,}b_{6},b_{7}\right\}$
$b_{8}$	$\left\{ b_{3},b_{8},b_{10}\right\}$	$\left\{ b_{5},b_{8},b_{10},b_{11}\right\}$
$b_{9}$	$\left\{ b_{9}\right\}$	$\left\{ b_{1},b_{2},b_{4},b_{5},b_{8},b_{9},b_{10},b_{11},b_{12}\right\}$
$b_{10}$	$\left\{ b_{2},b_{3},b_{8},b_{9},b_{10},b_{11},b_{12}\right\}$	$\left\{ b_{4},b_{8},b_{10}\right\}$
$b_{11}$	$\left\{ b_{2},b_{3},b_{8},b_{9},b_{11},b_{12}\right\}$	$\left\{ b_{10},b_{11}\right\}$
$b_{12}$	$\left\{ b_{9},b_{12}\right\}$	$\left\{ b_{10},b_{11},b_{12}\right\}$

From the above example we see that $\left( \underline{X^{\Diamond }}\right) _{{\mathcal {D}}ominan\left( {\mathcal {T}}_{k}^{*},C\right) ^{+}}$ and $\left( \overline{S^{\Diamond }}\right) _{{\mathcal {D}}ominan \left( {\mathcal {T}}_{k}^{*},C\right) ^{+}}$ are still rough sets, if we apply the lower or upper approximation operations over and over again to a subset $S^{\Diamond }$ of *A*,  we obtain six different sets at most. These sets are $\left( \underline{S^{\Diamond }}\right) _{{\mathcal {D}}ominan\left( {\mathcal {T}}_{k}^{*},C\right) ^{+}},$ $\left( \overline{\left( \underline{S^{\Diamond }}\right) }\right) _{{\mathcal {D}}ominan\left( {\mathcal {T}}_{k}^{*},C\right) ^{+}},$ $\left( \underline{\left( \overline{ \left( \underline{S^{\Diamond }}\right) }\right) }\right) _{{\mathcal {D}}om inan\left( {\mathcal {T}}_{k}^{*},C\right) ^{+}},$ $\left( \overline{S^{\Diamond }}\right) _{{\mathcal {D}}ominan\left( {\mathcal {T}}_{k}^{*},C\right) ^{+}},$ $\left( \underline{\left( \overline{ S^{\Diamond }}\right) }\right) _{{\mathcal {D}}ominan\left( {\mathcal {T}} _{k}^{*},C\right) ^{+}},$ and $\left( \overline{\left( \underline{\left( \overline{S^{\Diamond }}\right) }\right) }\right) _{{\mathcal {D}}ominan\left( {\mathcal {T}}_{k}^{*},C\right) ^{+}}.$

Now we give

### Theorem 3

Let ${\mathcal {I}}{\mathcal {S}}^{*}=\left( A,C,D,E\right)$ be an incomplete information system and $S^{\Diamond }\subseteq A.$ Then the following hold:

$$\left( 1\right) $$ $$\left( \overline{\left( \underline{\left( \overline{ \left( \underline{S^{\Diamond }}\right) }\right) }\right) }\right) _{ {\mathcal {D}}ominan\left( {\mathcal {T}}_{k}^{*},C\right) ^{+}}=\left( \overline{\left( \underline{S^{\Diamond }}\right) }\right) _{ {\mathcal {D}}ominan\left( {\mathcal {T}}_{k}^{*},C\right) ^{+}};$$

$$\left( 2\right) $$ $$\left( \underline{\left( \overline{\left( \underline{ \left( \overline{S^{\Diamond }}\right) }\right) }\right) }\right) _{{\mathcal {D}}ominan\left( {\mathcal {T}}_{k}^{*},C\right) ^{+}}=\left( \underline{\left( \overline{S^{\Diamond }}\right) }\right) _{{\mathcal {D}}om inan\left( {\mathcal {T}}_{k}^{*},C\right) ^{+}}.$$

### Proof

$\left( 1\right)$ As we know that $\left( \underline{S^{\Diamond }}\right) _{{\mathcal {D}}ominan\left( {\mathcal {T}}_{k}^{*},C\right) ^{+}}\subseteq \left( \overline{\left( \underline{S^{\Diamond }}\right) } \right) _{{\mathcal {D}}ominan\left( {\mathcal {T}}_{k}^{*},C\right) ^{+}}.$ This implies that

$\left( \underline{\left( \underline{S^{\Diamond }}\right) }\right) _{ {\mathcal {D}}ominan\left( {\mathcal {T}}_{k}^{*},C\right) ^{+}}\subseteq \left( \underline{\left( \overline{\left( \underline{ S^{\Diamond }}\right) }\right) }\right) _{{\mathcal {D}}ominan\left( {\mathcal {T}}_{k}^{*},C\right) ^{+}}.$ Therefore $\left( \underline{ S^{\Diamond }}\right) _{{\mathcal {D}}ominan\left( {\mathcal {T}} _{k}^{*},C\right) ^{+}}\subseteq \left( \underline{\left( \overline{ \left( \underline{S^{\Diamond }}\right) }\right) }\right) _{{\mathcal {D}}om inan\left( {\mathcal {T}}_{k}^{*},C\right) ^{+}}.$ Also $\left( \overline{\left( \underline{S^{\Diamond }}\right) }\right) _{{\mathcal {D}}om inan\left( {\mathcal {T}}_{k}^{*},C\right) ^{+}}\subseteq \left( \overline{\left( \underline{\left( \overline{\left( \underline{S^{\Diamond }} \right) }\right) }\right) }\right) _{{\mathcal {D}}ominan\left( {\mathcal {T}}_{k}^{*},C\right) ^{+}}.$ Now $\left( \underline{\left( \overline{\left( \underline{S^{\Diamond }}\right) }\right) }\right) _{ {\mathcal {D}}ominan\left( {\mathcal {T}}_{k}^{*},C\right) ^{+}}\subseteq \left( \overline{\left( \underline{S^{\Diamond }}\right) } \right) _{{\mathcal {D}}ominan\left( {\mathcal {T}}_{k}^{*},C\right) ^{+}}.$ This implies that

$$\left( \overline{\left( \underline{\left( \overline{\left( \underline{ S^{\Diamond }}\right) }\right) }\right) }\right) _{{\mathcal {D}}ominan\left( {\mathcal {T}}_{k}^{*},C\right) ^{+}}\subseteq \left( \overline{ \left( \overline{\left( \underline{S^{\Diamond }}\right) }\right) }\right) _{ {\mathcal {D}}ominan\left( {\mathcal {T}}_{k}^{*},C\right) ^{+}}=\left( \overline{\left( \underline{S^{\Diamond }}\right) }\right) _{ {\mathcal {D}}ominan\left( {\mathcal {T}}_{k}^{*},C\right) ^{+}}.$$ Therefore $$\left( \overline{\left( \underline{\left( \overline{\left( \underline{S^{\Diamond }}\right) }\right) }\right) }\right) _{{\mathcal {D}}om inan\left( {\mathcal {T}}_{k}^{*},C\right) ^{+}}=\left( \overline{ \left( \underline{S^{\Diamond }}\right) }\right) _{{\mathcal {D}}ominan\left( {\mathcal {T}}_{k}^{*},C\right) ^{+}}.$$

$\left( 2\right)$ Since $\left( \underline{\left( \overline{S^{\Diamond }} \right) }\right) _{{\mathcal {D}}ominan\left( {\mathcal {T}}_{k}^{*},C\right) ^{+}}\subseteq \left( \overline{S^{\Diamond }}\right) _{{\mathcal {D}}ominan\left( {\mathcal {T}}_{k}^{*},C\right) ^{+}},$ so $\left( \overline{\left( \underline{\left( \overline{S^{\Diamond }}\right) }\right) } \right) _{{\mathcal {D}}ominan\left( {\mathcal {T}}_{k}^{*},C\right) ^{+}}\subseteq \left( \overline{\left( \overline{S^{\Diamond }}\right) } \right) _{{\mathcal {D}}ominan\left( {\mathcal {T}}_{k}^{*},C\right) ^{+}}=\left( \overline{S^{\Diamond }}\right) _{{\mathcal {D}}ominan\left( {\mathcal {T}}_{k}^{*},C\right) ^{+}}.$ This implies that $\left( \underline{\left( \overline{\left( \underline{\left( \overline{S^{\Diamond }} \right) }\right) }\right) }\right) _{{\mathcal {D}}ominan\left( {\mathcal {T}}_{k}^{*},C\right) ^{+}}\subseteq \left( \underline{\left( \overline{S^{\Diamond }}\right) }\right) _{{\mathcal {D}}ominan\left( {\mathcal {T}}_{k}^{*},C\right) ^{+}}.$ It follows from that $\left( \underline{\left( \overline{S^{\Diamond }}\right) }\right) _{{\mathcal {D}}om inan\left( {\mathcal {T}}_{k}^{*},C\right) ^{+}}\subseteq \left( \overline{\left( \underline{\left( \overline{S^{\Diamond }}\right) }\right) } \right) _{{\mathcal {D}}ominan\left( {\mathcal {T}}_{k}^{*},C\right) ^{+}}.$ Also $\left( \underline{\left( \underline{\left( \overline{ S^{\Diamond }}\right) }\right) }\right) _{{\mathcal {D}}ominan\left( {\mathcal {T}}_{k}^{*},C\right) ^{+}}\subseteq \left( \underline{\left( \overline{\left( \underline{\left( \overline{S^{\Diamond }}\right) }\right) } \right) }\right) _{{\mathcal {D}}ominan\left( {\mathcal {T}}_{k}^{*},C\right) ^{+}}.$ This implies that $\left( \underline{\left( \overline{ S^{\Diamond }}\right) }\right) _{{\mathcal {D}}ominan\left( {\mathcal {T}} _{k}^{*},C\right) ^{+}}\subseteq \left( \underline{\left( \overline{ \left( \underline{\left( \overline{S^{\Diamond }}\right) }\right) }\right) } \right) _{{\mathcal {D}}ominan\left( {\mathcal {T}}_{k}^{*},C\right) ^{+}}.$ Therefore $\left( \underline{\left( \overline{\left( \underline{ \left( \overline{S^{\Diamond }}\right) }\right) }\right) }\right) _{{\mathcal {D}}ominan\left( {\mathcal {T}}_{k}^{*},C\right) ^{+}}=\left( \underline{\left( \overline{S^{\Diamond }}\right) }\right) _{{\mathcal {D}}om inan\left( {\mathcal {T}}_{k}^{*},C\right) ^{+}}.$ $\square$

The following theorem gives the relationship between the aforesaid six sets.

### Theorem 4

Let ${\mathcal {I}}{\mathcal {S}}^{*}=\left( A,C,D,E\right)$ be an incomplete information system and $S^{\Diamond }\subseteq A.$ Then the following hold:

1. $$\left( \underline{\left( \overline{\left( \underline{ \left( \overline{S^{\Diamond }}\right) }\right) }\right) }\right) _{{\mathcal {D}}ominan\left( {\mathcal {T}}_{k}^{*},C\right) ^{+}}=\left( \underline{\left( \overline{S^{\Diamond }}\right) }\right) _{{\mathcal {D}}om inan\left( {\mathcal {T}}_{k}^{*},C\right) ^{+}}=\left( \underline{ \left( \underline{\left( \overline{S^{\Diamond }}\right) }\right) }\right) _{ {\mathcal {D}}ominan\left( {\mathcal {T}}_{k}^{*},C\right) ^{+}};$$
2. $$\left( \overline{\left( \underline{\left( \overline{ \left( \underline{S^{\Diamond }}\right) }\right) }\right) }\right) _{ {\mathcal {D}}ominan\left( {\mathcal {T}}_{k}^{*},C\right) ^{+}}=\left( \overline{\left( \underline{S^{\Diamond }}\right) }\right) _{ {\mathcal {D}}ominan\left( {\mathcal {T}}_{k}^{*},C\right) ^{+}}=\left( \overline{\left( \overline{\left( \underline{S^{\Diamond }} \right) }\right) }\right) _{{\mathcal {D}}ominan\left( {\mathcal {T}} _{k}^{*},C\right) ^{+}};$$
3. If $$\left( \underline{S^{\Diamond }}\right) _{{\mathcal {D}} ominan\left( {\mathcal {T}}_{k}^{*},C\right) ^{+}}=\left( \overline{S^{\Diamond }}\right) _{{\mathcal {D}}ominan\left( {\mathcal {T}}_{k}^{*},C\right) ^{+}},$$ then $$\left( \underline{S^{\Diamond }}\right) _{{\mathcal {D}}ominan\left( {\mathcal {T}}_{k}^{*},C\right) ^{+}}=S^{\Diamond }=\left( \overline{S^{\Diamond }}\right) _{{\mathcal {D}}om inan\left( {\mathcal {T}}_{k}^{*},C\right) ^{+}}$$
  $$=\left( \underline{\left( \overline{S^{\Diamond }}\right) }\right) _{ {\mathcal {D}}ominan\left( {\mathcal {T}}_{k}^{*},C\right) ^{+}}=\left( \overline{\left( \underline{\left( \overline{S^{\Diamond }} \right) }\right) }\right) _{{\mathcal {D}}ominan\left( {\mathcal {T}} _{k}^{*},C\right) ^{+}}=\left( \overline{\left( \underline{S^{\Diamond }} \right) }\right) _{{\mathcal {D}}ominan\left( {\mathcal {T}}_{k}^{*},C\right) ^{+}}$$
  $$=\left( \underline{\left( \overline{\left( \underline{S^{\Diamond }}\right) }\right) }\right) _{{\mathcal {D}}ominan\left( {\mathcal {T}}_{k}^{*},C\right) ^{+}};$$
4. If $$\left( \overline{S^{\Diamond }}\right) _{{\mathcal {D}}om inan\left( {\mathcal {T}}_{k}^{*},C\right) ^{+}}=\left( \underline{ \left( \overline{\left( \underline{S^{\Diamond }}\right) }\right) }\right) _{ {\mathcal {D}}ominan\left( {\mathcal {T}}_{k}^{*},C\right) ^{+}},$$ then $$\left( \overline{S^{\Diamond }}\right) _{{\mathcal {D}}ominan \left( {\mathcal {T}}_{k}^{*},C\right) ^{+}}=\left( \underline{\left( \overline{S^{\Diamond }}\right) }\right) _{{\mathcal {D}}ominan\left( {\mathcal {T}}_{k}^{*},C\right) ^{+}}$$
  $$=\left( \overline{\left( \underline{\left( \overline{S^{\Diamond }}\right) } \right) }\right) _{{\mathcal {D}}ominan\left( {\mathcal {T}}_{k}^{*},C\right) ^{+}}=\left( \overline{\left( \underline{S^{\Diamond }}\right) } \right) _{{\mathcal {D}}ominan\left( {\mathcal {T}}_{k}^{*},C\right) ^{+}}=\left( \underline{\left( \overline{\left( \underline{S^{\Diamond }} \right) }\right) }\right) _{{\mathcal {D}}ominan\left( {\mathcal {T}} _{k}^{*},C\right) ^{+}};$$
5. If $$\left( \underline{S^{\Diamond }}\right) _{{\mathcal {D}} ominan\left( {\mathcal {T}}_{k}^{*},C\right) ^{+}}=\left( \overline{\left( \underline{\left( \overline{S^{\Diamond }}\right) }\right) } \right) _{{\mathcal {D}}ominan\left( {\mathcal {T}}_{k}^{*},C\right) ^{+}},$$ then $$\left( \underline{S^{\Diamond }}\right) _{{\mathcal {D}}om inan\left( {\mathcal {T}}_{k}^{*},C\right) ^{+}}=\left( \overline{\left( \underline{S^{\Diamond }}\right) }\right) _{{\mathcal {D}}ominan\left( {\mathcal {T}}_{k}^{*},C\right) ^{+}}$$
  $$=\left( \overline{\left( \underline{\left( \overline{S^{\Diamond }}\right) } \right) }\right) _{{\mathcal {D}}ominan\left( {\mathcal {T}}_{k}^{*},C\right) ^{+}}=\left( \underline{\left( \overline{S^{\Diamond }}\right) } \right) _{{\mathcal {D}}ominan\left( {\mathcal {T}}_{k}^{*},C\right) ^{+}}=\left( \underline{\left( \overline{\left( \underline{S^{\Diamond }} \right) }\right) }\right) _{{\mathcal {D}}ominan\left( {\mathcal {T}} _{k}^{*},C\right) ^{+}};$$
6. If $\left( \overline{S^{\Diamond }}\right) _{{\mathcal {D}}om inan\left( {\mathcal {T}}_{k}^{*},C\right) ^{+}}=\left( \overline{ \left( \underline{S^{\Diamond }}\right) }\right) _{{\mathcal {D}}ominan \left( {\mathcal {T}}_{k}^{*},C\right) ^{+}},$ then $\left( \overline{ S^{\Diamond }}\right) _{{\mathcal {D}}ominan\left( {\mathcal {T}} _{k}^{*},C\right) ^{+}}=\left( \overline{\left( \underline{S^{\Diamond }} \right) }\right) _{{\mathcal {D}}ominan\left( {\mathcal {T}}_{k}^{*},C\right) ^{+}}=\left( \overline{\left( \underline{\left( \overline{ S^{\Diamond }}\right) }\right) }\right) _{{\mathcal {D}}ominan\left( {\mathcal {T}}_{k}^{*},C\right) ^{+}}$ and $\left( \underline{\left( \overline{S^{\Diamond }}\right) }\right) _{{\mathcal {D}}ominan\left( {\mathcal {T}}_{k}^{*},C\right) ^{+}}=\left( \underline{\left( \overline{ \left( \underline{S^{\Diamond }}\right) }\right) }\right) _{{\mathcal {D}}om inan\left( {\mathcal {T}}_{k}^{*},C\right) ^{+}};$
7. If $\left( \underline{S^{\Diamond }}\right) _{{\mathcal {D}} ominan\left( {\mathcal {T}}_{k}^{*},C\right) ^{+}}=\left( \underline{\left( \overline{S^{\Diamond }}\right) }\right) _{{\mathcal {D}}om inan\left( {\mathcal {T}}_{k}^{*},C\right) ^{+}},$ then $\left( \underline{S^{\Diamond }}\right) _{{\mathcal {D}}ominan \left( {\mathcal {T}}_{k}^{*},C\right) ^{+}} =\left( \underline{\left( \overline{S^{\Diamond }}\right) }\right) _{{\mathcal {D}}ominan\left( {\mathcal {T}}_{k}^{*},C\right) ^{+}}=\left( \underline{\left( \overline{\left( \underline{ S^{\Diamond }}\right) }\right) }\right) _{{\mathcal {D}}ominan\left( {\mathcal {T}}_{k}^{*},C\right) ^{+}}$ and $\left( \overline{\left( \underline{S^{\Diamond }}\right) }\right) _{{\mathcal {D}}ominan\left( {\mathcal {T}}_{k}^{*},C\right) ^{+}}=\left( \overline{\left( \underline{ \left( \overline{S^{\Diamond }}\right) }\right) }\right) _{{\mathcal {D}}om inan\left( {\mathcal {T}}_{k}^{*},C\right) ^{+}};$
8. If $$\left( \underline{\left( \overline{\left( \underline{ S^{\Diamond }}\right) }\right) }\right) _{{\mathcal {D}}ominan\left( {\mathcal {T}}_{k}^{*},C\right) ^{+}}=\left( \overline{\left( \underline{ \left( \overline{S^{\Diamond }}\right) }\right) }\right) _{{\mathcal {D}}om inan\left( {\mathcal {T}}_{k}^{*},C\right) ^{+}},$$ then $$\left( \overline{\left( \underline{S^{\Diamond }}\right) }\right) _{{\mathcal {D}}om inan\left( {\mathcal {T}}_{k}^{*},C\right) ^{+}}=\left( \overline{ \left( \underline{\left( \overline{S^{\Diamond }}\right) }\right) }\right) _{ {\mathcal {D}}ominan\left( {\mathcal {T}}_{k}^{*},C\right) ^{+}}=\left( \underline{\left( \overline{S^{\Diamond }}\right) }\right) _{ {\mathcal {D}}ominan\left( {\mathcal {T}}_{k}^{*},C\right) ^{+}}=\left( \underline{\left( \overline{\left( \underline{S^{\Diamond }} \right) }\right) }\right) _{{\mathcal {D}}ominan\left( {\mathcal {T}} _{k}^{*},C\right) ^{+}};$$
9. If $$\left( \overline{\left( \underline{S^{\Diamond }} \right) }\right) _{{\mathcal {D}}ominan\left( {\mathcal {T}}_{k}^{*},C\right) ^{+}}=\left( \underline{\left( \overline{S^{\Diamond }}\right) } \right) _{{\mathcal {D}}ominan\left( {\mathcal {T}}_{k}^{*},C\right) ^{+}},$$ then $$\left( \overline{\left( \underline{S^{\Diamond }}\right) } \right) _{{\mathcal {D}}ominan\left( {\mathcal {T}}_{k}^{*},C\right) ^{+}}=\left( \overline{\left( \underline{\left( \overline{S^{\Diamond }} \right) }\right) }\right) _{{\mathcal {D}}ominan\left( {\mathcal {T}} _{k}^{*},C\right) ^{+}}=\left( \underline{\left( \overline{S^{\Diamond }} \right) }\right) _{{\mathcal {D}}ominan\left( {\mathcal {T}}_{k}^{*},C\right) ^{+}}=\left( \underline{\left( \overline{\left( \underline{ S^{\Diamond }}\right) }\right) }\right) _{{\mathcal {D}}ominan\left( {\mathcal {T}}_{k}^{*},C\right) ^{+}}.$$

## Logical multigranulation rough sets using soft dominance approach

According to two different approximations, Qian et al.^50,65^ developed two different multigranulation rough sets including optimistic and pessimistic ones.

### Logical disjunction soft dominating/dominated optimistic multigranulation rough sets

#### Definition 5

Let ${\mathcal {I}}{\mathcal {S}}^{*}=\left( A,C,D,E\right)$ be an incomplete information system. If $\beta \in \left( 0.5,1\right]$ and $l\in {\mathbb {N}} \cup \{0\},$ then for any $S^{\Diamond }\subseteq A,$ the optimistic lower approximation and optimistic upper approximation of $S^{\Diamond }$ with respect to ${\mathcal {D}}ominan\left( {\mathcal {T}}_{k}^{*},C\right) ^{+}$ are defined by

$$\begin{aligned} \sum \limits _{k=1}^{\left| D\right| }\left( \underline{S^{\Diamond }} \right) _{{\mathcal {D}}ominan\left( {\mathcal {T}}_{k}^{*},C\right) ^{+}}^{\beta \vee l\left( o\right) }=\bigvee \limits _{k=1}^{\left| D\right| }\left\{ \begin{array}{c} b\in A\mid \left( \frac{\left| \left[ b\right] _{{\mathcal {D}}ominan\left( {\mathcal {T}}_{k}^{*},C\right) }^{+}\cap S^{\Diamond }\right| }{\left| \left[ b\right] _{{\mathcal {D}}ominan\left( {\mathcal {T}} _{k}^{*},C\right) }^{+}\right| }\right) >\beta \\ \vee \left( \left| \left[ b\right] _{{\mathcal {D}}ominan\left( {\mathcal {T}}_{k}^{*},C\right) }^{+}\right| -\left| \left[ b\right] _{{\mathcal {D}}ominan\left( {\mathcal {T}}_{k}^{*},C\right) }^{+}\cap S^{\Diamond }\right| \right) <l \end{array}\right\} ; \end{aligned}$$

and

$$\begin{aligned} \sum \limits _{k=1}^{\left| D\right| }\left( \overline{S^{\Diamond }} \right) _{{\mathcal {D}}ominan\left( {\mathcal {T}}_{k}^{*},C\right) ^{+}}^{\beta \vee l\left( o\right) }=\bigwedge \limits _{k=1}^{\left| D\right| }\left\{ \begin{array}{c} b\in A\mid \left( \frac{\left| \left[ b\right] _{{\mathcal {D}}ominan \left( {\mathcal {T}}_{k}^{*},C\right) }^{+}\cap S^{\Diamond }\right| }{\left| \left[ b\right] _{{\mathcal {D}}ominan\left( {\mathcal {T}} _{k}^{*},C\right) }^{+}\right| }\right) \ge 1-\beta \\ \vee \left( \left| \left[ b\right] _{{\mathcal {D}}ominan\left( {\mathcal {T}}_{k}^{*},C\right) }^{+}\cap S^{\Diamond }\right| \right) \ge l \end{array}\right\} . \end{aligned}$$

The pair $\left( \sum \nolimits _{k=1}^{\left| D\right| }\left( \underline{S^{\Diamond }}\right) _{{\mathcal {D}}ominan\left( {\mathcal {T}}_{k}^{*},C\right) ^{+}}^{\beta \vee l\left( o\right) },\sum \nolimits _{k=1}^{\left| D\right| }\left( \overline{S^{\Diamond }} \right) _{{\mathcal {D}}ominan\left( {\mathcal {T}}_{k}^{*},C\right) ^{+}}^{\beta \vee l\left( o\right) }\right)$ is called the logical disjunction soft dominating optimistic multigranulation rough set if $\sum \nolimits _{k=1}^{\left| D\right| }\left( \underline{ S^{\Diamond }}\right) _{{\mathcal {D}}ominan\left( {\mathcal {T}} _{k}^{*},C\right) ^{+}}^{\beta \vee l\left( o\right) }\ne \sum \nolimits _{k=1}^{\left| D\right| }\left( \overline{S^{\Diamond }} \right) _{{\mathcal {D}}ominan\left( {\mathcal {T}}_{k}^{*},C\right) ^{+}}^{\beta \vee l\left( o\right) }.$ Similarly, the optimistic lower approximation and optimistic upper approximation of $S^{\Diamond }$ with respect to ${\mathcal {D}}ominan\left( {\mathcal {T}}_{k}^{*},C\right) ^{-}$ are defined by

$$\begin{aligned} \sum \limits _{k=1}^{\left| D\right| }\left( \underline{S^{\Diamond }} \right) _{{\mathcal {D}}ominan\left( {\mathcal {T}}_{k}^{*},C\right) ^{-}}^{\beta \vee l\left( o\right) }=\bigvee \limits _{k=1}^{\left| D\right| }\left\{ \begin{array}{c} b\in A\mid \left( \frac{\left| \left[ b\right] _{{\mathcal {D}}ominan \left( {\mathcal {T}}_{k}^{*},C\right) }^{-}\cap S^{\Diamond }\right| }{\left| \left[ b\right] _{{\mathcal {D}}ominan\left( {\mathcal {T}} _{k}^{*},C\right) }^{-}\right| }\right) >\beta \\ \vee \left( \left| \left[ b\right] _{{\mathcal {D}}ominan\left( {\mathcal {T}}_{k}^{*},C\right) }^{-}\right| -\left| \left[ b\right] _{{\mathcal {D}}ominan\left( {\mathcal {T}}_{k}^{*},C\right) }^{-}\cap S^{\Diamond }\right| \right) <l \end{array}\right\} ; \end{aligned}$$

and

$$\begin{aligned} \sum \limits _{k=1}^{\left| D\right| }\left( \overline{S^{\Diamond }} \right) _{{\mathcal {D}}ominan\left( {\mathcal {T}}_{k}^{*},C\right) ^{-}}^{\beta \vee l\left( o\right) }=\bigwedge \limits _{k=1}^{\left| D\right| }\left\{ \begin{array}{c} b\in A\mid \left( \frac{\left| \left[ b\right] _{{\mathcal {D}}ominan \left( {\mathcal {T}}_{k}^{*},C\right) }^{-}\cap S^{\Diamond }\right| }{\left| \left[ b\right] _{{\mathcal {D}}ominan\left( {\mathcal {T}} _{k}^{*},C\right) }^{-}\right| }\right) \ge 1-\beta \\ \vee \left( \left| \left[ b\right] _{{\mathcal {D}}ominan\left( {\mathcal {T}}_{k}^{*},C\right) }^{-}\cap S^{\Diamond }\right| \right) \ge l \end{array}\right\} . \end{aligned}$$

The pair $\left( \sum \nolimits _{k=1}^{\left| D\right| }\left( \underline{S^{\Diamond }}\right) _{{\mathcal {D}}ominan\left( {\mathcal {T}}_{k}^{*},C\right) ^{-}}^{\beta \vee l\left( o\right) },\sum \nolimits _{k=1}^{\left| D\right| }\left( \overline{S^{\Diamond }} \right) _{{\mathcal {D}}ominan\left( {\mathcal {T}}_{k}^{*},C\right) ^{-}}^{\beta \vee l\left( o\right) }\right)$ is called the logical disjunction soft dominated optimistic multigranulation rough set if $\sum \nolimits _{k=1}^{\left| D\right| }\left( \underline{S^{\Diamond }} \right) _{{\mathcal {D}}ominan\left( {\mathcal {T}}_{k}^{*},C\right) ^{-}}^{\beta \vee l\left( o\right) }\ne \sum \nolimits _{k=1}^{\left| D\right| }\left( \overline{S^{\Diamond }}\right) _{{\mathcal {D}}ominan \left( {\mathcal {T}}_{k}^{*},C\right) ^{-}}^{\beta \vee l\left( o\right) }.$ Using the lower approximation $\sum \nolimits _{k=1}^{\left| D\right| }\left( \underline{X^{\Diamond }}\right) _{{\mathcal {D}}ominan \left( {\mathcal {T}}_{k}^{*},C\right) ^{+}}^{\beta \vee l\left( o\right) }$ and upper approximation $\sum \nolimits _{k=1}^{\left| D\right| }\left( \overline{S^{\Diamond }}\right) _{{\mathcal {D}}ominan \left( {\mathcal {T}}_{k}^{*},C\right) ^{+}}^{\beta \vee l\left( o\right) },$ the logical disjunction soft dominating optimistic multigranulation boundary region of $S^{\Diamond }$ is

$$\begin{aligned} \sum \limits _{k=1}^{\left| D\right| }\left( S^{\Diamond }\right) _{ {\mathcal {D}}ominan\left( {\mathcal {T}}_{k}^{*},C\right) ^{+}}^{\beta \vee l\left( o\right) }=\sum \limits _{k=1}^{\left| D\right| }\left( \overline{S^{\Diamond }}\right) _{{\mathcal {D}}ominan \left( {\mathcal {T}}_{k}^{*},C\right) ^{+}}^{\beta \vee l\left( o\right) }-\sum \limits _{k=1}^{\left| D\right| }\left( \underline{ S^{\Diamond }}\right) _{{\mathcal {D}}ominan\left( {\mathcal {T}} _{k}^{*},C\right) ^{+}}^{\beta \vee l\left( o\right) }. \end{aligned}$$

Similarly, the lower approximation $\sum \nolimits _{k=1}^{\left| D\right| }\left( \underline{X}\right) _{{\mathcal {D}}ominan\left( {\mathcal {T}}_{k}^{*},C\right) ^{-}}^{\beta \vee l\left( o\right) }$ and upper approximation $\sum \nolimits _{k=1}^{\left| D\right| }\left( \overline{S^{\Diamond }}\right) _{{\mathcal {D}}ominan\left( {\mathcal {T}}_{k}^{*},C\right) ^{-}}^{\beta \vee l\left( o\right) },$ the logical disjunction soft dominated optimistic multigranulation boundary region of $S^{\Diamond }$ is $$\begin{aligned} \sum \limits _{k=1}^{\left| D\right| }\left( S^{\Diamond }\right) _{ {\mathcal {D}}ominan\left( {\mathcal {T}}_{k}^{*},C\right) ^{-}}^{\beta \vee l\left( o\right) }=\sum \limits _{k=1}^{\left| D\right| }\left( \overline{S^{\Diamond }}\right) _{{\mathcal {D}}ominan \left( {\mathcal {T}}_{k}^{*},C\right) ^{-}}^{\beta \vee l\left( o\right) }-\sum \limits _{k=1}^{\left| D\right| }\left( \underline{ S^{\Diamond }}\right) _{{\mathcal {D}}ominan\left( {\mathcal {T}} _{k}^{*},C\right) ^{-}}^{\beta \vee l\left( o\right) }. \end{aligned}$$

#### Theorem 5

Let ${\mathcal {I}}{\mathcal {S}}^{*}=\left( A,C,D,E\right)$ be an incomplete information system. If $\beta \in \left( 0.5,1\right]$ and $l\in {\mathbb {N}} \cup \{0\},$ then for any $S^{\Diamond }\subseteq A$ the following hold:

1. $$\sum \nolimits _{k=1}^{\left| D\right| }\left( \underline{S^{\Diamond }}\right) _{{\mathcal {D}}ominan\left( {\mathcal {T}}_{k}^{*},C\right) ^{+}}^{\beta \vee l\left( o\right) }=\sum \nolimits _{k=1}^{\left| D\right| }\left( \underline{S^{\Diamond }}\right) _{{\mathcal {D}}ominan\left( {\mathcal {T}}_{k}^{*},C\right) ^{+}}^{\beta \left( o\right) }\cup \sum \nolimits _{k=1}^{\left| D\right| }\left( \underline{S^{\Diamond }}\right) _{{\mathcal {D}}ominan \left( {\mathcal {T}}_{k}^{*},C\right) ^{+}}^{l\left( o\right) };$$
2. $$\sum \nolimits _{k=1}^{\left| D\right| }\left( \overline{S^{\Diamond }}\right) _{{\mathcal {D}}ominan\left( {\mathcal {T}}_{k}^{*},C\right) ^{+}}^{\beta \vee l\left( o\right) }=\sum \nolimits _{k=1}^{\left| D\right| }\left( \overline{S^{\Diamond }} \right) _{{\mathcal {D}}ominan\left( {\mathcal {T}}_{k}^{*},C\right) ^{+}}^{\beta \left( o\right) }\cup \sum \nolimits _{k=1}^{\left| D\right| }\left( \overline{S^{\Diamond }}\right) _{{\mathcal {D}}ominan \left( {\mathcal {T}}_{k}^{*},C\right) ^{+}}^{l\left( o\right) };$$
3. $$\sum \nolimits _{k=1}^{\left| D\right| }\left( \underline{A}\right) _{{\mathcal {D}}ominan\left( {\mathcal {T}} _{k}^{*},C\right) ^{+}}^{\beta \vee l\left( o\right) }=\sum \nolimits _{k=1}^{\left| D\right| }\left( \overline{A}\right) _{ {\mathcal {D}}ominan\left( {\mathcal {T}}_{k}^{*},C\right) ^{+}}^{\beta \vee l\left( o\right) }=A;$$
4. $$\sum \nolimits _{k=1}^{\left| D\right| }\left( \underline{\emptyset }\right) _{{\mathcal {D}}ominan\left( {\mathcal {T}} _{k}^{*},C\right) ^{+}}^{\beta \vee l\left( o\right) }=\sum \nolimits _{k=1}^{\left| D\right| }\left( \overline{\emptyset } \right) _{{\mathcal {D}}ominan\left( {\mathcal {T}}_{k}^{*},C\right) ^{+}}^{\beta \vee l\left( o\right) }=\emptyset ;$$
5. $$\sum \nolimits _{k=1}^{\left| D\right| }\left( \underline{S^{\Diamond }}\right) _{{\mathcal {D}}ominan\left( {\mathcal {T}}_{k}^{*},C\right) ^{+}}^{\beta \vee l_{1}\left( o\right) }\subseteq \sum \nolimits _{k=1}^{\left| D\right| }\left( \underline{S^{\Diamond }} \right) _{{\mathcal {D}}ominan\left( {\mathcal {T}}_{k}^{*},C\right) ^{+}}^{\beta \vee l_{2}\left( o\right) },$$ $$\sum \nolimits _{k=1}^{\left| D\right| }\left( \overline{S^{\Diamond }}\right) _{{\mathcal {D}}ominan \left( {\mathcal {T}}_{k}^{*},C\right) ^{+}}^{\beta \vee l_{2}\left( o\right) }\subseteq \sum \nolimits _{k=1}^{\left| D\right| }\left( \overline{S^{\Diamond }}\right) _{{\mathcal {D}}ominan\left( {\mathcal {T}}_{k}^{*},C\right) ^{+}}^{\beta \vee l_{1}\left( o\right) },$$ if $$ l_{1}\le l_{2}\in {\mathbb {N}} ;$$
6. $$\sum \nolimits _{k=1}^{\left| D\right| }\left( \underline{S^{\Diamond }}\right) _{{\mathcal {D}}ominan\left( {\mathcal {T}}_{k}^{*},C\right) ^{+}}^{\beta _{2}\vee l\left( o\right) }\subseteq \sum \nolimits _{k=1}^{\left| D\right| }\left( \underline{S^{\Diamond }} \right) _{{\mathcal {D}}ominan\left( {\mathcal {T}}_{k}^{*},C\right) ^{+}}^{\beta _{1}\vee l\left( o\right) },$$ $$\sum \nolimits _{k=1}^{\left| D\right| }\left( \overline{S^{\Diamond }}\right) _{{\mathcal {D}}ominan \left( {\mathcal {T}}_{k}^{*},C\right) ^{+}}^{\beta _{1}\vee l\left( o\right) }\subseteq \sum \nolimits _{k=1}^{\left| D\right| }\left( \overline{S^{\Diamond }}\right) _{{\mathcal {D}}ominan\left( {\mathcal {T}}_{k}^{*},C\right) ^{+}}^{\beta _{2}\vee l\left( o\right) },$$ if $$\beta _{1}\le \beta _{2}\in \left( 0.5,1\right] ;$$
7. $$\sum \nolimits _{k=1}^{\left| D\right| }\left( \underline{S^{\Diamond }}\right) _{{\mathcal {D}}ominan\left( {\mathcal {T}}_{k}^{*},C\right) ^{+}}^{\beta \vee l\left( o\right) }\cup \sum \nolimits _{k=1}^{\left| D\right| }\left( \underline{S^{\Diamond }} \right) _{{\mathcal {D}}ominan\left( {\mathcal {T}}_{k}^{*},C\right) ^{+}}^{\beta \vee l\left( o\right) }\subseteq \sum \nolimits _{k=1}^{\left| D\right| }\left( \underline{S^{\Diamond }\cup P^{\Diamond }}\right) _{ {\mathcal {D}}ominan\left( {\mathcal {T}}_{k}^{*},C\right) ^{+}}^{\beta \vee l\left( o\right) },$$ for all $$S^{\Diamond },$$ $$P^{\Diamond }\subseteq A;$$
8. $$\sum \nolimits _{k=1}^{\left| D\right| }\left( \overline{S^{\Diamond }}\right) _{{\mathcal {D}}ominan\left( {\mathcal {T}}_{k}^{*},C\right) ^{+}}^{\beta \vee l\left( o\right) }\cup \sum \nolimits _{k=1}^{\left| D\right| }\left( \overline{P^{\Diamond }} \right) _{{\mathcal {D}}ominan\left( {\mathcal {T}}_{k}^{*},C\right) ^{+}}^{\beta \vee l\left( o\right) }\subseteq \sum \nolimits _{k=1}^{\left| D\right| }\left( \overline{S^{\Diamond }\cup P^{\Diamond }}\right) _{ {\mathcal {D}}ominan\left( {\mathcal {T}}_{k}^{*},C\right) ^{+}}^{\beta \vee l\left( o\right) },$$ for all $$S^{\Diamond },$$ $$P^{\Diamond }\subseteq A;$$
9. $$\sum \nolimits _{k=1}^{\left| D\right| }\left( \underline{S^{\Diamond }}\right) _{{\mathcal {D}}ominan\left( {\mathcal {T}}_{k}^{*},C\right) ^{+}}^{\beta \vee l\left( o\right) }\cap \sum \nolimits _{k=1}^{\left| D\right| }\left( \underline{P^{\Diamond }} \right) _{{\mathcal {D}}ominan\left( {\mathcal {T}}_{k}^{*},C\right) ^{+}}^{\beta \vee l\left( o\right) }\supseteq \sum \nolimits _{k=1}^{\left| D\right| }\left( \underline{S^{\Diamond }\cap P^{\Diamond }}\right) _{ {\mathcal {D}}ominan\left( {\mathcal {T}}_{k}^{*},C\right) ^{+}}^{\beta \vee l\left( o\right) },$$ for all $$S^{\Diamond },$$ $$P^{\Diamond }\subseteq A;$$
10. $$\sum \nolimits _{k=1}^{\left| D\right| }\left( \overline{S^{\Diamond }}\right) _{{\mathcal {D}}ominan\left( {\mathcal {T}}_{k}^{*},C\right) ^{+}}^{\beta \vee l\left( o\right) }\cap \sum \nolimits _{k=1}^{\left| D\right| }\left( \overline{P^{\Diamond }} \right) _{{\mathcal {D}}ominan\left( {\mathcal {T}}_{k}^{*},C\right) ^{+}}^{\beta \vee l\left( o\right) }\supseteq \sum \nolimits _{k=1}^{\left| D\right| }\left( \overline{S^{\Diamond }\cap P^{\Diamond }}\right) _{ {\mathcal {D}}ominan\left( {\mathcal {T}}_{k}^{*},C\right) ^{+}}^{\beta \vee l\left( o\right) },$$ for all $$S^{\Diamond },$$ $$P^{\Diamond }\subseteq A.$$

#### Proof

$$\left( 1\right) $$ Let $$b\in \sum \nolimits _{k=1}^{\left| D\right| }\left( \underline{S^{\Diamond }}\right) _{{\mathcal {D}}ominan\left( {\mathcal {T}}_{k}^{*},C\right) ^{+}}^{\beta \vee l\left( o\right) }.$$ Then

$b\in \bigvee \limits _{k=1}^{\left| D\right| }\left\{ \left( \frac{ \left| \left[ b\right] _{{\mathcal {D}}ominan\left( {\mathcal {T}} _{k}^{*},C\right) }^{+}\cap S^{\Diamond }\right| }{\left| \left[ b\right] _{{\mathcal {D}}ominan\left( {\mathcal {T}}_{k}^{*},C\right) }^{+}\right| }\right) >\beta \vee \left( \left| \left[ b\right] _{ {\mathcal {D}}ominan\left( {\mathcal {T}}_{k}^{*},C\right) }^{+}\right| -\left| \left[ b\right] _{{\mathcal {D}}ominan\left( {\mathcal {T}}_{k}^{*},C\right) }^{+}\cap S^{\Diamond }\right| \right) <l\right\} .$ This implies that $b\in \left\{ \bigvee \limits _{k=1}^{\left| D\right| }\left( \frac{ \left| \left[ b\right] _{{\mathcal {D}}ominan\left( {\mathcal {T}} _{k}^{*},C\right) }^{+}\cap S^{\Diamond }\right| }{\left| \left[ b\right] _{{\mathcal {D}}ominan\left( {\mathcal {T}}_{k}^{*},C\right) }^{+}\right| }\right) >\beta \vee \bigvee \limits _{k=1}^{\left| D\right| }\left( \left| \left[ b\right] _{{\mathcal {D}}ominan\left( {\mathcal {T}}_{k}^{*},C\right) }^{+}\right| -\left| \left[ b\right] _{{\mathcal {D}}ominan\left( {\mathcal {T}}_{k}^{*},C\right) }^{+}\cap S^{\Diamond }\right| \right) <l\right\} ,$ which further implies that $b\in \bigvee \limits _{k=1}^{\left| D\right| }\left( \frac{\left| \left[ b\right] _{{\mathcal {D}}ominan\left( {\mathcal {T}}_{k}^{*},C\right) }^{+}\cap S^{\Diamond }\right| }{\left| \left[ b\right] _{{\mathcal {D}}ominan\left( {\mathcal {T}}_{k}^{*},C\right) }^{+}\right| }\right) >\beta$ or $b\in \bigvee \limits _{k=1}^{\left| D\right| }\left( \left| \left[ b\right] _{{\mathcal {D}}ominan\left( {\mathcal {T}}_{k}^{*},C\right) }^{+}\right| -\left| \left[ b\right] _{{\mathcal {D}}ominan\left( {\mathcal {T}}_{k}^{*},C\right) }^{+}\cap S^{\Diamond }\right| \right) <l.$ Hence $b\in \sum \nolimits _{k=1}^{\left| D\right| }\left( \underline{S^{\Diamond }}\right) _{{\mathcal {D}}ominan\left( {\mathcal {T}}_{k}^{*},C\right) ^{+}}^{\beta \left( o\right) }$ or $b\in \sum \nolimits _{k=1}^{\left| D\right| }\left( \underline{S^{\Diamond }} \right) _{{\mathcal {D}}ominan\left( {\mathcal {T}}_{k}^{*},C\right) ^{+}}^{l\left( o\right) }.$ Therefore we have $b\in \sum \nolimits _{k=1}^{\left| D\right| }\left( \underline{S^{\Diamond }} \right) _{{\mathcal {D}}ominan\left( {\mathcal {T}}_{k}^{*},C\right) ^{+}}^{\beta \left( o\right) }\cup \sum \nolimits _{k=1}^{\left| D\right| }\left( \underline{S^{\Diamond }}\right) _{{\mathcal {D}}ominan \left( {\mathcal {T}}_{k}^{*},C\right) ^{+}}^{l\left( o\right) }.$ On the other hand, for any $b\in \sum \nolimits _{k=1}^{\left| D\right| }\left( \underline{S^{\Diamond }}\right) _{{\mathcal {D}}ominan\left( {\mathcal {T}}_{k}^{*},C\right) ^{+}}^{\beta \left( o\right) }\cup \sum \nolimits _{k=1}^{\left| D\right| }\left( \underline{S^{\Diamond }} \right) _{{\mathcal {D}}ominan\left( {\mathcal {T}}_{k}^{*},C\right) ^{+}}^{l\left( o\right) },$ we can get that $b\in \sum \nolimits _{k=1}^{\left| D\right| }\left( \underline{S^{\Diamond }} \right) _{{\mathcal {D}}ominan\left( {\mathcal {T}}_{k}^{*},C\right) ^{+}}^{\beta \left( o\right) }$ or $b\in \sum \nolimits _{k=1}^{\left| D\right| }\left( \underline{S^{\Diamond }}\right) _{{\mathcal {D}}ominan \left( {\mathcal {T}}_{k}^{*},C\right) ^{+}}^{l\left( o\right) },$ that is, $b\in \bigvee \limits _{k=1}^{\left| D\right| }\left( \frac{ \left| \left[ b\right] _{{\mathcal {D}}ominan\left( {\mathcal {T}} _{k}^{*},C\right) }^{+}\cap S^{\Diamond }\right| }{\left| \left[ b\right] _{{\mathcal {D}}ominan\left( {\mathcal {T}}_{k}^{*},C\right) }^{+}\right| }\right) >\beta$ or $b\in \bigvee \limits _{k=1}^{\left| D\right| }\left( \left| \left[ b\right] _{{\mathcal {D}}ominan\left( {\mathcal {T}}_{k}^{*},C\right) }^{+}\right| -\left| \left[ b\right] _{{\mathcal {D}}ominan\left( {\mathcal {T}}_{k}^{*},C\right) }^{+}\cap S^{\Diamond }\right| \right) <l.$ This implies that

$$b\in \bigvee \limits _{k=1}^{\left| D\right| }\left\{ \left( \frac{ \left| \left[ b\right] _{{\mathcal {D}}ominan\left( {\mathcal {T}} _{k}^{*},C\right) }^{+}\cap S^{\Diamond }\right| }{\left| \left[ b\right] _{{\mathcal {D}}ominan\left( {\mathcal {T}}_{k}^{*},C\right) }^{+}\right| }\right) >\beta \vee \left( \left| \left[ b\right] _{ {\mathcal {D}}ominan\left( {\mathcal {T}}_{k}^{*},C\right) }^{+}\right| -\left| \left[ b\right] _{{\mathcal {D}}ominan\left( {\mathcal {T}}_{k}^{*},C\right) }^{+}\cap S^{\Diamond }\right| \right) <l\right\} .$$ Hence $$b\in \sum \nolimits _{k=1}^{\left| D\right| }\left( \underline{S^{\Diamond }}\right) _{{\mathcal {D}}ominan\left( {\mathcal {T}}_{k}^{*},C\right) ^{+}}^{\beta \vee l\left( o\right) }.$$ Consequently, $$\sum \nolimits _{k=1}^{\left| D\right| }\left( \underline{ S^{\Diamond }}\right) _{{\mathcal {D}}ominan\left( {\mathcal {T}} _{k}^{*},C\right) ^{+}}^{\beta \vee l\left( o\right) }=\sum \nolimits _{k=1}^{\left| D\right| }\left( \underline{S^{\Diamond }}\right) _{{\mathcal {D}}ominan\left( {\mathcal {T}}_{k}^{*},C\right) ^{+}}^{\beta \left( o\right) }\cup \sum \nolimits _{k=1}^{\left| D\right| }\left( \underline{S^{\Diamond }}\right) _{{\mathcal {D}}ominan \left( {\mathcal {T}}_{k}^{*},C\right) ^{+}}^{l\left( o\right) }.$$

$\left( 2\right)$ The proof process is similar to the proof of $\left( 1\right) .$

$\left( 3\right)$ Since $\sum \nolimits _{k=1}^{\left| D\right| }\left( \underline{A}\right) _{{\mathcal {D}}ominan\left( {\mathcal {T}} _{k}^{*},C\right) ^{+}}^{\beta \left( o\right) }=\sum \nolimits _{k=1}^{\left| D\right| }\left( \overline{A}\right) _{ {\mathcal {D}}ominan\left( {\mathcal {T}}_{k}^{*},C\right) ^{+}}^{\beta \left( o\right) }=A$ and $\sum \nolimits _{k=1}^{\left| D\right| }\left( \underline{A}\right) _{{\mathcal {D}}ominan\left( {\mathcal {T}}_{k}^{*},C\right) ^{+}}^{l\left( o\right) }=\sum \nolimits _{k=1}^{\left| D\right| }\left( \overline{A}\right) _{ {\mathcal {D}}ominan\left( {\mathcal {T}}_{k}^{*},C\right) ^{+}}^{l\left( o\right) }=A,$ using $\left( 1\right)$, we can achieve that $\sum \nolimits _{k=1}^{\left| D\right| }\left( \underline{A}\right) _{ {\mathcal {D}}ominan\left( {\mathcal {T}}_{k}^{*},C\right) ^{+}}^{\beta \vee l\left( o\right) }=\sum \nolimits _{k=1}^{\left| D\right| }\left( \overline{A}\right) _{{\mathcal {D}}ominan\left( {\mathcal {T}}_{k}^{*},C\right) ^{+}}^{\beta \vee l\left( o\right) }=A.$

$\left( 4\right)$ The proof process is similar to the proof of $\left( 3\right) .$

$\left( 5\right)$ Using $\left( 1\right) ,$ we only prove $\sum \nolimits _{k=1}^{\left| D\right| }\left( \underline{S^{\Diamond }} \right) _{{\mathcal {D}}ominan\left( {\mathcal {T}}_{k}^{*},C\right) ^{+}}^{l_{1}\left( o\right) }\subseteq \sum \nolimits _{k=1}^{\left| D\right| }\left( \underline{S^{\Diamond }}\right) _{{\mathcal {D}}ominan \left( {\mathcal {T}}_{k}^{*},C\right) ^{+}}^{l_{2}\left( o\right) }.$ For this let $b\in \sum \nolimits _{k=1}^{\left| D\right| }\left( \underline{S^{\Diamond }}\right) _{{\mathcal {D}}ominan\left( {\mathcal {T}}_{k}^{*},C\right) ^{+}}^{l_{1}\left( o\right) }.$ Then $\bigvee \limits _{k=1}^{\left| D\right| }\left( \left| \left[ b \right] _{{\mathcal {D}}ominan\left( {\mathcal {T}}_{k}^{*},C\right) }^{+}\right| -\left| \left[ b\right] _{{\mathcal {D}}ominan\left( {\mathcal {T}}_{k}^{*},C\right) }^{+}\cap S^{\Diamond }\right| \right) <l_{1}.$ But $l_{1}\le l_{2},$ so $\bigvee \limits _{k=1}^{\left| D\right| }\left( \left| \left[ b\right] _{{\mathcal {D}}ominan\left( {\mathcal {T}}_{k}^{*},C\right) }^{+}\right| -\left| \left[ b\right] _{{\mathcal {D}}ominan\left( {\mathcal {T}}_{k}^{*},C\right) }^{+}\cap S^{\Diamond }\right| \right) <l_{2},$ that is, $b\in \sum \nolimits _{k=1}^{\left| D\right| }\left( \underline{S^{\Diamond }} \right) _{{\mathcal {D}}ominan\left( {\mathcal {T}}_{k}^{*},C\right) ^{+}}^{l_{2}\left( o\right) }.$ Hence $\sum \nolimits _{k=1}^{\left| D\right| }\left( \underline{S^{\Diamond }}\right) _{{\mathcal {D}}ominan \left( {\mathcal {T}}_{k}^{*},C\right) ^{+}}^{l_{1}\left( o\right) }\subseteq \sum \nolimits _{k=1}^{\left| D\right| }\left( \underline{ S^{\Diamond }}\right) _{{\mathcal {D}}ominan\left( {\mathcal {T}} _{k}^{*},C\right) ^{+}}^{l_{2}\left( o\right) }.$ Consequently $\sum \nolimits _{k=1}^{\left| D\right| }\left( \underline{S^{\Diamond }} \right) _{{\mathcal {D}}ominan\left( {\mathcal {T}}_{k}^{*},C\right) ^{+}}^{\beta \vee l_{1}\left( o\right) }\subseteq \sum \nolimits _{k=1}^{\left| D\right| }\left( \underline{S^{\Diamond }} \right) _{{\mathcal {D}}ominan\left( {\mathcal {T}}_{k}^{*},C\right) ^{+}}^{\beta \vee l_{2}\left( o\right) }.$ Similarly, we can get $\sum \nolimits _{k=1}^{\left| D\right| }\left( \overline{S^{\Diamond }} \right) _{{\mathcal {D}}ominan\left( {\mathcal {T}}_{k}^{*},C\right) ^{+}}^{\beta \vee l_{2}\left( o\right) }\subseteq \sum \nolimits _{k=1}^{\left| D\right| }\left( \overline{S^{\Diamond }} \right) _{{\mathcal {D}}ominan\left( {\mathcal {T}}_{k}^{*},C\right) ^{+}}^{\beta \vee l_{1}\left( o\right) }.$

$\left( 6\right)$ The proof process is similar to the proof of $\left( 5\right) .$

$$\left( 7\right) $$ Let $$b\in \sum \nolimits _{k=1}^{\left| D\right| }\left( \underline{S^{\Diamond }}\right) _{{\mathcal {D}}ominan\left( {\mathcal {T}}_{k}^{*},C\right) ^{+}}^{\beta \vee l\left( o\right) }.$$ Then

$$b\in \bigvee \limits _{k=1}^{\left| D\right| } \left\{ \begin{array}{c} \left( \frac{\left| \left[ b\right] _{{\mathcal {D}}ominan\left( {\mathcal {T}}_{k}^{*},C\right) }^{+}\cap S^{\Diamond }\right| }{ \left| \left[ b\right] _{{\mathcal {D}}ominan\left( {\mathcal {T}} _{k}^{*},C\right) }^{+}\right| }\right) >\beta \\ \vee \left( \left| \left[ b\right] _{{\mathcal {D}}ominan\left( {\mathcal {T}}_{k}^{*},C\right) }^{+}\right| -\left| \left[ b\right] _{{\mathcal {D}}ominan\left( {\mathcal {T}}_{k}^{*},C\right) }^{+}\cap S^{\Diamond }\right| \right) <l \end{array}\right\} .$$ This implies that

$b\in \bigvee \limits _{k=1}^{\left| D\right| }\left( \frac{\left| \left[ b\right] _{{\mathcal {D}}ominan\left( {\mathcal {T}}_{k}^{*},C\right) }^{+}\cap S^{\Diamond }\right| }{\left| \left[ b\right] _{ {\mathcal {D}}ominan\left( {\mathcal {T}}_{k}^{*},C\right) }^{+}\right| }\right) >\beta$ or $b\in \bigvee \limits _{k=1}^{\left| D\right| }\left( \left| \left[ b\right] _{{\mathcal {D}}ominan\left( {\mathcal {T}}_{k}^{*},C\right) }^{+}\right| -\left| \left[ b\right] _{{\mathcal {D}}ominan\left( {\mathcal {T}}_{k}^{*},C\right) }^{+}\cap S^{\Diamond }\right| \right) <l.$ We can get ${\mathcal {D}}om inan\left( {\mathcal {T}}_{i},C\right) ,$ ${\mathcal {D}}ominan\left( {\mathcal {T}}_{j},C\right)$ such that $\left( \frac{\left| \left[ b\right] _{{\mathcal {D}}ominan\left( {\mathcal {T}}_{i},C\right) }^{+}\cap S^{\Diamond }\right| }{\left| \left[ b\right] _{{\mathcal {D}} ominan\left( {\mathcal {T}}_{i},C\right) }^{+}\right| }\right) >\beta$ or $\left( \left| \left[ b\right] _{{\mathcal {D}}ominan \left( {\mathcal {T}}_{j},C\right) }^{+}\right| -\left| \left[ b \right] _{{\mathcal {D}}ominan\left( {\mathcal {T}}_{j},C\right) }^{+}\cap S^{\Diamond }\right| \right) <l,$ for any $P^{\Diamond }\subseteq A,$ we know that $\left( \frac{\left| \left[ b\right] _{ {\mathcal {D}}ominan\left( {\mathcal {T}}_{i},C\right) }^{+}\cap \left( S^{\Diamond }\cup P^{\Diamond }\right) \right| }{\left| \left[ b \right] _{{\mathcal {D}}ominan\left( {\mathcal {T}}_{i},C\right) }^{+}\right| }\right) \ge \left( \frac{\left| \left[ b\right] _{ {\mathcal {D}}ominan\left( {\mathcal {T}}_{i},C\right) }^{+}\cap S^{\Diamond }\right| }{\left| \left[ b\right] _{{\mathcal {D}}ominan \left( {\mathcal {T}}_{i},C\right) }^{+}\right| }\right) >\beta$ or $\left( \left| \left[ b\right] _{{\mathcal {D}}ominan\left( {\mathcal {T}}_{j},C\right) }^{+}\right| -\left| \left[ b\right] _{ {\mathcal {D}}ominan\left( {\mathcal {T}}_{j},C\right) }^{+}\cap \left( S^{\Diamond }\cup P^{\Diamond }\right) \right| \right)$ $\le \left( \left| \left[ b\right] _{{\mathcal {D}}ominan\left( {\mathcal {T}}_{j},C\right) }^{+}\right| -\left| \left[ b\right] _{ {\mathcal {D}}ominan\left( {\mathcal {T}}_{j},C\right) }^{+}\cap S^{\Diamond }\right| \right) <l,$ that is $b\in \sum \nolimits _{k=1}^{\left| D\right| }\left( \underline{S^{\Diamond }\cup P}^{\Diamond }\right) _{{\mathcal {D}}ominan\left( {\mathcal {T}} _{k}^{*},C\right) ^{+}}^{\beta \vee l\left( o\right) }.$ Hence $\sum \nolimits _{k=1}^{\left| D\right| }\left( \underline{S^{\Diamond }} \right) _{{\mathcal {D}}ominan\left( {\mathcal {T}}_{k}^{*},C\right) ^{+}}^{\beta \vee l\left( o\right) }\subseteq \sum \nolimits _{k=1}^{\left| D\right| }\left( \underline{S^{\Diamond }\cup P^{\Diamond }}\right) _{ {\mathcal {D}}ominan\left( {\mathcal {T}}_{k}^{*},C\right) ^{+}}^{\beta \vee l\left( o\right) }.$ Similarly we can get $\sum \nolimits _{k=1}^{\left| D\right| }\left( \underline{P^{\Diamond }} \right) _{{\mathcal {D}}ominan\left( {\mathcal {T}}_{k}^{*},C\right) ^{+}}^{\beta \vee l\left( o\right) }\subseteq \sum \nolimits _{k=1}^{\left| D\right| }\left( \underline{S^{\Diamond }\cup P^{\Diamond }}\right) _{ {\mathcal {D}}ominan\left( {\mathcal {T}}_{k}^{*},C\right) ^{+}}^{\beta \vee l\left( o\right) }.$ Therefore $\sum \nolimits _{k=1}^{\left| D\right| }\left( \underline{S^{\Diamond }}\right) _{{\mathcal {D}}om inan\left( {\mathcal {T}}_{k}^{*},C\right) ^{+}}^{\beta \vee l\left( o\right) }\cup \sum \nolimits _{k=1}^{\left| D\right| }\left( \underline{P^{\Diamond }}\right) _{{\mathcal {D}}ominan\left( {\mathcal {T}}_{k}^{*},C\right) ^{+}}^{\beta \vee l\left( o\right) }\subseteq \sum \nolimits _{k=1}^{\left| D\right| }\left( \underline{S^{\Diamond }\cup P^{\Diamond }}\right) _{{\mathcal {D}}ominan\left( {\mathcal {T}} _{k}^{*},C\right) ^{+}}^{\beta \vee l\left( o\right) }.$

$\left( 8\right)$ The proof process is similar to the proof of $\left( 7\right) .$

$$\left( 9\right) $$ Let $$b\in \sum \nolimits _{k=1}^{\left| D\right| }\left( \underline{S^{\Diamond }\cap P^{\Diamond }}\right) _{{\mathcal {D}}om inan\left( {\mathcal {T}}_{k}^{*},C\right) ^{+}}^{\beta \vee l\left( o\right) }.$$ Then

$$b\in \bigvee \limits _{k=1}^{\left| D\right| } \left\{ \begin{array}{c} \left( \frac{\left| \left[ b\right] _{{\mathcal {D}}ominan\left( {\mathcal {T}}_{k}^{*},C\right) }^{+}\cap \left( S^{\Diamond }\cap P^{\Diamond }\right) \right| }{\left| \left[ b\right] _{{\mathcal {D}}om inan\left( {\mathcal {T}}_{k}^{*},C\right) }^{+}\right| } \right) >\beta \vee \\ \left( \left| \left[ b\right] _{{\mathcal {D}}ominan\left( {\mathcal {T}}_{k}^{*},C\right) }^{+}\right| -\left| \left[ b\right] _{{\mathcal {D}}ominan\left( {\mathcal {T}}_{k}^{*},C\right) }^{+}\cap \left( S^{\Diamond }\cap P^{\Diamond }\right) \right| \right) <l\end{array} \right\} .$$ This implies that $b\in \bigvee \limits _{k=1}^{\left| D\right| }\left( \frac{\left| \left[ b\right] _{{\mathcal {D}}ominan \left( {\mathcal {T}}_{k}^{*},C\right) }^{+}\cap \left( S^{\Diamond }\cap P\right) \right| }{\left| \left[ b\right] _{{\mathcal {D}}om inan\left( {\mathcal {T}}_{k}^{*},C\right) }^{+}\right| } \right) >\beta$ or $b\in \bigvee \limits _{k=1}^{\left| D\right| }\left( \left| \left[ b\right] _{{\mathcal {D}}ominan\left( {\mathcal {T}}_{k}^{*},C\right) }^{+}\right| -\left| \left[ b\right] _{{\mathcal {D}}ominan\left( {\mathcal {T}}_{k}^{*},C\right) }^{+}\cap \left( S^{\Diamond }\cap P^{\Diamond }\right) \right| \right) <l.$ We can get ${\mathcal {D}}om inan\left( {\mathcal {T}}_{i}^{*},C\right) ,$ ${\mathcal {D}}om inan\left( {\mathcal {T}}_{j}^{*},C\right)$ such that $\left( \frac{\left| \left[ b\right] _{{\mathcal {D}}ominan\left( {\mathcal {T}}_{i}^{*},C\right) }^{+}\cap \left( S^{\Diamond }\cap P^{\Diamond }\right) \right| }{\left| \left[ b\right] _{{\mathcal {D}}ominan\left( {\mathcal {T}}_{i}^{*},C\right) }^{+}\right| }\right) >\beta$ or $\left( \left| \left[ b\right] _{{\mathcal {D}}ominan\left( {\mathcal {T}}_{j}^{*},C\right) }^{+}\right| -\left| \left[ b\right] _{{\mathcal {D}}ominan\left( {\mathcal {T}}_{j}^{*},C\right) }^{+}\cap \left( S^{\Diamond }\cap P^{\Diamond }\right) \right| \right) <l,$ for any $P^{\Diamond }\subseteq A,$ we know that $\left( \frac{ \left| \left[ b\right] _{{\mathcal {D}}ominan\left( {\mathcal {T}} _{i},C\right) }^{+}\cap S^{\Diamond }\right| }{\left| \left[ b\right] _{{\mathcal {D}}ominan\left( {\mathcal {T}}_{i},C\right) }^{+}\right| }\right) \ge \left( \frac{\left| \left[ b\right] _{{\mathcal {D}}ominan \left( {\mathcal {T}}_{i},C\right) }^{+}\cap \left( S^{\Diamond }\cap P^{\Diamond }\right) \right| }{\left| \left[ b\right] _{{\mathcal {D}}om inan\left( {\mathcal {T}}_{i},C\right) }^{+}\right| }\right) >\beta$ or $\left( \left| \left[ b\right] _{{\mathcal {D}}ominan\left( {\mathcal {T}}_{j},C\right) }^{+}\right| -\left| \left[ b\right] _{ {\mathcal {D}}ominan\left( {\mathcal {T}}_{j},C\right) }^{+}\cap S^{\Diamond }\right| \right) \le$ $\left( \left| \left[ b\right] _{{\mathcal {D}}ominan\left( {\mathcal {T}}_{j},C\right) }^{+}\right| -\left| \left[ b\right] _{ {\mathcal {D}}ominan\left( {\mathcal {T}}_{j},C\right) }^{+}\cap \left( S^{\Diamond }\cap P^{\Diamond }\right) \right| \right) <l,$ that is $b\in \sum \nolimits _{k=1}^{\left| D\right| }\left( \underline{ S^{\Diamond }}\right) _{{\mathcal {D}}ominan\left( {\mathcal {T}} _{k}^{*},C\right) ^{+}}^{\beta \vee l\left( o\right) }.$ Hence $\sum \nolimits _{k=1}^{\left| D\right| }\left( \underline{S^{\Diamond }\cap P^{\Diamond }}\right) _{{\mathcal {D}}ominan\left( {\mathcal {T}} _{k}^{*},C\right) ^{+}}^{\beta \vee l\left( o\right) }\subseteq \sum \nolimits _{k=1}^{\left| D\right| }\left( \underline{S^{\Diamond }} \right) _{{\mathcal {D}}ominan\left( {\mathcal {T}}_{k}^{*},C\right) ^{+}}^{\beta \vee l\left( o\right) }.$ Similarly we can get $\sum \nolimits _{k=1}^{\left| D\right| }\left( \underline{S^{\Diamond }\cap P^{\Diamond }}\right) _{{\mathcal {D}}ominan\left( {\mathcal {T}} _{k}^{*},C\right) ^{+}}^{\beta \vee l\left( o\right) }\subseteq \sum \nolimits _{k=1}^{\left| D\right| }\left( \underline{S^{\Diamond }} \right) _{{\mathcal {D}}ominan\left( {\mathcal {T}}_{k}^{*},C\right) ^{+}}^{\beta \vee l\left( o\right) }.$ Therefore $\sum \nolimits _{k=1}^{\left| D\right| }\left( \underline{S^{\Diamond }}\right) _{{\mathcal {D}}om inan\left( {\mathcal {T}}_{k}^{*},C\right) ^{+}}^{\beta \vee l\left( o\right) }\cap \sum \nolimits _{k=1}^{\left| D\right| }\left( \underline{P^{\Diamond }}\right) _{{\mathcal {D}}ominan\left( {\mathcal {T}}_{k}^{*},C\right) ^{+}}^{\beta \vee l\left( o\right) }\supseteq \sum \nolimits _{k=1}^{\left| D\right| }\left( \underline{S^{\Diamond }\cap P^{\Diamond }}\right) _{{\mathcal {D}}ominan\left( {\mathcal {T}} _{k}^{*},C\right) ^{+}}^{\beta \vee l\left( o\right) }.$

$\left( 10\right)$ The proof process is similar to the proof of $\left( 9\right) .$ $\square$

#### Definition 6

Let ${\mathcal {I}}{\mathcal {S}}^{*}=\left( A,C,D,E\right)$ be an incomplete information system. For any $S^{\Diamond }\subseteq A,$ the approximate precisions $\sum \nolimits _{k=1}^{\left| D\right| }\left( S^{\Diamond }\right) _{{\mathcal {D}}ominan\left( {\mathcal {T}}_{k}^{*},C\right) ^{+}}^{\rho \left( \beta \vee l\right) \left( o\right) }$ and $\sum \nolimits _{k=1}^{\left| D\right| }\left( S^{\Diamond }\right) _{ {\mathcal {D}}ominan\left( {\mathcal {T}}_{k}^{*},C\right) ^{-}}^{\rho \left( \beta \vee l\right) \left( o\right) }$ of $S^{\Diamond }$ about ${\mathcal {D}}ominan\left( {\mathcal {T}}_{k}^{*},C\right) ^{+}$ and ${\mathcal {D}}ominan\left( {\mathcal {T}}_{k}^{*},C\right) ^{-}$ are defined by

$$\begin{aligned} \sum \limits _{k=1}^{\left| D\right| }\left( S^{\Diamond }\right) _{ {\mathcal {D}}ominan\left( {\mathcal {T}}_{k}^{*},C\right) ^{+}}^{\rho \left( \beta \vee l\right) \left( o\right) }=\frac{\left| \sum \limits _{k=1}^{\left| D\right| }\left( \underline{S^{\Diamond }} \right) _{{\mathcal {D}}ominan\left( {\mathcal {T}}_{k}^{*},C\right) ^{+}}^{\beta \vee l\left( o\right) }\right| }{\left| \sum \limits _{k=1}^{\left| D\right| }\left( \overline{S^{\Diamond }} \right) _{{\mathcal {D}}ominan\left( {\mathcal {T}}_{k}^{*},C\right) ^{+}}^{\beta \vee l\left( o\right) }\right| }; \end{aligned}$$

and

$$\begin{aligned} \sum \limits _{k=1}^{\left| D\right| }\left( S^{\Diamond }\right) _{{\mathcal {D}}ominan\left( {\mathcal {T}}_{k}^{*},C\right) ^{-}}^{\rho \left( \beta \vee l\right) \left( o\right) }=\frac{\left| \sum \limits _{k=1}^{\left| D\right| }\left( \underline{S^{\Diamond }} \right) _{{\mathcal {D}}ominan\left( {\mathcal {T}}_{k}^{*},C\right) ^{-}}^{\beta \vee l\left( o\right) }\right| }{\left| \sum \limits _{k=1}^{\left| D\right| }\left( \overline{S^{\Diamond }} \right) _{{\mathcal {D}}ominan\left( {\mathcal {T}}_{k}^{*},C\right) ^{-}}^{\beta \vee l\left( o\right) }\right| }; \end{aligned}$$

where $S^{\Diamond }\ne \emptyset$ and $\left| \cdot \right|$ denotes the cardinality of a set.

Let $\sum \nolimits _{k=1}^{\left| D\right| }\left( S^{\Diamond }\right) _{{\mathcal {D}}ominan\left( {\mathcal {T}}_{k}^{*},C\right) ^{+}}^{\mu \left( \beta \vee l\right) \left( o\right) }=1-\sum \nolimits _{k=1}^{\left| D\right| }\left( S^{\Diamond }\right) _{{\mathcal {D}}ominan\left( {\mathcal {T}}_{k}^{*},C\right) ^{+}}^{\rho \left( \beta \vee l\right) \left( o\right) }$ and $\sum \nolimits _{k=1}^{\left| D\right| }\left( S^{\Diamond }\right) _{ {\mathcal {D}}ominan\left( {\mathcal {T}}_{k}^{*},C\right) ^{-}}^{\mu \left( \beta \vee l\right) \left( o\right) }=1-\sum \nolimits _{k=1}^{\left| D\right| }\left( S^{\Diamond }\right) _{{\mathcal {D}}ominan\left( {\mathcal {T}}_{k}^{*},C\right) ^{-}}^{\rho \left( \beta \vee l\right) \left( o\right) },$ then $\sum \nolimits _{k=1}^{\left| D\right| }\left( S^{\Diamond }\right) _{{\mathcal {D}}ominan\left( {\mathcal {T}} _{k}^{*},C\right) ^{+}}^{\mu \left( \beta \vee l\right) \left( o\right) }$ and $\sum \nolimits _{k=1}^{\left| D\right| }\left( S^{\Diamond }\right) _{{\mathcal {D}}ominan\left( {\mathcal {T}}_{k}^{*},C\right) ^{-}}^{\mu \left( \beta \vee l\right) \left( o\right) }$ are called the rough degrees of $S^{\Diamond }$ about ${\mathcal {D}}ominan\left( {\mathcal {T}}_{k}^{*},C\right) ^{+}$ and ${\mathcal {D}}ominan\left( {\mathcal {T}}_{k}^{*},C\right) ^{-}$ respectively.

By definition $0\le \sum \nolimits _{k=1}^{\left| D\right| }\left( S^{\Diamond }\right) _{{\mathcal {D}}ominan\left( {\mathcal {T}} _{k}^{*},C\right) ^{+}}^{\mu \left( \beta \vee l\right) \left( o\right) }\le 1$ and $0\le \sum \nolimits _{k=1}^{\left| D\right| }\left( S^{\Diamond }\right) _{{\mathcal {D}}ominan\left( {\mathcal {T}} _{k}^{*},C\right) ^{-}}^{\mu \left( \beta \vee l\right) \left( o\right) }\le 1.$

#### Definition 7

Let ${\mathcal {I}}{\mathcal {S}}^{*}=\left( A,C,D,E\right)$ be an incomplete information system. For any $S^{\Diamond }\subseteq A,$ the * approximate quality* $\sum \nolimits _{k=1}^{\left| D\right| }\left( S^{\Diamond }\right) _{{\mathcal {D}}ominan\left( {\mathcal {T}} _{k}^{*},C\right) ^{+}}^{\gamma \left( \beta \vee l\right) \left( o\right) }$ and $\sum \nolimits _{k=1}^{\left| D\right| }\left( S^{\Diamond }\right) _{{\mathcal {D}}ominan\left( {\mathcal {T}} _{k}^{*},C\right) ^{-}}^{\gamma \left( \beta \vee l\right) \left( o\right) }$ of $S^{\Diamond }$ about ${\mathcal {D}}ominan\left( {\mathcal {T}}_{k}^{*},C\right) ^{+}$ and ${\mathcal {D}}ominan\left( {\mathcal {T}}_{k}^{*},C\right) ^{-}$ are defined by:

$$\begin{aligned} \sum \limits _{k=1}^{\left| D\right| }\left( S^{\Diamond }\right) _{ {\mathcal {D}}ominan\left( {\mathcal {T}}_{k}^{*},C\right) ^{+}}^{\gamma \left( \beta \vee l\right) \left( o\right) }=\frac{\left| \sum \limits _{k=1}^{\left| D\right| }\left( \underline{S^{\Diamond }} \right) _{{\mathcal {D}}ominan\left( {\mathcal {T}}_{k}^{*},C\right) ^{+}}^{\beta \vee l\left( o\right) }\right| }{\left| A\right| } \end{aligned}$$

and

$$\begin{aligned} \sum \limits _{k=1}^{\left| D\right| }\left( S^{\Diamond }\right) _{ {\mathcal {D}}ominan\left( {\mathcal {T}}_{k}^{*},C\right) ^{-}}^{\gamma \left( \beta \vee l\right) \left( o\right) }=\frac{\left| \sum \limits _{k=1}^{\left| D\right| }\left( \underline{S^{\Diamond }} \right) _{{\mathcal {D}}ominan\left( {\mathcal {T}}_{k}^{*},C\right) ^{-}}^{\beta \vee l\left( o\right) }\right| }{\left| A\right| }. \end{aligned}$$

Clearly $$0\le \sum \nolimits _{k=1}^{\left| D\right| }\left( S^{\Diamond }\right) _{{\mathcal {D}}ominan\left( {\mathcal {T}} _{k}^{*},C\right) ^{+}}^{\gamma \left( \beta \vee l\right) \left( o\right) }\le 1$$ and $$0\le \sum \nolimits _{k=1}^{\left| D\right| }\left( S^{\Diamond }\right) _{{\mathcal {D}}ominan\left( {\mathcal {T}} _{k}^{*},C\right) ^{-}}^{\gamma \left( \beta \vee l\right) \left( o\right) }\le 1.$$

We now obtain three way decision rules using logical disjunction soft dominating optimistic multigranulation rough sets:

$\left( \mathrm{i}\right)$ If there exists $k\in \left\{ 1,2,3,...,\left| D\right| \right\}$ such that $\left( \frac{ \left| \left[ b\right] _{{\mathcal {D}}ominan\left( {\mathcal {T}} _{k}^{*},C\right) }^{+}\cap S^{\Diamond }\right| }{\left| \left[ b\right] _{{\mathcal {D}}ominan\left( {\mathcal {T}}_{k}^{*},C\right) }^{+}\right| }\right) >\beta \vee \left( \left| \left[ b\right] _{ {\mathcal {D}}ominan\left( {\mathcal {T}}_{k}^{*},C\right) }^{+}\right| -\left| \left[ b\right] _{{\mathcal {D}}ominan\left( {\mathcal {T}}_{k}^{*},C\right) }^{+}\cap S^{\Diamond }\right| \right) <l,$ decide $Pos\left( S^{\Diamond }\right) _{{\mathcal {D}}ominan \left( {\mathcal {T}}_{k}^{*},C\right) ^{+}}^{\beta \vee l\left( o\right) };$

$\left( \mathrm{ii}\right)$ If there exists for all $k\in \left\{ 1,2,3,...,\left| D\right| \right\}$ such that $\left( \frac{ \left| \left[ b\right] _{{\mathcal {D}}ominan\left( {\mathcal {T}} _{k}^{*},C\right) }^{+}\cap S^{\Diamond }\right| }{\left| \left[ b\right] _{{\mathcal {D}}ominan\left( {\mathcal {T}}_{k}^{*},C\right) }^{+}\right| }\right)<1-\beta \vee \left( \left| \left[ b\right] _{ {\mathcal {D}}ominan\left( {\mathcal {T}}_{k}^{*},C\right) }^{+}\right| -\left| \left[ b\right] _{{\mathcal {D}}ominan\left( {\mathcal {T}}_{k}^{*},C\right) }^{+}\cap S^{\Diamond }\right| \right) <l,$ decide $Neg\left( S^{\Diamond }\right) _{{\mathcal {D}}ominan \left( {\mathcal {T}}_{k}^{*},C\right) ^{+}}^{\beta \vee l\left( o\right) };$

$$\left( \mathrm{iii}\right) $$ Otherwise, decide $$\sum \nolimits _{k=1}^{\left| D\right| }\left( S^{\Diamond }\right) _{{\mathcal {D}}ominan\left( {\mathcal {T}}_{k}^{*},C\right) ^{+}}^{\beta \vee l\left( o\right) }.$$

Similarly, we obtained three way decision rules using logical disjunction soft dominated optimistic multigranulation rough sets:

$\left( \mathrm{i}\right)$ If there exists $k\in \left\{ 1,2,3,...,\left| D\right| \right\}$ such that $\left( \frac{ \left| \left[ b\right] _{{\mathcal {D}}ominan\left( {\mathcal {T}} _{k}^{*},C\right) }^{-}\cap S^{\Diamond }\right| }{\left| \left[ b\right] _{{\mathcal {D}}ominan\left( {\mathcal {T}}_{k}^{*},C\right) }^{-}\right| }\right) >\beta \vee \left( \left| \left[ b\right] _{ {\mathcal {D}}ominan\left( {\mathcal {T}}_{k}^{*},C\right) }^{-}\right| -\left| \left[ b\right] _{{\mathcal {D}}ominan\left( {\mathcal {T}}_{k}^{*},C\right) }^{-}\cap S^{\Diamond }\right| \right) <l,$ decide $Pos\left( S^{\Diamond }\right) _{{\mathcal {D}}ominan \left( {\mathcal {T}}_{k}^{*},C\right) ^{-}}^{\beta \vee l\left( o\right) };$

$\left( \mathrm{ii}\right)$ If there exists for all $k\in \left\{ 1,2,3,...,\left| D\right| \right\}$ such that $\left( \frac{ \left| \left[ b\right] _{{\mathcal {D}}ominan\left( {\mathcal {T}} _{k}^{*},C\right) }^{-}\cap S^{\Diamond }\right| }{\left| \left[ b\right] _{{\mathcal {D}}ominan\left( {\mathcal {T}}_{k}^{*},C\right) }^{-}\right| }\right)<1-\beta \vee \left( \left| \left[ b\right] _{ {\mathcal {D}}ominan\left( {\mathcal {T}}_{k}^{*},C\right) }^{-}\right| -\left| \left[ b\right] _{{\mathcal {D}}ominan\left( {\mathcal {T}}_{k}^{*},C\right) }^{-}\cap S^{\Diamond }\right| \right) <l,$ decide $Neg\left( S^{\Diamond }\right) _{{\mathcal {D}}ominan \left( {\mathcal {T}}_{k}^{*},C\right) ^{-}}^{\beta \vee l\left( o\right) };$

$$\left( \mathrm{iii}\right) $$ Otherwise, decide $$\sum \nolimits _{k=1}^{\left| D\right| }\left( S^{\Diamond }\right) _{{\mathcal {D}}ominan\left( {\mathcal {T}}_{k}^{*},C\right) ^{-}}^{\beta \vee l\left( o\right) }.$$

### Proposed Algorithm-$\mathrm{I}$ for conflict analysis model

**Input:** Given information system ${\mathcal {I}}{\mathcal {S}}^{*}=\left( A,C,D,E\right) ;$

**Step** 1: Construct $\left( {\mathcal {T}}_{k}^{*},C\right)$ corresponding to the $D_{k}$ for all $k=1,2,...,\left| D\right|$;

**Step** 2: Construct ${\mathcal {D}}ominan\left( {\mathcal {T}} _{k}^{*},C\right) ,$ for all $k:=1,2,...,\left| D\right|$;

**Step** 3: Construct $\sum \nolimits _{k=1}^{\left| D\right| }\left( \underline{S_{k}^{\Diamond }}\right) _{{\mathcal {D}}ominan \left( {\mathcal {T}}_{k}^{*},C\right) ^{+}}^{\beta \vee l\left( o\right) }$ and $\sum \nolimits _{k=1}^{\left| D\right| }\left( \underline{ S_{k}^{\Diamond }}\right) _{{\mathcal {D}}ominan\left( {\mathcal {T}} _{k}^{*},C\right) ^{-}}^{\beta \vee l\left( o\right) }$, where $S^{\Diamond }=\left\{ b\in A:g(b,D_{k})\ne 0\right\} ;$

**Step** 4: If $\sum \nolimits _{k=1}^{\left| D\right| }\left( \underline{S_{k}^{\Diamond }}\right) _{{\mathcal {D}}ominan\left( {\mathcal {T}}_{k}^{*},C\right) ^{+}}^{\beta \vee l\left( o\right) }=\sum \nolimits _{k=1}^{\left| D\right| }\left( \underline{ S_{k}^{\Diamond }}\right) _{{\mathcal {D}}ominan\left( {\mathcal {T}} _{k}^{*},C\right) ^{-}}^{\beta \vee l\left( o\right) }$ then $\delta _{k}=\sum \nolimits _{k=1}^{\left| D\right| }\left( \underline{ S_{k}^{\Diamond }}\right) _{{\mathcal {D}}ominan\left( {\mathcal {T}} _{k}^{*},C\right) ^{+}}^{\beta \vee l\left( o\right) }$ or $\delta _{k}=\sum \nolimits _{k=1}^{\left| D\right| }\left( \underline{ X_{k}^{\Diamond }}\right) _{{\mathcal {D}}ominan\left( {\mathcal {T}} _{k}^{*},C\right) ^{-}}^{\beta \vee l\left( o\right) }$ otherwise $\delta _{k}=\sum \nolimits _{k=1}^{\left| D\right| }\left( \underline{ S_{k}^{\Diamond }}\right) _{{\mathcal {D}}ominan\left( {\mathcal {T}} _{k}^{*},C\right) ^{+}}^{\beta \vee l\left( o\right) }-\sum \nolimits _{k=1}^{\left| D\right| }\left( \underline{ S_{k}^{\Diamond }}\right) _{{\mathcal {D}}ominan\left( {\mathcal {T}} _{k}^{*},C\right) ^{-}}^{\beta \vee l\left( o\right) };$

**Step** 5: $$\delta =\bigcap \nolimits _{k=1}^{\left| D\right| }\delta _{k};$$

**Step** 6: If $$\delta \ne \emptyset ;$$

         go to **output.**

         otherwise,

         put $\beta ^{\prime }$ in **Step** 3, where $\beta ^{\prime }\le \beta ,$

or

         put $l^{\prime }$ in **Step** 3, where $l\le l^{\prime };$

**Output:** Feasible alternative(s) for feasible consensus strategy with decision precision $\beta$ and.

#### Example 2

(Continued from Example 1) Let ${\mathcal {I}}{\mathcal {S}}^{*}=\left( A,C,D,E\right)$ be an incomplete information system. Applying Algorithm-$\mathrm{I},$ construct soft preference relations for the symptoms corresponding to doctors, $\left( {\mathcal {F}}_{1}^{*},C\right)$ corresponding to $D_{1},$ similarly $\left( {\mathcal {F}}_{2}^{*},C\right)$ corresponding to $D_{2}$ and so on from the given incomplete information system ${\mathcal {I}}{\mathcal {S}}^{*}=\left( A,C,D,E\right) .$ Applying Definition 1 and Definition 2 to construct soft dominance relations.

$$\begin{aligned} {\mathcal {D}}ominan\left( {\mathcal {T}}_{2}^{*},C\right) =\left\{ \begin{array}{c} \left( b_{1},b_{1}\right) ,\ \left( b_{2},b_{2}\right) ,\ \left( b_{3},b_{3}\right) ,\ \left( b_{4},b_{4}\right) ,\ \left( b_{5},b_{5}\right) ,\ \left( b_{6},b_{6}\right) ,\left( b_{7},b_{7}\right) , \\ \left( b_{9},b_{9}\right) ,\ \left( b_{10},b_{10}\right) ,\ \left( b_{11},b_{11}\right) ,\ \left( b_{12},b_{12}\right) ,\ \left( b_{1},b_{2}\right) ,\ \left( b_{2},b_{1}\right) ,\ \left( b_{2},b_{4}\right) , \\ \ \left( b_{2},b_{5}\right) ,\ \left( b_{2},b_{8}\right) , \ \left( b_{2},b_{10}\right) ,\ \left( b_{2},b_{11}\right) , \left( b_{2},b_{12}\right) ,\ \left( b_{3},b_{1}\right) ,\ \left( b_{5},b_{1}\right) , \\ \left( b_{5},b_{4}\right) ,\ \left( b_{6},b_{1}\right) ,\ \left( b_{7},b_{1}\right) ,\left( b_{7},b_{3}\right) ,\ \left( b_{7},b_{4}\right) ,\ \left( b_{7},b_{5}\right) ,\ \left( b_{7},b_{6}\right) , \\ \left( b_{5},b_{4}\right) ,\ \left( b_{8},b_{1}\right) ,\ \left( b_{8},b_{3}\right) ,\ \left( b_{8},b_{4}\right) ,\ \left( b_{8},b_{5}\right) ,\ \left( b_{8},b_{10}\right) ,\ \left( b_{8},b_{11}\right) , \\ \ \left( b_{9},b_{1}\right) ,\left( b_{9},b_{4}\right) ,\ \left( b_{9},b_{5}\right) ,\ \left( b_{9},b_{8}\right) ,\ \left( b_{9},b_{11}\right) ,\left( b_{9},b_{12}\right) ,\left( b_{8},b_{8}\right) , \\ \left( b_{10},b_{4}\right) ,\ \left( b_{10},b_{8}\right) ,\ \left( b_{12},b_{11}\right) \end{array}\right\} . \end{aligned}$$

The soft dominance classes from ${\mathcal {D}}ominan\left( {\mathcal {T}} _{2}^{*},C\right)$ are given in the following Table 3.

$${\mathcal {D}}ominan\left( {\mathcal {T}}_{3}^{*},C\right) =\left\{ \begin{array}{c} \left( b_{1},b_{1}\right) ,\ \left( b_{2},b_{2}\right) ,\ \left( b_{3},b_{3}\right) ,\ \left( b_{4},b_{4}\right) ,\ \left( b_{5},b_{5}\right) ,\ \left( b_{6},b_{6}\right) , \\ \left( b_{7},b_{7}\right) ,\left( b_{9},b_{9}\right) ,\ \left( b_{10},b_{10}\right) ,\ \left( b_{11},b_{11}\right) ,\ \left( b_{12},b_{12}\right) ,\ \left( b_{1},b_{2}\right) ,\ \\ \left( b_{2},b_{4}\right) ,\ \left( b_{2},b_{5}\right) ,\ \left( b_{2},b_{8}\right) ,\ \left( b_{5},b_{4}\right) ,\ \left( b_{6},b_{1}\right) ,\left( b_{6},b_{2}\right) ,\ \\ \left( b_{6},b_{3}\right) ,\ \left( b_{6},b_{4}\right) ,\ \left( b_{6},b_{5}\right) ,\left( b_{7},b_{1}\right) ,\ \left( b_{7},b_{2}\right) ,\ \left( b_{7},b_{3}\right) ,\ \\ \left( b_{7},b_{4}\right) ,\ \left( b_{7},b_{5}\right) ,\ \left( b_{7},b_{6}\right) ,\ \left( b_{8},b_{3}\right) ,\ \left( b_{8},b_{4}\right) ,\ \left( b_{8},b_{5}\right) ,\ \\ \left( b_{9},b_{1}\right) ,\ \left( b_{9},b_{2}\right) ,\ \left( b_{9},b_{4}\right) ,\ \left( b_{9},b_{5}\right) ,\ \left( b_{9},b_{8}\right) ,\ \left( b_{9},b_{10}\right) , \\ \left( b_{9},b_{12}\right) ,\left( b_{10},b_{4}\right) ,\ \left( b_{10},b_{8}\right) ,\ \left( b_{11},b_{10}\right) ,\ \left( b_{12},b_{10}\right) ,\ \left( b_{8},b_{8}\right) \end{array}\right\} .$$

The soft dominance classes from ${\mathcal {D}}ominan\left( {\mathcal {T}} _{3}^{*},C\right)$ are given in the following Table 4.

$${\mathcal {D}}ominan\left( {\mathcal {T}}_{4}^{*},C\right) =\left\{ \begin{array}{c} \left( b_{1},b_{1}\right) ,\ \left( b_{2},b_{2}\right) ,\ \left( b_{3},b_{3}\right) ,\ \left( b_{4},b_{4}\right) ,\ \left( b_{5},b_{5}\right) ,\ \left( b_{6},b_{6}\right) , \\ \left( b_{7},b_{7}\right) ,\left( b_{9},b_{9}\right) ,\ \left( b_{10},b_{10}\right) ,\ \left( b_{11},b_{11}\right) ,\ \left( b_{12},b_{12}\right) ,\left( b_{1},b_{2}\right) ,\ \\ \left( b_{2},b_{1}\right) ,\ \left( b_{3},b_{1}\right) ,\ \left( b_{5},b_{1}\right) ,\ \left( b_{6},b_{1}\right) ,\left( b_{7},b_{1}\right) ,\left( b_{7},b_{2}\right) , \\ \left( b_{7},b_{4}\right) ,\ \left( b_{7},b_{5}\right) ,\ \left( b_{7},b_{6}\right) ,\ \left( b_{8},b_{1}\right) ,\left( b_{8},b_{4}\right) ,\left( b_{8},b_{5}\right) , \\ \left( b_{8},b_{11}\right) ,\ \left( b_{9},b_{1}\right) ,\left( b_{9},b_{2}\right) ,\ \left( b_{9},b_{4}\right) ,\ \left( b_{9},b_{5}\right) ,\ \left( b_{9},b_{8}\right) , \\ \left( b_{9},b_{10}\right) ,\ \left( b_{10},b_{4}\right) ,\ \left( b_{10},b_{8}\right) ,\ \left( b_{12},b_{11}\right) \end{array}\right\} .$$

The soft dominance classes from ${\mathcal {D}}ominan\left( {\mathcal {T}} _{4}^{*},C\right)$ are given in the following Table 5.

Using Tables 6 and 7 to get $\delta =\left\{ b_{7}\right\} .$ The result shows that $b_{7}$ is the optimal decision medicine with decision precision 0.9. Since the action $b_{7}$ is a medicine for solving this conflict analysis problem, on which most of the doctors have agreed in the conflict situation ${\mathcal {I}}{\mathcal {S}}^{*}.$ As it is well known that, the optimal strategy which satisfy all doctors in a conflict situation, that is, the feasible consensus strategy usually doesn’t exist because of unlike conveniences and judgements. Therefore a suboptimal feasible consensus strategy which satisfy the doctors as much as possible is a reasonable target. Therefore we answer the second and third questions proposed by Deja in^64^ for the classical Pawlak conflict analysis decision making model.

**Table 3:** Dominating classes $\left[ b_{i}\right] _{{\mathcal {D}}ominan \left( {\mathcal {T}}_{2}^{*},C\right) }^{+}$ and Dominated classes $\left[ b_{i}\right] _{{\mathcal {D}}ominan\left( {\mathcal {T}}_{2}^{*}, C\right) }^{-}$.

$b_{i}$	$\left[ b_{i}\right] _{{\mathcal {D}}om inan\left( {\mathcal {T}}_{2}^{*},C\right) }^{+}$	$\left[ b_{i}\right] _{{\mathcal {D}}ominan\left( {\mathcal {T}}_{2}^{*},C\right) }^{-}$
$b_{1}$	$\left\{ b_{1},b_{2},b_{3},b_{5},b_{6},b_{7},b_{8},b_{9}\right\}$	$\left\{ b_{1}\right\}$
$b_{2}$	$\left\{ b_{1},b_{2}\right\}$	$\left\{ b_{1},b_{2},b_{4},b_{5},b_{8},b_{10},b_{11},b_{12}\right\}$
$b_{3}$	$\left\{ b_{3},b_{7},b_{8}\right\}$	$\left\{ b_{1},b_{3}\right\}$
$b_{4}$	$\left\{ b_{2},b_{4},b_{5},b_{7},b_{8},b_{9},b_{10}\right\}$	$\left\{ b_{4}\right\}$
$b_{5}$	$\left\{ b_{5},b_{7},b_{8},b_{9}\right\}$	$\left\{ b_{1},b_{4},b_{5}\right\}$
$b_{6}$	$\left\{ b_{6},b_{7}\right\}$	$\left\{ b_{1},b_{6}\right\}$
$b_{7}$	$\left\{ b_{7}\right\}$	$\left\{ b_{1},b_{3},b_{4},b_{5,}b_{6},b_{7}\right\}$
$b_{8}$	$\left\{ b_{2},b_{8},b_{9},b_{10}\right\}$	$\left\{ b_{1},b_{3},b_{4},b_{5,}b_{8},b_{10},b_{11}\right\}$
$b_{9}$	$\left\{ b_{9}\right\}$	$\left\{ b_{1},b_{4},b_{5},b_{8},b_{9},b_{11},b_{12}\right\}$
$b_{10}$	$\left\{ b_{2},b_{10}\right\}$	$\left\{ b_{4},b_{8},b_{10}\right\}$
$b_{11}$	$\left\{ b_{2},b_{8},b_{9},b_{11},b_{12}\right\}$	$\left\{ b_{11}\right\}$
$b_{12}$	$\left\{ b_{2},b_{9},b_{12}\right\}$	$\left\{ b_{11},b_{12}\right\}$

**Table 4:** Dominating classes $\left[ b_{i}\right] _{{\mathcal {D}}ominan \left( {\mathcal {T}}_{3}^{*},C\right) }^{+}$ and Dominated classes $\left[ b_{i}\right] _{{\mathcal {D}}ominan \left( {\mathcal {T}}_{3}^{*},C\right) }^{-}$.

$b_{i}$	$\left[ b_{i}\right] _{{\mathcal {D}}om inan\left( {\mathcal {T}}_{3}^{*},C\right) }^{+}$	$\left[ b_{i}\right] _{{\mathcal {D}}ominan \left( {\mathcal {T}}_{3}^{*},C\right) }^{-}$
$b_{1}$	$\left\{ b_{1},b_{6},b_{7},b_{9}\right\}$	$\left\{ b_{1},b_{2}\right\}$
$b_{2}$	$\left\{ b_{1},b_{2},b_{6},b_{7},b_{9}\right\}$	$\left\{ b_{2},b_{4},b_{5},b_{8}\right\}$
$b_{3}$	$\left\{ b_{3},b_{6},b_{7},b_{8}\right\}$	$\left\{ b_{3}\right\}$
$b_{4}$	$\left\{ b_{2},b_{4},b_{5},b_{6},b_{7},b_{8},b_{9},b_{10}\right\}$	$\left\{ b_{4}\right\}$
$b_{5}$	$\left\{ b_{2},b_{5},b_{6},b_{7},b_{8},b_{9}\right\}$	$\left\{ b_{4},b_{5}\right\}$
$b_{6}$	$\left\{ b_{6},b_{7}\right\}$	$\left\{ b_{1},b_{2},b_{3},b_{4},b_{5,}b_{6}\right\}$
$b_{7}$	$\left\{ b_{7}\right\}$	$\left\{ b_{1},b_{2},b_{3},b_{4},b_{5,}b_{6},b_{7}\right\}$
$b_{8}$	$\left\{ b_{2},b_{8},b_{9},b_{10}\right\}$	$\left\{ b_{4},b_{8},b_{10}\right\}$
$b_{9}$	$\left\{ b_{9}\right\}$	$\left\{ b_{1},b_{2},b_{4},b_{5},b_{8},b_{9},b_{10},b_{12}\right\}$
$b_{10}$	$\left\{ b_{9},b_{10},b_{11},b_{12}\right\}$	$\left\{ b_{4},b_{8},b_{10}\right\}$
$b_{11}$	$\left\{ b_{11}\right\}$	$\left\{ b_{10},b_{11}\right\}$
$b_{12}$	$\left\{ b_{9},b_{12}\right\}$	$\left\{ b_{10},b_{12}\right\}$

**Table 5:** Dominating classes $\left[ b_{i}\right] _{{\mathcal {D}}ominan \left( {\mathcal {T}}_{4}^{*},C\right) }^{+}$ and Dominated classes $\left[ b_{i}\right] _{{\mathcal {D}}ominan \left( {\mathcal {T}}_{4}^{*},C\right) }^{-}$.

$b_{i}$	$\left[ b_{i}\right] _{{\mathcal {D}}ominan \left( {\mathcal {T}}_{4}^{*},C\right) }^{+}$	$\left[ b_{i}\right] _{{\mathcal {D}}ominan \left( {\mathcal {T}}_{4}^{*},C\right) }^{-}$
$b_{1}$	$\left\{ b_{1},b_{2},b_{3},b_{5},b_{6},b_{7},b_{8},b_{9}\right\}$	$\left\{ b_{1},b_{2}\right\}$
$b_{2}$	$\left\{ b_{1},b_{2},b_{7}\right\}$	$\left\{ b_{1},b_{2}\right\}$
$b_{3}$	$\left\{ b_{3}\right\}$	$\left\{ b_{1},b_{3}\right\}$
$b_{4}$	$\left\{ b_{4},b_{7},b_{8},b_{9},b_{10}\right\}$	$\left\{ b_{4}\right\}$
$b_{5}$	$\left\{ b_{5},b_{7},b_{8},b_{9}\right\}$	$\left\{ b_{1},b_{5}\right\}$
$b_{6}$	$\left\{ b_{6},b_{7}\right\}$	$\left\{ b_{1},b_{6}\right\}$
$b_{7}$	$\left\{ b_{7}\right\}$	$\left\{ b_{1},b_{2},b_{4},b_{5,}b_{6},b_{7}\right\}$
$b_{8}$	$\left\{ b_{8},b_{9}\right\}$	$\left\{ b_{1},b_{4},b_{5},b_{8},b_{11}\right\}$
$b_{9}$	$\left\{ b_{9}\right\}$	$\left\{ b_{1},b_{2},b_{4},b_{5,}b_{8},b_{9},b_{10}\right\}$
$b_{10}$	$\left\{ b_{9},b_{10}\right\}$	$\left\{ b_{4},b_{8},b_{10}\right\}$
$b_{11}$	$\left\{ b_{8},b_{11},b_{12}\right\}$	$\left\{ b_{11}\right\}$
$b_{12}$	$\left\{ b_{12}\right\}$	$\left\{ b_{11},b_{12}\right\}$

**Table 6:** The lower approximations table with $\beta =0.90, l=1$.

$S^{\Diamond }$	$\sum \limits _{k=1}^{\left| D\right| } \left( \underline{S_{k}}\right) _{{\mathcal {D}}ominan \left( {\mathcal {T}}_{k}^{*},C\right) ^{+}}^{\beta \vee l \left( o\right) }$	$\sum \nolimits _{k=1}^{\left| D\right| } \left( \underline{S_{k}}\right) _{{\mathcal {D}}ominan \left( {\mathcal {T}}_{k}^{*},C\right) ^{-}}^{\beta \vee l\left( o\right) }$
$S_{1}^{\Diamond }$	$\left\{ b_{1},b_{2},b_{3},b_{4},b_{6},b_{7},b_{9},b_{10},b_{12}\right\}$	$\left\{ b_{1},b_{2},b_{3},b_{4},b_{6},b_{12}\right\}$
$S_{2}^{\Diamond }$	$\left\{ b_{1},b_{2},b_{3},b_{4},b_{5},b_{7},b_{8},b_{9},b_{10},b_{11},b_{12}\right\}$	$\left\{ b_{1},b_{2},b_{3},b_{4},b_{5},b_{8},b_{9},b_{10},b_{11},b_{12}\right\}$
$S_{3}^{\Diamond }$	$\left\{ b_{1},b_{6},b_{7},b_{9},b_{11},b_{12}\right\}$	$\left\{ b_{1},b_{4},b_{6},b_{11},b_{12}\right\}$
$S_{4}^{\Diamond }$	$\left\{ b_{1},b_{2},b_{3},b_{4},b_{5},b_{7},b_{8},b_{9},b_{10},b_{12}\right\}$	$\left\{ b_{1},b_{2},b_{3},b_{4},b_{5},b_{8},b_{9},b_{10},b_{12}\right\}$

**Table 7:** Decision table with $\beta =0.90, l=1$.

$\delta _{k}$	$\sum \limits _{k=1}^{\left| D\right| }\left( \underline{S_{k}}\right) _{{\mathcal {D}}ominan\left( {\mathcal {T}}_{k}^{*},C\right) ^{+}}^{\beta \vee l\left( o\right) }-\sum \limits _{k=1}^{\left| D\right| }\left( \underline{ S_{k}}\right) _{{\mathcal {D}}ominan\left( {\mathcal {T}}_{k}^{*},C\right) ^{-}}^{\beta \vee l\left( o\right) }$
$\delta _{1}$	$\left\{ b_{7},b_{9},b_{10}\right\}$
$\delta _{2}$	$\left\{ b_{7}\right\}$
$\delta _{3}$	$\left\{ b_{7},b_{9}\right\}$
$\delta _{4}$	$\left\{ b_{7}\right\}$

### Logical conjunction soft dominating/dominated optimistic multigranulation rough sets

#### Definition 8

Let ${\mathcal {I}}{\mathcal {S}}^{*}=\left( A,C,D,E\right)$ be an incomplete information system. If $\beta \in \left( 0.5,1\right]$ and $l\in {\mathbb {N}} \cup \left\{ 0\right\} ,$ then for any $S^{\Diamond }\subseteq A,$ the optimistic lower approximation and optimistic upper approximation of $S^{\Diamond }$ with respect to ${\mathcal {D}}ominan\left( {\mathcal {T}} _{k}^{*}, C\right) ^{+}$ are defined by

$$\begin{aligned} \sum \limits _{k=1}^{\left| D\right| }\left( \underline{S^{\Diamond }} \right) _{{\mathcal {D}}ominan\left( {\mathcal {T}}_{k}^{*},C\right) ^{+}}^{\beta \wedge l\left( o\right) }=\bigvee \limits _{k=1}^{\left| D\right| }\left\{ \begin{array}{c} b\in A\mid \left( \frac{\left| \left[ b\right] _{{\mathcal {D}}ominan \left( {\mathcal {T}}_{k}^{*},C\right) }^{+}\cap S^{\Diamond }\right| }{\left| \left[ b\right] _{{\mathcal {D}}ominan\left( {\mathcal {T}} _{k}^{*},C\right) }^{+}\right| }\right) >\beta \\ \wedge \left( \left| \left[ b\right] _{{\mathcal {D}}ominan\left( {\mathcal {T}}_{k}^{*},C\right) }^{+}\right| -\left| \left[ b\right] _{{\mathcal {D}}ominan\left( {\mathcal {T}}_{k}^{*},C\right) }^{+} \cap S^{\Diamond }\right| \right) <l \end{array}\right\} ; \end{aligned}$$

and

$$\begin{aligned} \sum \limits _{k=1}^{\left| D\right| }\left( \overline{S^{\Diamond }} \right) _{{\mathcal {D}}ominan\left( {\mathcal {T}}_{k}^{*},C\right) ^{+}}^{\beta \wedge l\left( o\right) }=\bigwedge \limits _{k=1}^{\left| D\right| }\left\{ \begin{array}{c} b\in A\mid \left( \frac{\left| \left[ b\right] _{{\mathcal {D}}ominan \left( {\mathcal {T}}_{k}^{*},C\right) }^{+}\cap S^{\Diamond }\right| }{\left| \left[ b\right] _{{\mathcal {D}}ominan\left( {\mathcal {T}} _{k}^{*},C\right) }^{+}\right| }\right) \ge 1-\beta \\ \wedge \left( \left| \left[ b\right] _{{\mathcal {D}}ominan \left( {\mathcal {T}}_{k}^{*},C\right) }^{+}\cap S^{\Diamond } \right| \right) \ge l \end{array}\right\} . \end{aligned}$$

The pair $\left( \sum \nolimits _{k=1}^{\left| D\right| }\left( \underline{S^{\Diamond }}\right) _{{\mathcal {D}}ominan\left( {\mathcal {T}}_{k}^{*},C\right) ^{+}}^{\beta \wedge l\left( o\right) },\sum \nolimits _{k=1}^{\left| D\right| }\left( \overline{S^{\Diamond }} \right) _{{\mathcal {D}}ominan\left( {\mathcal {T}}_{k}^{*},C\right) ^{+}}^{\beta \wedge l\left( o\right) }\right)$ is called the logical conjunction soft dominating optimistic multigranulation rough set if $\sum \nolimits _{k=1}^{\left| D\right| }\left( \underline{S^{\Diamond }} \right) _{{\mathcal {D}}ominan\left( {\mathcal {T}}_{k}^{*},C\right) ^{+}}^{\beta \wedge l\left( o\right) }\ne \sum \nolimits _{k=1}^{\left| D\right| }\left( \overline{S^{\Diamond }}\right) _{{\mathcal {D}}ominan \left( {\mathcal {T}}_{k}^{*},C\right) ^{+}}^{\beta \wedge l\left( o\right) }.$ Similarly the optimistic lower approximation and optimistic upper approximation of $S^{\Diamond }$ with respect to ${\mathcal {D}}ominan\left( {\mathcal {T}}_{k}^{*},C\right) ^{-}$ are defined by

$$\begin{aligned} \sum \limits _{k=1}^{\left| D\right| }\left( \underline{S^{\Diamond }} \right) _{{\mathcal {D}}ominan\left( {\mathcal {T}}_{k}^{*},C\right) ^{-}}^{\beta \wedge l\left( o\right) }=\bigvee \limits _{k=1}^{\left| D\right| }\left\{ \begin{array}{c} b\in A\mid \left( \frac{\left| \left[ b\right] _{{\mathcal {D}}ominan \left( {\mathcal {T}}_{k}^{*},C\right) }^{-}\cap S^{\Diamond }\right| }{\left| \left[ b\right] _{{\mathcal {D}}ominan\left( {\mathcal {T}} _{k}^{*},C\right) }^{-}\right| }\right) >\beta \\ \wedge \left( \left| \left[ b\right] _{{\mathcal {D}}ominan\left( {\mathcal {T}}_{k}^{*},C\right) }^{-}\right| -\left| \left[ b\right] _{{\mathcal {D}}ominan\left( {\mathcal {T}}_{k}^{*},C\right) }^{-}\cap S^{\Diamond }\right| \right) <l \end{array}\right\} ; \end{aligned}$$

and

$$\begin{aligned} \sum \limits _{k=1}^{\left| D\right| }\left( \overline{S^{\Diamond }} \right) _{{\mathcal {D}}ominan\left( {\mathcal {T}}_{k}^{*},C\right) ^{-}}^{\beta \wedge l\left( o\right) }=\bigwedge \limits _{k=1}^{\left| D\right| }\left\{ \begin{array}{c} b\in A\mid \left( \frac{\left| \left[ b\right] _{{\mathcal {D}}ominan \left( {\mathcal {T}}_{k}^{*},C\right) }^{-}\cap S^{\Diamond }\right| }{\left| \left[ b\right] _{{\mathcal {D}}ominan\left( {\mathcal {T}} _{k}^{*},C\right) }^{-}\right| }\right) \ge 1-\beta \\ \wedge \left( \left| \left[ b\right] _{{\mathcal {D}}ominan\left( {\mathcal {T}}_{k}^{*},C\right) }^{-}\cap S^{\Diamond }\right| \right) \ge l \end{array}\right\} . \end{aligned}$$

The pair $\left( \sum \nolimits _{k=1}^{\left| D\right| }\left( \underline{S^{\Diamond }}\right) _{{\mathcal {D}}ominan\left( {\mathcal {T}}_{k}^{*},C\right) ^{-}}^{\beta \wedge l\left( o\right) },\sum \nolimits _{k=1}^{\left| D\right| }\left( \overline{S^{\Diamond }} \right) _{{\mathcal {D}}ominan\left( {\mathcal {T}}_{k}^{*},C\right) ^{-}}^{\beta \wedge l\left( o\right) }\right)$ is called the logical conjunction soft dominated optimistic multigranulation rough set if $\sum \nolimits _{k=1}^{\left| D\right| }\left( \underline{S^{\Diamond }} \right) _{{\mathcal {D}}ominan\left( {\mathcal {T}}_{k}^{*},C\right) ^{-}}^{\beta \wedge l\left( o\right) }\ne \sum \nolimits _{k=1}^{\left| D\right| }\left( \overline{S^{\Diamond }}\right) _{{\mathcal {D}}ominan \left( {\mathcal {T}}_{k}^{*},C\right) ^{-}}^{\beta \wedge l\left( o\right) }.$ Using the lower approximation $\sum \nolimits _{k=1}^{\left| D\right| }\left( \underline{X^{\Diamond }}\right) _{{\mathcal {D}}ominan \left( {\mathcal {T}}_{k}^{*},C\right) ^{+}}^{\beta \wedge l\left( o\right) }$ and upper approximation $\sum \nolimits _{k=1}^{\left| D\right| }\left( \overline{S^{\Diamond }}\right) _{{\mathcal {D}}ominan \left( {\mathcal {T}}_{k}^{*},C\right) ^{+}}^{\beta \wedge l\left( o\right) },$ the logical conjunction soft dominating optimistic multigranulation boundary region of $S^{\Diamond }$ is

$$\begin{aligned} \sum \limits _{k=1}^{\left| D\right| }\left( S^{\Diamond }\right) _{ {\mathcal {D}}ominan\left( {\mathcal {T}}_{k}^{*},C\right) ^{+}}^{\beta \wedge l\left( o\right) }=\sum \limits _{k=1}^{\left| D\right| }\left( \overline{S^{\Diamond }}\right) _{{\mathcal {D}}ominan \left( {\mathcal {T}}_{k}^{*},C\right) ^{+}}^{\beta \wedge l\left( o\right) }-\sum \limits _{k=1}^{\left| D\right| }\left( \underline{ S^{\Diamond }}\right) _{{\mathcal {D}}ominan\left( {\mathcal {T}} _{k}^{*},C\right) ^{+}}^{\beta \wedge l\left( o\right) }. \end{aligned}$$

Similarly the lower approximation $\sum \nolimits _{k=1}^{\left| D\right| }\left( \underline{X}\right) _{{\mathcal {D}}ominan\left( {\mathcal {T}}_{k}^{*},C\right) ^{-}}^{\beta \wedge l\left( o\right) }$ and upper approximation $\sum \nolimits _{k=1}^{\left| D\right| }\left( \overline{S^{\Diamond }}\right) _{{\mathcal {D}}ominan\left( {\mathcal {T}}_{k}^{*},C\right) ^{-}}^{\beta \wedge l\left( o\right) },$ the logical conjunction soft dominated optimistic multigranulation boundary region of $S^{\Diamond }$ is

$$\begin{aligned} \sum \limits _{k=1}^{\left| D\right| }\left( S^{\Diamond }\right) _{ {\mathcal {D}}ominan\left( {\mathcal {T}}_{k}^{*},C\right) ^{-}}^{\beta \wedge l\left( o\right) }=\sum \limits _{k=1}^{\left| D\right| }\left( \overline{S^{\Diamond }}\right) _{{\mathcal {D}}ominan \left( {\mathcal {T}}_{k}^{*},C\right) ^{-}}^{\beta \wedge l\left( o\right) }-\sum \limits _{k=1}^{\left| D\right| }\left( \underline{ S^{\Diamond }}\right) _{{\mathcal {D}}ominan\left( {\mathcal {T}} _{k}^{*},C\right) ^{-}}^{\beta \wedge l\left( o\right) }. \end{aligned}$$

#### Theorem 6

Let ${\mathcal {I}}{\mathcal {S}}^{*}=\left( A,C,D,E\right)$ be an incomplete information system and $S^{\Diamond }\subseteq A.$ If $\beta \in \left( 0.5,1 \right]$ and $l\in {\mathbb {N}} \cup \left\{ 0\right\} ,$ then the following hold:

1. $$\sum \nolimits _{k=1}^{\left| D\right| }\left( \underline{S^{\Diamond }}\right) _{{\mathcal {D}}ominan\left( {\mathcal {T}}_{k}^{*},C\right) ^{+}}^{\beta \wedge l\left( o\right) }=\sum \nolimits _{k=1}^{\left| D\right| }\left( \underline{S^{\Diamond }}\right) _{{\mathcal {D}}ominan\left( {\mathcal {T}}_{k}^{*},C\right) ^{+}}^{\beta \left( o\right) }\cap \sum \nolimits _{k=1}^{\left| D\right| }\left( \underline{S^{\Diamond }}\right) _{{\mathcal {D}}ominan \left( {\mathcal {T}}_{k}^{*},C\right) ^{+}}^{l\left( o\right) };$$
2. $$\sum \nolimits _{k=1}^{\left| D\right| }\left( \overline{S^{\Diamond }}\right) _{{\mathcal {D}}ominan\left( {\mathcal {T}}_{k}^{*},C\right) ^{+}}^{\beta \wedge l\left( o\right) }=\sum \nolimits _{k=1}^{\left| D\right| }\left( \overline{S^{\Diamond }} \right) _{{\mathcal {D}}ominan\left( {\mathcal {T}}_{k}^{*},C\right) ^{+}}^{\beta \left( o\right) }\cap \sum \nolimits _{k=1}^{\left| D\right| }\left( \overline{S^{\Diamond }}\right) _{{\mathcal {D}}ominan \left( {\mathcal {T}}_{k}^{*},C\right) ^{+}}^{l\left( o\right) };$$
3. $$\sum \nolimits _{k=1}^{\left| D\right| } \left( \underline{A}\right) _{{\mathcal {D}}ominan\left( {\mathcal {T}} _{k}^{*},C\right) ^{+}}^{\beta \wedge l\left( o\right) }=\sum \nolimits _{k=1}^{\left| D\right| }\left( \overline{A}\right) _{ {\mathcal {D}}ominan\left( {\mathcal {T}}_{k}^{*},C\right) ^{+}}^{\beta \wedge l\left( o\right) }=A;$$
4. $$\sum \nolimits _{k=1}^{\left| D\right| }\left( \underline{\emptyset }\right) _{{\mathcal {D}}ominan\left( {\mathcal {T}} _{k}^{*},C\right) ^{+}}^{\beta \wedge l\left( o\right) }=\sum \nolimits _{k=1}^{\left| D\right| }\left( \overline{\emptyset } \right) _{{\mathcal {D}}ominan\left( {\mathcal {T}}_{k}^{*},C\right) ^{+}}^{\beta \wedge l\left( o\right) }=\emptyset ;$$
5. $$\sum \nolimits _{k=1}^{\left| D\right| } \left( \underline{X^{\Diamond }}\right) _{{\mathcal {D}}ominan\left( {\mathcal {T}}_{k}^{*},C\right) ^{+}}^{\beta \wedge l_{1}\left( o\right) }\subseteq \sum \nolimits _{k=1}^{\left| D\right| }\left( \underline{X^{\Diamond }} \right) _{{\mathcal {D}}ominan\left( {\mathcal {T}}_{k}^{*},C\right) ^{+}}^{\beta \wedge l_{2}\left( o\right) },$$ $$\sum \nolimits _{k=1}^{\left| D\right| }\left( \overline{S^{\Diamond }}\right) _{{\mathcal {D}}ominan \left( {\mathcal {T}}_{k}^{*},C\right) ^{+}}^{\beta \wedge l_{2}\left( o\right) }\subseteq \sum \nolimits _{k=1}^{\left| D\right| }\left( \overline{S^{\Diamond }}\right) _{{\mathcal {D}}ominan\left( {\mathcal {T}}_{k}^{*},C\right) ^{+}}^{\beta \wedge l_{1}\left( o\right) },$$ if $$ l_{1}\le l_{2}\in {\mathbb {N}} \cup \left\{ 0\right\} ;$$
6. $$\sum \nolimits _{k=1}^{\left| D\right| }\left( \underline{S^{\Diamond }}\right) _{{\mathcal {D}}ominan\left( {\mathcal {T}}_{k}^{*},C\right) ^{+}}^{\beta _{2}\wedge l\left( o\right) }\subseteq \sum \nolimits _{k=1}^{\left| D\right| }\left( \underline{S^{\Diamond }} \right) _{{\mathcal {D}}ominan\left( {\mathcal {T}}_{k}^{*},C\right) ^{+}}^{\beta _{1}\wedge l\left( o\right) },$$ $$\sum \nolimits _{k=1}^{\left| D\right| }\left( \overline{S^{\Diamond }}\right) _{{\mathcal {D}}ominan \left( {\mathcal {T}}_{k}^{*},C\right) ^{+}}^{\beta _{1}\wedge l\left( o\right) }\subseteq \sum \nolimits _{k=1}^{\left| D\right| }\left( \overline{S^{\Diamond }}\right) _{{\mathcal {D}}ominan\left( {\mathcal {T}}_{k}^{*},C\right) ^{+}}^{\beta _{2}\wedge l\left( o\right) },$$ if $$ \beta _{1}\le \beta _{2}\in \left( 0.5,1\right] ;$$
7. $$\sum \nolimits _{k=1}^{\left| D\right| }\left( \underline{S^{\Diamond }}\right) _{{\mathcal {D}}ominan\left( {\mathcal {T}}_{k}^{*},C\right) ^{+}}^{\beta \wedge l\left( o\right) }\cup \sum \nolimits _{k=1}^{\left| D\right| }\left( \underline{P^{\Diamond }} \right) _{{\mathcal {D}}ominan\left( {\mathcal {T}}_{k}^{*},C\right) ^{+}}^{\beta \wedge l\left( o\right) }\subseteq \sum \nolimits _{k=1}^{\left| D\right| }\left( \underline{S^{\Diamond }\cup P^{\Diamond }}\right) _{{\mathcal {D}}ominan\left( {\mathcal {T}} _{k}^{*},C\right) ^{+}}^{\beta \wedge l\left( o\right) },$$ for all $$ S^{\Diamond },$$ $$P^{\Diamond }\subseteq A;$$
8. $$\sum \nolimits _{k=1}^{\left| D\right| }\left( \overline{S^{\Diamond }}\right) _{{\mathcal {D}}ominan\left( {\mathcal {T}}_{k}^{*},C\right) ^{+}}^{\beta \wedge l\left( o\right) }\cup \sum \nolimits _{k=1}^{\left| D\right| }\left( \overline{P^{\Diamond }} \right) _{{\mathcal {D}}ominan\left( {\mathcal {T}}_{k}^{*},C\right) ^{+}}^{\beta \wedge l\left( o\right) }\subseteq \sum \nolimits _{k=1}^{\left| D\right| }\left( \overline{S^{\Diamond }\cup P^{\Diamond }}\right) _{{\mathcal {D}}ominan\left( {\mathcal {T}} _{k}^{*},C\right) ^{+}}^{\beta \wedge l\left( o\right) },$$ for all $$ S^{\Diamond },$$ $$P^{\Diamond }\subseteq A;$$
9. $$\sum \nolimits _{k=1}^{\left| D\right| }\left( \underline{S^{\Diamond }}\right) _{{\mathcal {D}}ominan\left( {\mathcal {T}}_{k}^{*},C\right) ^{+}}^{\beta \wedge l\left( o\right) }\cap \sum \nolimits _{k=1}^{\left| D\right| }\left( \underline{P^{\Diamond }} \right) _{{\mathcal {D}}ominan\left( {\mathcal {T}}_{k}^{*},C\right) ^{+}}^{\beta \wedge l\left( o\right) }\supseteq \sum \nolimits _{k=1}^{\left| D\right| }\left( \underline{S^{\Diamond }\cap P^{\Diamond }}\right) _{{\mathcal {D}}ominan\left( {\mathcal {T}} _{k}^{*},C\right) ^{+}}^{\beta \wedge l\left( o\right) },$$ for all $$ S^{\Diamond },$$ $$P^{\Diamond }\subseteq A;$$
10. $$\sum \nolimits _{k=1}^{\left| D\right| }\left( \overline{S^{\Diamond }}\right) _{{\mathcal {D}}ominan\left( {\mathcal {T}}_{k}^{*},C\right) ^{+}}^{\beta \wedge l\left( o\right) }\cap \sum \nolimits _{k=1}^{\left| D\right| }\left( \overline{P^{\Diamond }} \right) _{{\mathcal {D}}ominan\left( {\mathcal {T}}_{k}^{*},C\right) ^{+}}^{\beta \wedge l\left( o\right) }\supseteq \sum \nolimits _{k=1}^{\left| D\right| }\left( \overline{S^{\Diamond }\cap P^{\Diamond }}\right) _{{\mathcal {D}}ominan\left( {\mathcal {T}} _{k}^{*},C\right) ^{+}}^{\beta \wedge l\left( o\right) },$$ for all $$ S^{\Diamond },$$ $$P^{\Diamond }\subseteq A.$$

#### Proof

The proof process is similar to the proof of Theorem 5. $\square$

#### Definition 9

Let ${\mathcal {I}}{\mathcal {S}}^{*}=\left( A,C,D,E\right)$ be an incomplete information system. If $S^{\Diamond }\subseteq A,$ $\beta \in \left( 0.5,1 \right]$ and $l\in {\mathbb {N}} \cup \left\{ 0\right\} ,$ then the approximate precisions $\sum \nolimits _{k=1}^{\left| D\right| }\left( S^{\Diamond }\right) _{ {\mathcal {D}}ominan\left( {\mathcal {T}}_{k}^{*},C\right) ^{+}}^{\rho \left( \beta \wedge l\right) \left( o\right) }$ and $\sum \nolimits _{k=1}^{\left| D\right| }\left( S^{\Diamond }\right) _{ {\mathcal {D}}ominan\left( {\mathcal {T}}_{k}^{*},C\right) ^{-}}^{\rho \left( \beta \wedge l\right) \left( o\right) }$ of $S^{\Diamond }$ about ${\mathcal {D}}ominan\left( {\mathcal {T}}_{k}^{*},C\right) ^{+}$ and ${\mathcal {D}}ominan\left( {\mathcal {T}}_{k}^{*},C\right) ^{-}$ are defined by:

$$\begin{aligned} \sum \limits _{k=1}^{\left| D\right| }\left( S^{\Diamond }\right) _{ {\mathcal {D}}ominan\left( {\mathcal {T}}_{k}^{*},C\right) ^{+}}^{\rho \left( \beta \wedge l\right) \left( o\right) }=\frac{\left| \sum \limits _{k=1}^{\left| D\right| }\left( \underline{S^{\Diamond }} \right) _{{\mathcal {D}}ominan\left( {\mathcal {T}}_{k}^{*},C\right) ^{+}}^{\beta \wedge l\left( o\right) }\right| }{\left| \sum \limits _{k=1}^{\left| D\right| }\left( \overline{S^{\Diamond }} \right) _{{\mathcal {D}}ominan\left( {\mathcal {T}}_{k}^{*},C\right) ^{+}}^{\beta \wedge l\left( o\right) }\right| }; \end{aligned}$$

and

$$\begin{aligned} \ \sum \limits _{k=1}^{\left| D\right| }\left( S^{\Diamond }\right) _{{\mathcal {D}}ominan\left( {\mathcal {T}}_{k}^{*},C\right) ^{-}}^{\rho \left( \beta \wedge l\right) \left( o\right) }=\frac{\left| \sum \limits _{k=1}^{\left| D\right| }\left( \underline{S^{\Diamond }} \right) _{{\mathcal {D}}ominan\left( {\mathcal {T}}_{k}^{*},C\right) ^{-}}^{\beta \wedge l\left( o\right) }\right| }{\left| \sum \limits _{k=1}^{\left| D\right| }\left( \overline{S^{\Diamond }} \right) _{{\mathcal {D}}ominan\left( {\mathcal {T}}_{k}^{*},C\right) ^{-}}^{\beta \wedge l\left( o\right) }\right| }, \end{aligned}$$

where $S^{\Diamond }\ne \emptyset ,$ and $\left| \cdot \right|$ denotes the cardinality of a set.

Let $\sum \nolimits _{k=1}^{\left| D\right| }\left( S^{\Diamond }\right) _{{\mathcal {D}}ominan\left( {\mathcal {T}}_{k}^{*},C\right) ^{+}}^{\mu \left( \beta \wedge l\right) \left( o\right) }=1-\sum \nolimits _{k=1}^{\left| D\right| }\left( S^{\Diamond }\right) _{{\mathcal {D}}ominan\left( {\mathcal {T}}_{k}^{*},C\right) ^{+}}^{\rho \left( \beta \wedge l\right) \left( o\right) }$ and $\sum \nolimits _{k=1}^{\left| D\right| }\left( S^{\Diamond }\right) _{ {\mathcal {D}}ominan\left( {\mathcal {T}}_{k}^{*},C\right) ^{-}}^{\mu \left( \beta \wedge l\right) \left( o\right) }=1-\sum \nolimits _{k=1}^{\left| D\right| }\left( S^{\Diamond }\right) _{{\mathcal {D}}ominan\left( {\mathcal {T}}_{k}^{*},C\right) ^{-}}^{\rho \left( \beta \wedge l\right) \left( o\right) },$ then $\sum \nolimits _{k=1}^{\left| D\right| }\left( S^{\Diamond }\right) _{ {\mathcal {D}}ominan\left( {\mathcal {T}}_{k}^{*},C\right) ^{+}}^{\mu \left( \beta \wedge l\right) \left( o\right) }$ and $\sum \nolimits _{k=1}^{ \left| D\right| }\left( S^{\Diamond }\right) _{{\mathcal {D}}ominan \left( {\mathcal {T}}_{k}^{*},C\right) ^{-}}^{\mu \left( \beta \wedge l\right) \left( o\right) }$ are called the rough degrees of $S^{\Diamond }$ about ${\mathcal {D}}ominan\left( {\mathcal {T}}_{k}^{*},C\right) ^{+}$ and ${\mathcal {D}}ominan\left( {\mathcal {T}}_{k}^{*},C\right) ^{-}$ respectively.

By definition $0\le \sum \nolimits _{k=1}^{\left| D\right| }\left( S^{\Diamond }\right) _{{\mathcal {D}}ominan\left( {\mathcal {T}} _{k}^{*},C\right) ^{+}}^{\mu \left( \beta \wedge l\right) \left( o\right) }\le 1$ and $0\le \sum \nolimits _{k=1}^{\left| D\right| }\left( S^{\Diamond }\right) _{{\mathcal {D}}ominan\left( {\mathcal {T}} _{k}^{*},C\right) ^{-}}^{\mu \left( \beta \wedge l\right) \left( o\right) }\le 1.$

#### Definition 10

Let ${\mathcal {I}}{\mathcal {S}}^{*}=\left( A,C,D,E\right)$ be an incomplete information system and $S^{\Diamond }\subseteq A,$ $\beta \in \left( 0.5,1 \right]$ and $l\in {\mathbb {N}} \cup \left\{ 0\right\} .$ Then the *approximate quality* $\sum \nolimits _{k=1}^{\left| D\right| }\left( S^{\Diamond }\right) _{ {\mathcal {D}}ominan\left( {\mathcal {T}}_{k}^{*},C\right) ^{+}}^{\gamma \left( \beta \wedge l\right) \left( o\right) }$ and $\sum \nolimits _{k=1}^{\left| D\right| }\left( S^{\Diamond }\right) _{ {\mathcal {D}}ominan\left( {\mathcal {T}}_{k}^{*},C\right) ^{-}}^{\gamma \left( \beta \wedge l\right) \left( o\right) }$ of $S^{\Diamond }$ about ${\mathcal {D}}ominan\left( {\mathcal {T}}_{k}^{*},C\right) ^{+}$ and ${\mathcal {D}}ominan\left( {\mathcal {T}}_{k}^{*},C\right) ^{-}$ are defined by:

$$\begin{aligned} \sum \limits _{k=1}^{\left| D\right| }\left( S^{\Diamond }\right) _{ {\mathcal {D}}ominan\left( {\mathcal {T}}_{k}^{*},C\right) ^{+}}^{\gamma \left( \beta \wedge l\right) \left( o\right) }=\frac{ \left| \sum \limits _{k=1}^{\left| D\right| }\left( \underline{ S^{\Diamond }}\right) _{{\mathcal {D}}ominan\left( {\mathcal {T}} _{k}^{*},C\right) ^{+}}^{\beta \wedge l\left( o\right) }\right| }{ \left| A\right| } \end{aligned}$$

and

$$\begin{aligned} \sum \limits _{k=1}^{\left| D\right| }\left( S^{\Diamond }\right) _{ {\mathcal {D}}ominan\left( {\mathcal {T}}_{k}^{*},C\right) ^{-}}^{\gamma \left( \beta \wedge l\right) \left( o\right) }=\frac{ \left| \sum \limits _{k=1}^{\left| D\right| }\left( \underline{ S^{\Diamond }}\right) _{{\mathcal {D}}ominan\left( {\mathcal {T}} _{k}^{*},C\right) ^{-}}^{\beta \wedge l\left( o\right) }\right| }{ \left| A\right| }. \end{aligned}$$

Clearly $$0\le \sum \nolimits _{k=1}^{\left| D\right| }\left( S^{\Diamond }\right) _{{\mathcal {D}}ominan\left( {\mathcal {T}} _{k}^{*},C\right) ^{+}}^{\gamma \left( \beta \wedge l\right) \left( o\right) }\le 1$$ and $$0\le \sum \nolimits _{k=1}^{\left| D\right| }\left( S^{\Diamond }\right) _{{\mathcal {D}}ominan\left( {\mathcal {T}} _{k}^{*},C\right) ^{-}}^{\gamma \left( \beta \wedge l\right) \left( o\right) }\le 1.$$

We now obtain three way decision rules using logical conjunction soft dominating optimistic multigranulation rough sets:

$\left( \mathrm{i}\right)$ If there exists $k\in \left\{ 1,2,3,...,\left| D\right| \right\}$ such that $\left( \frac{ \left| \left[ b\right] _{{\mathcal {D}}ominan\left( {\mathcal {T}} _{k}^{*},C\right) }^{+}\cap S^{\Diamond }\right| }{\left| \left[ b\right] _{{\mathcal {D}}ominan\left( {\mathcal {T}}_{k}^{*},C\right) }^{+}\right| }\right) >\beta \wedge \left( \left| \left[ b\right] _{ {\mathcal {D}}ominan\left( {\mathcal {T}}_{k}^{*},C\right) }^{+}\right| -\left| \left[ b\right] _{{\mathcal {D}}ominan\left( {\mathcal {T}}_{k}^{*},C\right) }^{+}\cap S^{\Diamond }\right| \right) <l,$ decide $Pos\left( S^{\Diamond }\right) _{{\mathcal {D}}ominan \left( {\mathcal {T}}_{k}^{*},C\right) ^{+}}^{\beta \wedge l\left( o\right) };$

$\left( \mathrm{ii}\right)$ If there exists for all $k\in \left\{ 1,2,3,...,\left| D\right| \right\}$ such that $\left( \frac{ \left| \left[ b\right] _{{\mathcal {D}}ominan\left( {\mathcal {T}} _{k}^{*},C\right) }^{+}\cap S^{\Diamond }\right| }{\left| \left[ b\right] _{{\mathcal {D}}ominan\left( {\mathcal {T}}_{k}^{*},C\right) }^{+}\right| }\right)<1-\beta \wedge \left( \left| \left[ b\right] _{{\mathcal {D}}ominan\left( {\mathcal {T}}_{k}^{*},C\right) }^{+}\right| -\left| \left[ b\right] _{{\mathcal {D}}ominan\left( {\mathcal {T}}_{k}^{*},C\right) }^{+}\cap S^{\Diamond }\right| \right) <l,$ decide $Neg\left( S^{\Diamond }\right) _{{\mathcal {D}}ominan \left( {\mathcal {T}}_{k}^{*},C\right) ^{+}}^{\beta \wedge l\left( o\right) };$

$$\left( \mathrm{iii}\right) $$ Otherwise, decide $$\sum \nolimits _{k=1}^{\left| D\right| }\left( S^{\Diamond }\right) _{{\mathcal {D}}ominan\left( {\mathcal {T}}_{k}^{*},C\right) ^{+}}^{\beta \vee l\left( o\right) }.$$

Similarly, we obtain three way decision rules using logical conjunction soft dominated optimistic multigranulation rough sets:

$\left( \mathrm{i}\right)$ If there exists $k\in \left\{ 1,2,3,...,\left| D\right| \right\}$ such that $\left( \frac{ \left| \left[ b\right] _{{\mathcal {D}}ominan\left( {\mathcal {T}} _{k}^{*},C\right) }^{-}\cap S^{\Diamond }\right| }{\left| \left[ b\right] _{{\mathcal {D}}ominan\left( {\mathcal {T}}_{k}^{*},C\right) }^{-}\right| }\right) >\beta \wedge \left( \left| \left[ b\right] _{ {\mathcal {D}}ominan\left( {\mathcal {T}}_{k}^{*},C\right) }^{-}\right| -\left| \left[ b\right] _{{\mathcal {D}}ominan\left( {\mathcal {T}}_{k}^{*},C\right) }^{-}\cap S^{\Diamond }\right| \right) <l,$ decide $Pos\left( S^{\Diamond }\right) _{{\mathcal {D}}ominan \left( {\mathcal {T}}_{k}^{*},C\right) ^{-}}^{\beta \wedge l\left( o\right) };$

$\left( \mathrm{ii}\right)$ If there exists for all $k\in \left\{ 1,2,3,...,\left| D\right| \right\}$ such that $\left( \frac{ \left| \left[ b\right] _{{\mathcal {D}}ominan\left( {\mathcal {T}} _{k}^{*},C\right) }^{-}\cap S^{\Diamond }\right| }{\left| \left[ b\right] _{{\mathcal {D}}ominan\left( {\mathcal {T}}_{k}^{*},C\right) }^{-}\right| }\right)<1-\beta \wedge \left( \left| \left[ b\right] _{{\mathcal {D}}ominan\left( {\mathcal {T}}_{k}^{*},C\right) }^{-}\right| -\left| \left[ b\right] _{{\mathcal {D}}ominan\left( {\mathcal {T}}_{k}^{*},C\right) }^{-}\cap S^{\Diamond }\right| \right) <l,$ decide $Neg\left( S^{\Diamond }\right) _{{\mathcal {D}}ominan \left( {\mathcal {T}}_{k}^{*},C\right) ^{-}}^{\beta \wedge l\left( o\right) };$

$$\left( \mathrm{iii}\right) $$ Otherwise, decide $$\sum \nolimits _{k=1}^{\left| D\right| }\left( S^{\Diamond }\right) _{{\mathcal {D}}ominan\left( {\mathcal {T}}_{k}^{*},C\right) ^{-}}^{\beta \wedge l\left( o\right) }.$$

### Proposed Algorithm-$\mathrm{II}$ for conflict analysis model

**Input:** Given information system ${\mathcal {I}}{\mathcal {S}}^{*}=\left( A,C,D,E\right) ;$

**Step** 1: Construct $\left( {\mathcal {T}}_{k}^{*},C\right)$ corresponding to the $D_{k}$ for all $k=1,2,...,\left| D\right|$;

**Step** 2: Construct ${\mathcal {D}}ominan\left( {\mathcal {T}} _{k}^{*},C\right) ,$ for all $k=1,2,...,\left| D\right|$;

**Step** 3: Construct $\sum \nolimits _{k=1}^{\left| D\right| }\left( \underline{S_{k}^{\Diamond }}\right) _{{\mathcal {D}}ominan\left( {\mathcal {T}}_{k}^{*},C\right) ^{+}}^{\beta \wedge l\left( o\right) }$ and $\sum \nolimits _{k=1}^{\left| D\right| }\left( \underline{S_{k}^{\Diamond }}\right) _{{\mathcal {D}}ominan\left( {\mathcal {T}}_{k}^{*},C\right) ^{-}}^{\beta \wedge l\left( o\right) }$, where $S^{\Diamond }=\left\{ b\in A:g(b,D_{k})\ne 0\right\} ,$ $\beta \in \left( 0.5,1\right]$ and $l\in {\mathbb {N}} \cup \left\{ 0\right\} ;$

**Step** 4: If $\sum \nolimits _{k=1}^{\left| D\right| }\left( \underline{S_{k}^{\Diamond }}\right) _{{\mathcal {D}}ominan\left( {\mathcal {T}}_{k}^{*},C\right) ^{+}}^{\beta \wedge l\left( o\right) }=\sum \nolimits _{k=1}^{\left| D\right| }\left( \underline{ S_{k}^{\Diamond }}\right) _{{\mathcal {D}}ominan\left( {\mathcal {T}} _{k}^{*},C\right) ^{-}}^{\beta \wedge l\left( o\right) }$ then $\delta _{k}=\sum \nolimits _{k=1}^{\left| D\right| }\left( \underline{ S_{k}^{\Diamond }}\right) _{{\mathcal {D}}ominan\left( {\mathcal {T}} _{k}^{*},C\right) ^{+}}^{\beta \wedge l\left( o\right) }$ or $\delta _{k}=\sum \nolimits _{k=1}^{\left| D\right| }\left( \underline{ S_{k}^{\Diamond }}\right) _{{\mathcal {D}}ominan\left( {\mathcal {T}} _{k}^{*},C\right) ^{-}}^{\beta \wedge l\left( o\right) }$ otherwise $\delta _{k}=\sum \nolimits _{k=1}^{\left| D\right| }\left( \underline{ S_{k}^{\Diamond }}\right) _{{\mathcal {D}}ominan\left( {\mathcal {T}} _{k}^{*},C\right) ^{+}}^{\beta \wedge l\left( o\right) }-\sum \nolimits _{k=1}^{\left| D\right| }\left( \underline{ S_{k}^{\Diamond }}\right) _{{\mathcal {D}}ominan\left( {\mathcal {T}} _{k}^{*},C\right) ^{-}}^{\beta \wedge l\left( o\right) };$

**Step** 5: $$\delta =\bigcap \nolimits _{k=1}^{\left| D\right| }\delta _{k};$$

**Step** 6: If $$\delta \ne \emptyset ;$$

         go to **output.**

         otherwise,

         put $\beta ^{\prime }$ in **Step** 3, where $\beta ^{\prime }\le \beta ,$

or

         put $l^{\prime }$ in **Step** 3, where $l\le l^{\prime };$

**Output:** Feasible alternative(s) for feasible consensus strategy.

#### Example 3

(Continued from Example 1) Let ${\mathcal {I}}{\mathcal {S}}^{*}=\left( A,C,D,E\right)$ be an incomplete information system. Applying the Algorithm-$\mathrm{II}$, we have

Using Tables 8 and 9 to get $\delta =\left\{ b_{7}\right\} .$ The result shows that $b_{7}$ is the optimal decision medicine with decision precision 0.9. Since the action $b_{7}$ is the medicine for solving this conflict analysis problem on which most of the doctors have agreed in the conflict situation ${\mathcal {I}}{\mathcal {S}}^{*}.$

**Table 8:** The lower approximations with $\beta =0.90, l=1$.

$S^{\Diamond }$	$\sum \limits _{k=1}^{\left| D\right| }\left( \underline{S_{k}^{\Diamond }}\right) _{{\mathcal {D}}ominan \left( {\mathcal {T}}_{k}^{*},C\right) ^{+}}^{\beta \wedge l\left( o\right) }$	$\sum \nolimits _{k=1}^{\left| D\right| }\left( \underline{S_{k}^{\Diamond }}\right) _{{\mathcal {D}}om inan\left( {\mathcal {T}}_{k}^{*},C\right) ^{-}}^{\beta \wedge l\left( o\right) }$
$S_{1}^{\Diamond }$	$\left\{ b_{1},b_{2},b_{3},b_{4},b_{6},b_{7},b_{9},b_{10},b_{12}\right\}$	$\left\{ b_{1},b_{2},b_{3},b_{4},b_{6},b_{12}\right\}$
$S_{2}^{\Diamond }$	$\left\{ b_{1},b_{2},b_{3},b_{4},b_{5},b_{7},b_{8},b_{9},b_{10},b_{11},b_{12}\right\}$	$\left\{ b_{1},b_{2},b_{3},b_{4},b_{5},b_{8},b_{9},b_{10},b_{11},b_{12}\right\}$
$S_{3}^{\Diamond }$	$\left\{ b_{1},b_{6},b_{7},b_{9},b_{11},b_{12}\right\}$	$\left\{ b_{1},b_{4},b_{6},b_{11},b_{12}\right\}$
$S_{4}^{\Diamond }$	$\left\{ b_{1},b_{2},b_{3},b_{4},b_{5},b_{7},b_{8},b_{9},b_{10},b_{12}\right\}$	$\left\{ b_{1},b_{2},b_{3},b_{4},b_{5},b_{8},b_{9},b_{10},b_{12}\right\}$

**Table 9:** Decision table with $\beta =0.90, l=1$.

$\delta _{k}$	$\sum \limits _{k=1}^{\left| D\right| }\left( \underline{S_{k}^{\Diamond }}\right) _{{\mathcal {D}} ominan\left( {\mathcal {T}}_{k}^{*},C\right) ^{+}}^{\beta \wedge l\left( o\right) }-\sum \limits _{k=1}^{\left| D\right| }\left( \underline{S_{k}^{\Diamond }}\right) _{{\mathcal {D}}ominan\left( {\mathcal {T}}_{k}^{*},C\right) ^{-}}^{\beta \wedge l\left( o\right) }$
$\delta _{1}$	$\left\{ b_{7},b_{9},b_{10}\right\}$
$\delta _{2}$	$\left\{ b_{7}\right\}$
$\delta _{3}$	$\left\{ b_{7},b_{9}\right\}$
$\delta _{4}$	$\left\{ b_{7}\right\}$

### Logical disjunction soft dominating/dominated pessimistic multigranulation rough sets

#### Definition 11

Let ${\mathcal {I}}{\mathcal {S}}^{*}=\left( A,C,D,E\right)$ be an incomplete information system and $S^{\Diamond }\subseteq A.$ If $\beta \in \left( 0.5,1\right]$ and $l\in {\mathbb {N}} \cup \left\{ 0\right\} ,$ then the pessimistic lower approximation and pessimistic upper approximation of $S^{\Diamond }$ with respect to ${\mathcal {D}}ominan\left( {\mathcal {T}}_{k}^{*},C\right) ^{+}$ are defined by

$$\begin{aligned} \sum \limits _{k=1}^{\left| D\right| }\left( \underline{S^{\Diamond }} \right) _{{\mathcal {D}}ominan\left( {\mathcal {T}}_{k}^{*},C\right) ^{+}}^{\beta \vee l\left( p\right) }=\bigwedge \limits _{k=1}^{\left| D\right| }\left\{ \begin{array}{c} b\in A\mid \left( \frac{\left| \left[ b\right] _{{\mathcal {D}}ominan \left( {\mathcal {T}}_{k}^{*},C\right) }^{+}\cap S^{\Diamond }\right| }{\left| \left[ b\right] _{{\mathcal {D}}ominan\left( {\mathcal {T}} _{k}^{*},C\right) }^{+}\right| }\right) >\beta \\ \vee \left( \left| \left[ b\right] _{{\mathcal {D}}ominan\left( {\mathcal {T}}_{k}^{*},C\right) }^{+}\right| -\left| \left[ b\right] _{{\mathcal {D}}ominan\left( {\mathcal {T}}_{k}^{*},C\right) }^{+}\cap S^{\Diamond }\right| \right) <l \end{array}\right\} ; \end{aligned}$$

and

$$\begin{aligned} \sum \limits _{k=1}^{\left| D\right| }\left( \overline{S^{\Diamond }} \right) _{{\mathcal {D}}ominan\left( {\mathcal {T}}_{k}^{*},C\right) ^{+}}^{\beta \vee l\left( p\right) }=\bigvee \limits _{k=1}^{\left| D\right| }\left\{ \begin{array}{c} b\in A\mid \left( \frac{\left| \left[ b\right] _{{\mathcal {D}}ominan \left( {\mathcal {T}}_{k}^{*},C\right) }^{+}\cap S^{\Diamond }\right| }{\left| \left[ b\right] _{{\mathcal {D}}ominan\left( {\mathcal {T}} _{k}^{*},C\right) }^{+}\right| }\right) \ge 1-\beta \\ \vee \left( \left| \left[ b\right] _{{\mathcal {D}}ominan\left( {\mathcal {T}}_{k}^{*},C\right) }^{+}\cap S^{\Diamond }\right| \right) \ge l \end{array}\right\} . \end{aligned}$$

The pair $\left( \sum \nolimits _{k=1}^{\left| D\right| }\left( \underline{S^{\Diamond }}\right) _{{\mathcal {D}}ominan\left( {\mathcal {T}}_{k}^{*},C\right) ^{+}}^{\beta \vee l\left( p\right) },\sum \nolimits _{k=1}^{\left| D\right| }\left( \overline{S^{\Diamond }} \right) _{{\mathcal {D}}ominan\left( {\mathcal {T}}_{k}^{*},C\right) ^{+}}^{\beta \vee l\left( p\right) }\right)$ is called the logical disjunction soft dominating pessimistic multigranulation rough set if $\sum \nolimits _{k=1}^{\left| D\right| }\left( \underline{S^{\Diamond }} \right) _{{\mathcal {D}}ominan\left( {\mathcal {T}}_{k}^{*},C\right) ^{+}}^{\beta \vee l\left( o\right) }\ne \sum \nolimits _{k=1}^{\left| D\right| }\left( \overline{S^{\Diamond }}\right) _{{\mathcal {D}}ominan \left( {\mathcal {T}}_{k}^{*},C\right) ^{+}}^{\beta \vee l\left( o\right) }.$ Similarly the pessimistic lower approximation and pessimistic upper approximation of $S^{\Diamond }$ with respect to ${\mathcal {D}}ominan\left( {\mathcal {T}}_{k}^{*},C\right) ^{-}$ are defined by

$$\begin{aligned} \sum \limits _{k=1}^{\left| D\right| }\left( \underline{S^{\Diamond }} \right) _{{\mathcal {D}}ominan\left( {\mathcal {T}}_{k}^{*},C\right) ^{-}}^{\beta \vee l\left( p\right) }=\bigwedge \limits _{k=1}^{\left| D\right| }\left\{ \begin{array}{c} b\in A\mid \left( \frac{\left| \left[ b\right] _{{\mathcal {D}}ominan \left( {\mathcal {T}}_{k}^{*},C\right) }^{-}\cap S^{\Diamond }\right| }{\left| \left[ b\right] _{{\mathcal {D}}ominan\left( {\mathcal {T}} _{k}^{*},C\right) }^{-}\right| }\right) >\beta \\ \vee \left( \left| \left[ b\right] _{{\mathcal {D}}ominan\left( {\mathcal {T}}_{k}^{*},C\right) }^{-}\right| -\left| \left[ b\right] _{{\mathcal {D}}ominan\left( {\mathcal {T}}_{k}^{*},C\right) }^{-}\cap S^{\Diamond }\right| \right) <l \end{array}\right\} ; \end{aligned}$$

and

$$\begin{aligned} \sum \limits _{k=1}^{\left| D\right| }\left( \overline{S^{\Diamond }} \right) _{{\mathcal {D}}ominan\left( {\mathcal {T}}_{k}^{*},C\right) ^{-}}^{\beta \vee l\left( p\right) }=\bigvee \limits _{k=1}^{\left| D\right| }\left\{ \begin{array}{c} b\in A\mid \left( \frac{\left| \left[ b\right] _{{\mathcal {D}}ominan \left( {\mathcal {T}}_{k}^{*},C\right) }^{-}\cap S^{\Diamond }\right| }{\left| \left[ b\right] _{{\mathcal {D}}ominan\left( {\mathcal {T}} _{k}^{*},C\right) }^{-}\right| }\right) \ge 1-\beta \\ \vee \left( \left| \left[ b\right] _{{\mathcal {D}}ominan\left( {\mathcal {T}}_{k}^{*},C\right) }^{-}\cap S^{\Diamond }\right| \right) \ge l \end{array}\right\} . \end{aligned}$$

The pair $\left( \sum \nolimits _{k=1}^{\left| D\right| }\left( \underline{S^{\Diamond }}\right) _{{\mathcal {D}}ominan\left( {\mathcal {T}}_{k}^{*},C\right) ^{-}}^{\beta \vee l\left( p\right) },\sum \nolimits _{k=1}^{\left| D\right| }\left( \overline{S^{\Diamond }} \right) _{{\mathcal {D}}ominan\left( {\mathcal {T}}_{k}^{*},C\right) ^{-}}^{\beta \vee l\left( p\right) }\right)$ is called the logical disjunction soft dominated pessimistic multigranulation rough set if $\sum \nolimits _{k=1}^{\left| D\right| }\left( \underline{S^{\Diamond }} \right) _{{\mathcal {D}}ominan\left( {\mathcal {T}}_{k}^{*},C\right) ^{-}}^{\beta \vee l\left( p\right) }\ne \sum \nolimits _{k=1}^{\left| D\right| }\left( \overline{S^{\Diamond }}\right) _{{\mathcal {D}}ominan \left( {\mathcal {T}}_{k}^{*},C\right) ^{-}}^{\beta \vee l\left( p\right) }.$ Using the lower approximation $\sum \nolimits _{k=1}^{\left| D\right| }\left( \underline{S^{\Diamond }}\right) _{{\mathcal {D}}ominan \left( {\mathcal {T}}_{k}^{*},C\right) ^{+}}^{\beta \vee l\left( p\right) }$ and upper approximation $\sum \nolimits _{k=1}^{\left| D\right| }\left( \overline{S^{\Diamond }}\right) _{{\mathcal {D}}ominan \left( {\mathcal {T}}_{k}^{*},C\right) ^{+}}^{\beta \vee l\left( p\right) },$ the logical disjunction soft dominating pessimistic multigranulation boundary region of $S^{\Diamond }$ is

$$\begin{aligned} \sum \limits _{k=1}^{\left| D\right| }\left( S^{\Diamond }\right) _{ {\mathcal {D}}ominan\left( {\mathcal {T}}_{k}^{*},C\right) ^{+}}^{\beta \vee l\left( p\right) }=\sum \limits _{k=1}^{\left| D\right| }\left( \overline{S^{\Diamond }}\right) _{{\mathcal {D}}ominan \left( {\mathcal {T}}_{k}^{*},C\right) ^{+}}^{\beta \vee l\left( p\right) }-\sum \limits _{k=1}^{\left| D\right| }\left( \underline{ S^{\Diamond }}\right) _{{\mathcal {D}}ominan\left( {\mathcal {T}} _{k}^{*},C\right) ^{+}}^{\beta \vee l\left( p\right) }. \end{aligned}$$

Similarly the lower approximation $\sum \nolimits _{k=1}^{\left| D\right| }\left( \underline{X}\right) _{{\mathcal {D}}ominan\left( {\mathcal {T}}_{k}^{*},C\right) ^{-}}^{\beta \vee l\left( p\right) }$ and upper approximation $\sum \nolimits _{k=1}^{\left| D\right| }\left( \overline{S^{\Diamond }}\right) _{{\mathcal {D}}ominan\left( {\mathcal {T}}_{k}^{*},C\right) ^{-}}^{\beta \vee l\left( p\right) },$ the logical disjunction soft dominated pessimistic multigranulation boundary region of $S^{\Diamond }$ is

$$\begin{aligned} \sum \limits _{k=1}^{\left| D\right| }\left( S^{\Diamond }\right) _{ {\mathcal {D}}ominan\left( {\mathcal {T}}_{k}^{*},C\right) ^{-}}^{\beta \vee l\left( p\right) }=\sum \limits _{k=1}^{\left| D\right| }\left( \overline{S^{\Diamond }}\right) _{{\mathcal {D}}ominan \left( {\mathcal {T}}_{k}^{*},C\right) ^{-}}^{\beta \vee l\left( p\right) }-\sum \limits _{k=1}^{\left| D\right| }\left( \underline{ S^{\Diamond }}\right) _{{\mathcal {D}}ominan\left( {\mathcal {T}} _{k}^{*},C\right) ^{-}}^{\beta \vee l\left( p\right) }. \end{aligned}$$

#### Theorem 7

Let ${\mathcal {I}}{\mathcal {S}}^{*}=\left( A,C,D,E\right)$ be an incomplete information system and $S^{\Diamond }\subseteq A.$ If $\beta \in \left( 0.5,1 \right]$ and $l\in {\mathbb {N}} \cup \left\{ 0\right\} ,$ then the following hold:

1. $$\sum \nolimits _{k=1}^{\left| D\right| }\left( \underline{S^{\Diamond }}\right) _{{\mathcal {D}}ominan\left( {\mathcal {T}}_{k}^{*},C\right) ^{+}}^{\beta \vee l\left( p\right) }=\sum \nolimits _{k=1}^{\left| D\right| }\left( \underline{S^{\Diamond }}\right) _{{\mathcal {D}}ominan\left( {\mathcal {T}}_{k}^{*},C\right) ^{+}}^{\beta \left( p\right) }\cup \sum \nolimits _{k=1}^{\left| D\right| }\left( \underline{S^{\Diamond }}\right) _{{\mathcal {D}}ominan \left( {\mathcal {T}}_{k}^{*},C\right) ^{+}}^{l\left( p\right) };$$
2. $$\sum \nolimits _{k=1}^{\left| D\right| }\left( \overline{S^{\Diamond }}\right) _{{\mathcal {D}}ominan\left( {\mathcal {T}}_{k}^{*},C\right) ^{+}}^{\beta \vee l\left( p\right) }=\sum \nolimits _{k=1}^{\left| D\right| }\left( \overline{S^{\Diamond }} \right) _{{\mathcal {D}}ominan\left( {\mathcal {T}}_{k}^{*},C\right) ^{+}}^{\beta \left( p\right) }\cup \sum \nolimits _{k=1}^{\left| D\right| }\left( \overline{S^{\Diamond }}\right) _{{\mathcal {D}}ominan \left( {\mathcal {T}}_{k}^{*},C\right) ^{+}}^{l\left( p\right) };$$
3. $$\sum \nolimits _{k=1}^{\left| D\right| }\left( \underline{A}\right) _{{\mathcal {D}}ominan\left( {\mathcal {T}} _{k}^{*},C\right) ^{+}}^{\beta \vee l\left( p\right) }=\sum \nolimits _{k=1}^{\left| D\right| }\left( \overline{A}\right) _{ {\mathcal {D}}ominan\left( {\mathcal {T}}_{k}^{*},C\right) ^{+}}^{\beta \vee l\left( p\right) }=A;$$
4. $$\sum \nolimits _{k=1}^{\left| D\right| }\left( \underline{\emptyset }\right) _{{\mathcal {D}}ominan\left( {\mathcal {T}} _{k}^{*},C\right) ^{+}}^{\beta \vee l\left( p\right) }=\sum \nolimits _{k=1}^{\left| D\right| }\left( \overline{\emptyset } \right) _{{\mathcal {D}}ominan\left( {\mathcal {T}}_{k}^{*},C\right) ^{+}}^{\beta \vee l\left( p\right) }=\emptyset ;$$
5. $$\sum \nolimits _{k=1}^{\left| D\right| }\left( \underline{S^{\Diamond }}\right) _{{\mathcal {D}}ominan\left( {\mathcal {T}}_{k}^{*},C\right) ^{+}}^{\beta \vee l_{1}\left( p\right) }\subseteq \sum \nolimits _{k=1}^{\left| D\right| }\left( \underline{S^{\Diamond }} \right) _{{\mathcal {D}}ominan\left( {\mathcal {T}}_{k}^{*},C\right) ^{+}}^{\beta \vee l_{2}\left( p\right) },$$ $$\sum \nolimits _{k=1}^{\left| D\right| }\left( \overline{S^{\Diamond }}\right) _{{\mathcal {D}}ominan \left( {\mathcal {T}}_{k}^{*},C\right) ^{+}}^{\beta \vee l_{2}\left( p\right) }\subseteq \sum \nolimits _{k=1}^{\left| D\right| }\left( \overline{S^{\Diamond }}\right) _{{\mathcal {D}}ominan\left( {\mathcal {T}}_{k}^{*},C\right) ^{+}}^{\beta \vee l_{1}\left( p\right) },$$ if $$ l_{1}\le l_{2}\in {\mathbb {N}} \cup \left\{ 0\right\} ;$$
6. $$\sum \nolimits _{k=1}^{\left| D\right| }\left( \underline{S^{\Diamond }}\right) _{{\mathcal {D}}ominan\left( {\mathcal {T}}_{k}^{*},C\right) ^{+}}^{\beta _{2}\vee l\left( p\right) }\subseteq \sum \nolimits _{k=1}^{\left| D\right| }\left( \underline{S^{\Diamond }} \right) _{{\mathcal {D}}ominan\left( {\mathcal {T}}_{k}^{*},C\right) ^{+}}^{\beta _{1}\vee l\left( p\right) },$$ $$\sum \nolimits _{k=1}^{\left| D\right| }\left( \overline{S^{\Diamond }}\right) _{{\mathcal {D}}ominan \left( {\mathcal {T}}_{k}^{*},C\right) ^{+}}^{\beta _{1}\vee l\left( p\right) }\subseteq \sum \nolimits _{k=1}^{\left| D\right| }\left( \overline{S^{\Diamond }}\right) _{{\mathcal {D}}ominan\left( {\mathcal {T}}_{k}^{*},C\right) ^{+}}^{\beta _{2}\vee l\left( p\right) },$$ if $$\beta _{1}\le \beta _{2}\in \left( 0.5,1\right] ;$$
7. $$\sum \nolimits _{k=1}^{\left| D\right| }\left( \underline{S^{\Diamond }}\right) _{{\mathcal {D}}ominan\left( {\mathcal {T}}_{k}^{*},C\right) ^{+}}^{\beta \vee l\left( p\right) }\cup \sum \nolimits _{k=1}^{\left| D\right| }\left( \underline{P^{\Diamond }} \right) _{{\mathcal {D}}ominan\left( {\mathcal {T}}_{k}^{*},C\right) ^{+}}^{\beta \vee l\left( p\right) }\subseteq \sum \nolimits _{k=1}^{\left| D\right| }\left( \underline{S^{\Diamond }\cup P^{\Diamond }}\right) _{ {\mathcal {D}}ominan\left( {\mathcal {T}}_{k}^{*},C\right) ^{+}}^{\beta \vee l\left( p\right) },$$ for all $$S^{\Diamond },$$ $$P^{\Diamond }\subseteq A;$$
8. $$\sum \nolimits _{k=1}^{\left| D\right| }\left( \overline{S^{\Diamond }}\right) _{{\mathcal {D}}ominan\left( {\mathcal {T}}_{k}^{*},C\right) ^{+}}^{\beta \vee l\left( p\right) }\cup \sum \nolimits _{k=1}^{\left| D\right| }\left( \overline{P^{\Diamond }} \right) _{{\mathcal {D}}ominan\left( {\mathcal {T}}_{k}^{*},C\right) ^{+}}^{\beta \vee l\left( p\right) }\subseteq \sum \nolimits _{k=1}^{\left| D\right| }\left( \overline{S^{\Diamond }\cup P^{\Diamond }}\right) _{ {\mathcal {D}}ominan\left( {\mathcal {T}}_{k}^{*},C\right) ^{+}}^{\beta \vee l\left( p\right) },$$ for all $$S^{\Diamond },$$ $$P^{\Diamond }\subseteq A;$$
9. $$\sum \nolimits _{k=1}^{\left| D\right| }\left( \underline{S^{\Diamond }}\right) _{{\mathcal {D}}ominan\left( {\mathcal {T}}_{k}^{*},C\right) ^{+}}^{\beta \vee l\left( p\right) }\cap \sum \nolimits _{k=1}^{\left| D\right| }\left( \underline{P^{\Diamond }} \right) _{{\mathcal {D}}ominan\left( {\mathcal {T}}_{k}^{*},C\right) ^{+}}^{\beta \vee l\left( p\right) }\supseteq \sum \nolimits _{k=1}^{\left| D\right| }\left( \underline{S^{\Diamond }\cap P^{\Diamond }}\right) _{ {\mathcal {D}}ominan\left( {\mathcal {T}}_{k}^{*},C\right) ^{+}}^{\beta \vee l\left( p\right) },$$ for all $$S^{\Diamond },$$ $$Y^{\Diamond }\subseteq A;$$
10. $$\sum \nolimits _{k=1}^{\left| D\right| }\left( \overline{S^{\Diamond }}\right) _{{\mathcal {D}}ominan\left( {\mathcal {T}}_{k}^{*},C\right) ^{+}}^{\beta \vee l\left( p\right) }\cap \sum \nolimits _{k=1}^{\left| D\right| }\left( \overline{P^{\Diamond }} \right) _{{\mathcal {D}}ominan\left( {\mathcal {T}}_{k}^{*},C\right) ^{+}}^{\beta \vee l\left( p\right) }\supseteq \sum \nolimits _{k=1}^{\left| D\right| }\left( \overline{S^{\Diamond }\cap P^{\Diamond }}\right) _{ {\mathcal {D}}ominan\left( {\mathcal {T}}_{k}^{*},C\right) ^{+}}^{\beta \vee l\left( p\right) },$$ for all $$S^{\Diamond },$$ $$P^{\Diamond }\subseteq A.$$

#### Definition 12

Let ${\mathcal {I}}{\mathcal {S}}^{*}=\left( A,C,D,E\right)$ be an incomplete information system and $S^{\Diamond }\subseteq A,$ $\beta \in \left( 0.5,1 \right]$ and $l\in {\mathbb {N}} \cup \left\{ 0\right\} .$ Then the approximate precisions $\sum \nolimits _{k=1}^{\left| D\right| }\left( S^{\Diamond }\right) _{ {\mathcal {D}}ominan\left( {\mathcal {T}}_{k}^{*},C\right) ^{+}}^{\rho \left( \beta \vee l\right) \left( p\right) }$ and $\sum \nolimits _{k=1}^{\left| D\right| }\left( S^{\Diamond }\right) _{ {\mathcal {D}}ominan\left( {\mathcal {T}}_{k}^{*},C\right) ^{-}}^{\rho \left( \beta \vee l\right) \left( p\right) }$ of $S^{\Diamond }$ about ${\mathcal {D}}ominan\left( {\mathcal {T}}_{k}^{*},C\right) ^{+}$ and ${\mathcal {D}}ominan\left( {\mathcal {T}}_{k}^{*},C\right) ^{-}$ are defined by

$$\begin{aligned} \sum \limits _{k=1}^{\left| D\right| }\left( S^{\Diamond }\right) _{ {\mathcal {D}}ominan\left( {\mathcal {T}}_{k}^{*},C\right) ^{+}}^{\rho \left( \beta \vee l\right) \left( p\right) }=\frac{\left| \sum \limits _{k=1}^{\left| D\right| }\left( \underline{S^{\Diamond }} \right) _{{\mathcal {D}}ominan\left( {\mathcal {T}}_{k}^{*},C\right) ^{+}}^{\beta \vee l\left( p\right) }\right| }{\left| \sum \limits _{k=1}^{\left| D\right| }\left( \overline{S^{\Diamond }} \right) _{{\mathcal {D}}ominan\left( {\mathcal {T}}_{k}^{*},C\right) ^{+}}^{\beta \vee l\left( p\right) }\right| }; \end{aligned}$$

and

$$\begin{aligned} \ \sum \limits _{k=1}^{\left| D\right| }\left( S^{\Diamond }\right) _{{\mathcal {D}}ominan\left( {\mathcal {T}}_{k}^{*},C\right) ^{-}}^{\rho \left( \beta \vee l\right) \left( p\right) }=\frac{\left| \sum \limits _{k=1}^{\left| D\right| }\left( \underline{S^{\Diamond }} \right) _{{\mathcal {D}}ominan\left( {\mathcal {T}}_{k}^{*},C\right) ^{-}}^{\beta \vee l\left( p\right) }\right| }{\left| \sum \limits _{k=1}^{\left| D\right| }\left( \overline{S^{\Diamond }} \right) _{{\mathcal {D}}ominan\left( {\mathcal {T}}_{k}^{*},C\right) ^{-}}^{\beta \vee l\left( p\right) }\right| }; \end{aligned}$$

where $S^{\Diamond }\ne \emptyset ,$ and $\left| \cdot \right|$ denotes the cardinality of a set.

Let $\sum \nolimits _{k=1}^{\left| D\right| }\left( S^{\Diamond }\right) _{{\mathcal {D}}ominan\left( {\mathcal {T}}_{k}^{*},C\right) ^{+}}^{\mu \left( \beta \vee l\right) \left( p\right) }=1-\sum \nolimits _{k=1}^{\left| D\right| }\left( S^{\Diamond }\right) _{{\mathcal {D}}ominan\left( {\mathcal {T}}_{k}^{*},C\right) ^{+}}^{\rho \left( \beta \vee l\right) \left( p\right) }$ and $\sum \nolimits _{k=1}^{\left| D\right| }\left( S^{\Diamond }\right) _{ {\mathcal {D}}ominan\left( {\mathcal {T}}_{k}^{*},C\right) ^{-}}^{\mu \left( \beta \vee l\right) \left( p\right) }=1-\sum \nolimits _{k=1}^{\left| D\right| }\left( S^{\Diamond }\right) _{{\mathcal {D}}ominan\left( {\mathcal {T}}_{k}^{*},C\right) ^{-}}^{\rho \left( \beta \vee l\right) \left( p\right) }.$ Then $\sum \nolimits _{k=1}^{\left| D\right| }\left( S^{\Diamond }\right) _{{\mathcal {D}}ominan\left( {\mathcal {T}} _{k}^{*},C\right) ^{+}}^{\mu \left( \beta \vee l\right) \left( p\right) }$ and $\sum \nolimits _{k=1}^{\left| D\right| }\left( S^{\Diamond }\right) _{{\mathcal {D}}ominan\left( {\mathcal {T}}_{k}^{*},C\right) ^{-}}^{\mu \left( \beta \vee l\right) \left( p\right) }$ are called the rough degrees of $S^{\Diamond }$ about ${\mathcal {D}}ominan\left( {\mathcal {T}}_{k}^{*},C\right) ^{+}$ and ${\mathcal {D}}ominan\left( {\mathcal {T}}_{k}^{*},C\right) ^{-}$ respectively.

By definition $0\le \sum \nolimits _{k=1}^{\left| D\right| }\left( S^{\Diamond }\right) _{{\mathcal {D}}ominan\left( {\mathcal {T}} _{k}^{*},C\right) ^{+}}^{\mu \left( \beta \vee l\right) \left( p\right) }\le 1$ and $0\le \sum \nolimits _{k=1}^{\left| D\right| }\left( S^{\Diamond }\right) _{{\mathcal {D}}ominan\left( {\mathcal {T}} _{k}^{*},C\right) ^{-}}^{\mu \left( \beta \vee l\right) \left( p\right) }\le 1.$

#### Definition 13

Let ${\mathcal {I}}{\mathcal {S}}^{*}=\left( A,C,D,E\right)$ be an incomplete information system and $S^{\Diamond }\subseteq A,$ $\beta \in \left( 0.5,1 \right]$ and $l\in {\mathbb {N}} \cup \left\{ 0\right\} .$ Then the *approximate quality* $\sum \nolimits _{k=1}^{\left| D\right| }\left( S^{\Diamond }\right) _{ {\mathcal {D}}ominan\left( {\mathcal {T}}_{k}^{*},C\right) ^{+}}^{\gamma \left( \beta \vee l\right) \left( p\right) }$ and $\sum \nolimits _{k=1}^{\left| D\right| }\left( S^{\Diamond }\right) _{ {\mathcal {D}}ominan\left( {\mathcal {T}}_{k}^{*},C\right) ^{-}}^{\gamma \left( \beta \vee l\right) \left( p\right) }$ of $S^{\Diamond }$ about ${\mathcal {D}}ominan\left( {\mathcal {T}}_{k}^{*},C\right) ^{+}$ and ${\mathcal {D}}ominan\left( {\mathcal {T}}_{k}^{*},C\right) ^{-}$ are defined by

$$\begin{aligned} \sum \limits _{k=1}^{\left| D\right| }\left( S^{\Diamond }\right) _{ {\mathcal {D}}ominan\left( {\mathcal {T}}_{k}^{*},C\right) ^{+}}^{\gamma \left( \beta \vee l\right) \left( p\right) }=\frac{\left| \sum \limits _{k=1}^{\left| D\right| }\left( \underline{S^{\Diamond }} \right) _{{\mathcal {D}}ominan\left( {\mathcal {T}}_{k}^{*},C\right) ^{+}}^{\beta \vee l\left( p\right) }\right| }{\left| A\right| } \end{aligned}$$

and

$$\begin{aligned} \sum \limits _{k=1}^{\left| D\right| }\left( S^{\Diamond }\right) _{ {\mathcal {D}}ominan\left( {\mathcal {T}}_{k}^{*},C\right) ^{-}}^{\gamma \left( \beta \vee l\right) \left( p\right) }=\frac{\left| \sum \limits _{k=1}^{\left| D\right| }\left( \underline{S^{\Diamond }} \right) _{{\mathcal {D}}ominan\left( {\mathcal {T}}_{k}^{*},C\right) ^{-}}^{\beta \vee l\left( p\right) }\right| }{\left| A\right| }. \end{aligned}$$

Clearly $$0\le \sum \nolimits _{k=1}^{\left| D\right| }\left( S^{\Diamond }\right) _{{\mathcal {D}}ominan\left( {\mathcal {T}} _{k}^{*},C\right) ^{+}}^{\gamma \left( \beta \vee l\right) \left( p\right) }\le 1$$ and $$0\le \sum \nolimits _{k=1}^{\left| D\right| }\left( S^{\Diamond }\right) _{{\mathcal {D}}ominan\left( {\mathcal {T}} _{k}^{*},C\right) ^{-}}^{\gamma \left( \beta \vee l\right) \left( p\right) }\le 1.$$

We now obtain three way decision rules using logical disjunction soft dominating pessimistic multigranulation rough sets:

$\left( \mathrm{i}\right)$ If there exists $k\in \left\{ 1,2,3,...,\left| D\right| \right\}$ such that $\left( \frac{ \left| \left[ b\right] _{{\mathcal {D}}ominan\left( {\mathcal {T}} _{k}^{*},C\right) }^{+}\cap S^{\Diamond }\right| }{\left| \left[ b\right] _{{\mathcal {D}}ominan\left( {\mathcal {T}}_{k}^{*},C\right) }^{+}\right| }\right) >\beta \vee \left( \left| \left[ b\right] _{ {\mathcal {D}}ominan\left( {\mathcal {T}}_{k}^{*},C\right) }^{+}\right| -\left| \left[ b\right] _{{\mathcal {D}}ominan\left( {\mathcal {T}}_{k}^{*},C\right) }^{+}\cap S^{\Diamond }\right| \right) <l,$ decide $Pos\left( S^{\Diamond }\right) _{{\mathcal {D}}ominan \left( {\mathcal {T}}_{k}^{*},C\right) ^{+}}^{\beta \vee l\left( p\right) };$

$\left( \mathrm{ii}\right)$ If there exists for all $k\in \left\{ 1,2,3,...,\left| D\right| \right\}$ such that $\left( \frac{ \left| \left[ b\right] _{{\mathcal {D}}ominan\left( {\mathcal {T}} _{k}^{*},C\right) }^{+}\cap S^{\Diamond }\right| }{\left| \left[ b\right] _{{\mathcal {D}}ominan\left( {\mathcal {T}}_{k}^{*},C\right) }^{+}\right| }\right)<1-\beta \vee \left( \left| \left[ b\right] _{ {\mathcal {D}}ominan\left( {\mathcal {T}}_{k}^{*},C\right) }^{+}\right| -\left| \left[ b\right] _{{\mathcal {D}}ominan\left( {\mathcal {T}}_{k}^{*},C\right) }^{+}\cap S^{\Diamond }\right| \right) <l,$ decide $Neg\left( S^{\Diamond }\right) _{{\mathcal {D}}ominan \left( {\mathcal {T}}_{k}^{*},C\right) ^{+}}^{\beta \vee l\left( p\right) };$

$$\left( \mathrm{iii}\right) $$ Otherwise, decide $$\sum \nolimits _{k=1}^{\left| D\right| }\left( S^{\Diamond }\right) _{{\mathcal {D}}ominan \left( {\mathcal {T}}_{k}^{*},C\right) ^{+}}^{\beta \vee l\left( p\right) }.$$

Similarly, we now obtain three way decision rules using logical disjunction soft dominated pessimistic multigranulation rough sets:

$\left( \mathrm{i}\right)$ If there exists $k\in \left\{ 1,2,3,...,\left| D\right| \right\}$ such that $\left( \frac{ \left| \left[ b\right] _{{\mathcal {D}}ominan\left( {\mathcal {T}} _{k}^{*},C\right) }^{-}\cap S^{\Diamond }\right| }{\left| \left[ b\right] _{{\mathcal {D}}ominan\left( {\mathcal {T}}_{k}^{*},C\right) }^{-}\right| }\right) >\beta \vee \left( \left| \left[ b\right] _{ {\mathcal {D}}ominan\left( {\mathcal {T}}_{k}^{*},C\right) }^{-}\right| -\left| \left[ b\right] _{{\mathcal {D}}ominan\left( {\mathcal {T}}_{k}^{*},C\right) }^{-}\cap S^{\Diamond }\right| \right) <l,$ decide $Pos\left( S^{\Diamond }\right) _{{\mathcal {D}}ominan \left( {\mathcal {T}}_{k}^{*},C\right) ^{-}}^{\beta \vee l\left( p\right) };$

$\left( \mathrm{ii}\right)$ If there exists for all $k\in \left\{ 1,2,3,...,\left| D\right| \right\}$ such that $\left( \frac{ \left| \left[ b\right] _{{\mathcal {D}}ominan\left( {\mathcal {T}} _{k}^{*},C\right) }^{-}\cap S^{\Diamond }\right| }{\left| \left[ b\right] _{{\mathcal {D}}ominan\left( {\mathcal {T}}_{k}^{*},C\right) }^{-}\right| }\right)<1-\beta \vee \left( \left| \left[ b\right] _{ {\mathcal {D}}ominan\left( {\mathcal {T}}_{k}^{*},C\right) }^{-}\right| -\left| \left[ b\right] _{{\mathcal {D}}ominan\left( {\mathcal {T}}_{k}^{*},C\right) }^{-}\cap S^{\Diamond }\right| \right) <l,$ decide $Neg\left( S^{\Diamond }\right) _{{\mathcal {D}}ominan \left( {\mathcal {T}}_{k}^{*},C\right) ^{-}}^{\beta \vee l\left( p\right) };$

$$\left( \mathrm{iii}\right) $$ Otherwise, decide $$\sum \nolimits _{k=1}^{\left| D\right| }\left( S^{\Diamond }\right) _{{\mathcal {D}}ominan\left( {\mathcal {T}}_{k}^{*},C\right) ^{-}}^{\beta \vee l\left( p\right) }.$$

### Proposed Agorithm-$\mathrm{III}$ for conflict analysis model

**Input:** Given information system ${\mathcal {I}}{\mathcal {S}}^{*}=\left( A,C,D,E\right) ;$

**Step** 1: Construct $\left( {\mathcal {T}}_{k}^{*},C\right)$ corresponding to the $D_{k}$ for all $k=1,2,...,\left| D\right|$;

**Step** 2: Construct ${\mathcal {D}}ominan\left( {\mathcal {T}} _{k}^{*},C\right) ,$ for all $k=1,2,...,\left| D\right|$;

**Step** 3: Construct $\sum \nolimits _{k=1}^{\left| D\right| }\left( \underline{S_{k}^{\Diamond }}\right) _{{\mathcal {D}}ominan \left( {\mathcal {T}}_{k}^{*},C\right) ^{+}}^{\beta \vee l\left( p\right) }$ and $\sum \nolimits _{k=1}^{\left| D\right| }\left( \underline{ S_{k}^{\Diamond }}\right) _{{\mathcal {D}}ominan\left( {\mathcal {T}} _{k}^{*},C\right) ^{-}}^{\beta \vee l\left( p\right) }$, where $\beta \in \left( 0.5,1\right]$, $l\in {\mathbb {N}} \cup \left\{ 0\right\}$ and $S^{\Diamond }=\left\{ b\in A:g(b,D_{k})\ne 0\right\} ;$

**Step** 4: If $\sum \nolimits _{k=1}^{\left| D\right| }\left( \underline{S_{k}^{\Diamond }}\right) _{{\mathcal {D}}ominan\left( {\mathcal {T}}_{k}^{*},C\right) ^{+}}^{\beta \vee l\left( p\right) }=\sum \nolimits _{k=1}^{\left| D\right| }\left( \underline{ S_{k}^{\Diamond }}\right) _{{\mathcal {D}}ominan\left( {\mathcal {T}} _{k}^{*},C\right) ^{-}}^{\beta \vee l\left( p\right) }$ then $\delta _{k}=\sum \nolimits _{k=1}^{\left| D\right| }\left( \underline{ S_{k}^{\Diamond }}\right) _{{\mathcal {D}}ominan\left( {\mathcal {T}} _{k}^{*},C\right) ^{+}}^{\beta \vee l\left( p\right) }$ or $\delta _{k}=\sum \nolimits _{k=1}^{\left| D\right| }\left( \underline{ S_{k}^{\Diamond }}\right) _{{\mathcal {D}}ominan\left( {\mathcal {T}} _{k}^{*},C\right) ^{-}}^{\beta \vee l\left( p\right) }$ otherwise $\delta _{k}=\sum \nolimits _{k=1}^{\left| D\right| }\left( \underline{ S_{k}^{\Diamond }}\right) _{{\mathcal {D}}ominan\left( {\mathcal {T}} _{k}^{*},C\right) ^{+}}^{\beta \vee l\left( p\right) }-\sum \nolimits _{k=1}^{\left| D\right| }\left( \underline{ S_{k}^{\Diamond }}\right) _{{\mathcal {D}}ominan\left( {\mathcal {T}} _{k}^{*},C\right) ^{-}}^{\beta \vee l\left( p\right) };$

**Step** 5: $$\delta =\bigcap \nolimits _{k=1}^{\left| D\right| }\delta _{k};$$

**Step** 6: If $$\delta \ne \emptyset ;$$

         go to **output.**

         otherwise,

         put $\beta ^{\prime }$ in **Step** 3, where $\beta ^{\prime }\le \beta ,$

or

         put $l^{\prime }$ in **Step** 3, where $l\le l^{\prime };$

**Output:** Feasible alternative(s) for feasible consensus strategy.

#### Example 4

(Continued from Example 1) Let ${\mathcal {I}}{\mathcal {S}}^{*}=\left( A,C,D,E\right)$ be an incomplete information system. Applying the Algorithm-$\mathrm{III}$, we have

From Tables 10 and 11 one get $\delta :=\left\{ b_{7}\right\} .$ The obtained result shows that $b_{7}$ is the optimal decision medicine with decision precision 0.9.

**Table 10:** The lower approximations with $\beta =0.90, l=1$.

$S^{\Diamond }$	$\sum \limits _{k=1}^{\left| D\right| }\left( \underline{S_{k}^{\Diamond }}\right) _{{\mathcal {D}}ominan\left( {\mathcal {T}}_{k}^{*},C\right) ^{+}}^{\beta \vee l\left( p\right) }$	$\sum \limits _{k=1}^{\left| D\right| }\left( \underline{S_{k}^{\Diamond }}\right) _{{\mathcal {D}}om inan\left( {\mathcal {T}}_{k}^{*},C\right) ^{-}}^{\beta \vee l\left( p\right) }$
$S_{1}^{\Diamond }$	$\left\{ b_{2},b_{6},b_{7},b_{9},b_{12}\right\}$	$\left\{ b_{1},b_{4}\right\}$
$S_{2}^{\Diamond }$	$\left\{ b_{7},b_{8},b_{9},b_{10},b_{11},b_{12}\right\}$	$\left\{ b_{1},b_{2},b_{4},b_{5},b_{8},b_{9},b_{10},b_{11},b_{12}\right\}$
$S_{3}^{\Diamond }$	$\left\{ b_{6},b_{7},b_{9}\right\}$	$\left\{ b_{4}\right\}$
$S_{4}^{\Diamond }$	$\left\{ b_{7},b_{8},b_{9},b_{12}\right\}$	$\left\{ b_{1},b_{4},b_{5},b_{10}\right\}$

**Table 11:** Decision table with $\beta =0.90, l=1$.

$\delta _{k}$	$\sum \limits _{k=1}^{\left| D\right| }\left( \underline{S_{k}^{\Diamond }}\right) _{{\mathcal {D}} ominan\left( {\mathcal {T}}_{k}^{*},C\right) ^{+}}^{\beta \vee l\left( p\right) }-\sum \limits _{k=1}^{\left| D\right| }\left( \underline{S_{k}^{\Diamond }}\right) _{{\mathcal {D}}ominan \left( {\mathcal {T}}_{k}^{*},C\right) ^{-}}^{\beta \vee l\left( p\right) }$
$\delta _{1}$	$\left\{ b_{2},b_{6},b_{7},b_{9},b_{12}\right\}$
$\delta _{2}$	$\left\{ b_{7}\right\}$
$\delta _{3}$	$\left\{ b_{6},b_{7},b_{9}\right\}$
$\delta _{4}$	$\left\{ b_{7},b_{8},b_{9},b_{12}\right\}$

### Logical conjunction soft dominating/dominated pessimistic multigranulation rough sets

#### Definition 14

Let ${\mathcal {I}}{\mathcal {S}}^{*}=\left( A,C,D,E\right)$ be an incomplete information system and $S^{\Diamond }\subseteq A.$ If $\beta \in \left( 0.5,1\right]$ and $l\in {\mathbb {N}} \cup \left\{ 0\right\} ,$ then the pessimistic lower approximation and pessimistic upper approximation of $S^{\Diamond }$ with respect to ${\mathcal {D}}ominan\left( {\mathcal {T}}_{k}^{*},C\right) ^{+}$ are defined by

$$\begin{aligned} \sum \limits _{k=1}^{\left| D\right| }\left( \underline{S^{\Diamond }} \right) _{{\mathcal {D}}ominan\left( {\mathcal {T}}_{k}^{*},C\right) ^{+}}^{\beta \wedge l\left( p\right) } =\bigwedge \limits _{k=1}^{\left| D\right| }\left\{ \begin{array}{c} b\in A\mid \left( \frac{\left| \left[ b\right] _{{\mathcal {D}}ominan \left( {\mathcal {T}}_{k}^{*},C\right) }^{+}\cap S^{\Diamond }\right| }{\left| \left[ b\right] _{{\mathcal {D}}ominan\left( {\mathcal {T}} _{k}^{*},C\right) }^{+}\right| }\right) >\beta \\ \wedge \left( \left| \left[ b\right] _{{\mathcal {D}}ominan\left( {\mathcal {T}}_{k}^{*},C\right) }^{+}\right| -\left| \left[ b\right] _{{\mathcal {D}}ominan\left( {\mathcal {T}}_{k}^{*},C\right) }^{+}\cap S^{\Diamond }\right| \right) <l \end{array}\right\} ; \end{aligned}$$

and

$$\begin{aligned} \sum \limits _{k=1}^{\left| D\right| }\left( \overline{S^{\Diamond }} \right) _{{\mathcal {D}}ominan\left( {\mathcal {T}}_{k}^{*},C\right) ^{+}}^{\beta \wedge l\left( p\right) } =\bigvee \limits _{k=1}^{\left| D\right| }\left\{ \begin{array}{c} b\in A\mid \left( \frac{\left| \left[ b\right] _{{\mathcal {D}}ominan \left( {\mathcal {T}}_{k}^{*},C\right) }^{+}\cap S^{\Diamond }\right| }{\left| \left[ b\right] _{{\mathcal {D}}ominan\left( {\mathcal {T}} _{k}^{*},C\right) }^{+}\right| }\right) \ge 1-\beta \\ \wedge \left( \left| \left[ b\right] _{{\mathcal {D}}ominan\left( {\mathcal {T}}_{k}^{*},C\right) }^{+}\cap S^{\Diamond }\right| \right) \ge l \end{array}\right\} . \end{aligned}$$

The pair $\left( \sum \nolimits _{k=1}^{\left| D\right| }\left( \underline{S^{\Diamond }}\right) _{{\mathcal {D}}ominan\left( {\mathcal {T}}_{k}^{*},C\right) ^{+}}^{\beta \wedge l\left( p\right) },\sum \nolimits _{k=1}^{\left| D\right| }\left( \overline{S^{\Diamond }} \right) _{{\mathcal {D}}ominan\left( {\mathcal {T}}_{k}^{*},C\right) ^{+}}^{\beta \wedge l\left( p\right) }\right)$ is called the logical conjunction soft dominating pessimistic multigranulation rough set if $\sum \nolimits _{k=1}^{\left| D\right| }\left( \underline{S^{\Diamond }} \right) _{{\mathcal {D}}ominan\left( {\mathcal {T}}_{k}^{*},C\right) ^{+}}^{\beta \wedge l\left( o\right) }\ne \sum \nolimits _{k=1}^{\left| D\right| }\left( \overline{S^{\Diamond }}\right) _{{\mathcal {D}}ominan \left( {\mathcal {T}}_{k}^{*},C\right) ^{+}}^{\beta \wedge l\left( o\right) }.$ Similarly the pessimistic lower approximation and pessimistic upper approximation of $S^{\Diamond }$ with respect to ${\mathcal {D}}ominan\left( {\mathcal {T}}_{k}^{*},C\right) ^{-}$ are defined by

$$\begin{aligned} \sum \limits _{k=1}^{\left| D\right| }\left( \underline{S^{\Diamond }} \right) _{{\mathcal {D}}ominan\left( {\mathcal {T}}_{k}^{*},C\right) ^{-}}^{\beta \wedge l\left( p\right) }=\bigwedge \limits _{k=1}^{\left| D\right| }\left\{ \begin{array}{c} b\in A\mid \left( \frac{\left| \left[ b\right] _{{\mathcal {D}}ominan \left( {\mathcal {T}}_{k}^{*},C\right) }^{-}\cap S^{\Diamond }\right| }{\left| \left[ b\right] _{{\mathcal {D}}ominan\left( {\mathcal {T}} _{k}^{*},C\right) }^{-}\right| }\right) >\beta \\ \wedge \left( \left| \left[ b\right] _{{\mathcal {D}}ominan\left( {\mathcal {T}}_{k}^{*},C\right) }^{-}\right| -\left| \left[ b\right] _{{\mathcal {D}}ominan\left( {\mathcal {T}}_{k}^{*},C\right) }^{-}\cap S^{\Diamond }\right| \right) <l \end{array}\right\} ; \end{aligned}$$

and

$$\begin{aligned} \sum \limits _{k=1}^{\left| D\right| }\left( \overline{S^{\Diamond }} \right) _{{\mathcal {D}}ominan\left( {\mathcal {T}}_{k}^{*},C\right) ^{-}}^{\beta \wedge l\left( p\right) }=\bigvee \limits _{k=1}^{\left| D\right| }\left\{ \begin{array}{c} b\in A\mid \left( \frac{\left| \left[ b\right] _{{\mathcal {D}}ominan \left( {\mathcal {T}}_{k}^{*},C\right) }^{-}\cap S^{\Diamond }\right| }{\left| \left[ b\right] _{{\mathcal {D}}ominan\left( {\mathcal {T}} _{k}^{*},C\right) }^{-}\right| }\right) \ge 1-\beta \\ \wedge \left( \left| \left[ b\right] _{{\mathcal {D}}ominan\left( {\mathcal {T}}_{k}^{*},C\right) }^{-}\cap S^{\Diamond }\right| \right) \ge l \end{array}\right\} . \end{aligned}$$

The pair $\left( \sum \nolimits _{k=1}^{\left| D\right| }\left( \underline{S^{\Diamond }}\right) _{{\mathcal {D}}ominan\left( {\mathcal {T}}_{k}^{*},C\right) ^{-}}^{\beta \wedge l\left( p\right) },\sum \nolimits _{k=1}^{\left| D\right| }\left( \overline{S^{\Diamond }} \right) _{{\mathcal {D}}ominan\left( {\mathcal {T}}_{k}^{*},C\right) ^{-}}^{\beta \wedge l\left( p\right) }\right)$ is called the logical conjunction soft dominated pessimistic multigranulation rough set if $\sum \nolimits _{k=1}^{\left| D\right| }\left( \underline{S^{\Diamond }} \right) _{{\mathcal {D}}ominan\left( {\mathcal {T}}_{k}^{*},C\right) ^{-}}^{\beta \wedge l\left( p\right) }\ne \sum \nolimits _{k=1}^{\left| D\right| }\left( \overline{S^{\Diamond }}\right) _{{\mathcal {D}}ominan \left( {\mathcal {T}}_{k}^{*},C\right) ^{-}}^{\beta \wedge l\left( p\right) }.$ Using the lower approximation $\sum \nolimits _{k=1}^{\left| D\right| }\left( \underline{X^{\Diamond }}\right) _{{\mathcal {D}}ominan \left( {\mathcal {T}}_{k}^{*},C\right) ^{+}}^{\beta \wedge l\left( p\right) }$ and upper approximation $\sum \nolimits _{k=1}^{\left| D\right| }\left( \overline{S^{\Diamond }}\right) _{{\mathcal {D}}ominan \left( {\mathcal {T}}_{k}^{*},C\right) ^{+}}^{\beta \wedge l\left( p\right) },$ the logical conjunction soft dominating pessimistic multigranulation boundary region of $S^{\Diamond }$ is

$$\begin{aligned} \sum \limits _{k=1}^{\left| D\right| }\left( S^{\Diamond }\right) _{ {\mathcal {D}}ominan\left( {\mathcal {T}}_{k}^{*},C\right) ^{+}}^{\beta \wedge l\left( p\right) }=\sum \limits _{k=1}^{\left| D\right| }\left( \overline{S^{\Diamond }}\right) _{{\mathcal {D}}ominan \left( {\mathcal {T}}_{k}^{*},C\right) ^{+}}^{\beta \wedge l\left( p\right) }-\sum \limits _{k=1}^{\left| D\right| }\left( \underline{ X^{\Diamond }}\right) _{{\mathcal {D}}ominan\left( {\mathcal {T}} _{k}^{*},C\right) ^{+}}^{\beta \wedge l\left( p\right) }. \end{aligned}$$

Similarly the lower approximation $\sum \nolimits _{k=1}^{\left| D\right| }\left( \underline{S^{\Diamond }}\right) _{{\mathcal {D}}ominan\left( {\mathcal {T}}_{k}^{*},C\right) ^{-}}^{\beta \wedge l\left( p\right) }$ and upper approximation $\sum \nolimits _{k=1}^{\left| D\right| }\left( \overline{S^{\Diamond }}\right) _{{\mathcal {D}}ominan \left( {\mathcal {T}}_{k}^{*},C\right) ^{-}}^{\beta \wedge l\left( p\right) },$ the logical conjunction soft dominated pessimistic multigranulation boundary region of $S^{\Diamond }$ is

$$\begin{aligned} \sum \limits _{k=1}^{\left| D\right| }\left( S^{\Diamond }\right) _{ {\mathcal {D}}ominan\left( {\mathcal {T}}_{k}^{*},C\right) ^{-}}^{\beta \wedge l\left( p\right) }=\sum \limits _{k=1}^{\left| D\right| }\left( \overline{S^{\Diamond }}\right) _{{\mathcal {D}}ominan \left( {\mathcal {T}}_{k}^{*},C\right) ^{-}}^{\beta \wedge l\left( p\right) }-\sum \limits _{k=1}^{\left| D\right| }\left( \underline{ S^{\Diamond }}\right) _{{\mathcal {D}}ominan\left( {\mathcal {T}} _{k}^{*},C\right) ^{-}}^{\beta \wedge l\left( p\right) }. \end{aligned}$$

#### Theorem 8

Let ${\mathcal {I}}{\mathcal {S}}^{*}=\left( A,C,D,E\right)$ be an incomplete information system and $S^{\Diamond }\subseteq A.$ If $\beta \in \left( 0.5,1 \right]$ and $l\in {\mathbb {N}} \cup \left\{ 0\right\} ,$ then the following hold:

1. $$\sum \nolimits _{k=1}^{\left| D\right| }\left( \underline{S^{\Diamond }}\right) _{{\mathcal {D}}ominan\left( {\mathcal {T}}_{k}^{*},C\right) ^{+}}^{\beta \wedge l\left( p\right) }=\sum \nolimits _{k=1}^{\left| D\right| }\left( \underline{S^{\Diamond }}\right) _{{\mathcal {D}}ominan\left( {\mathcal {T}}_{k}^{*},C\right) ^{+}}^{\beta \left( p\right) }\cap \sum \nolimits _{k=1}^{\left| D\right| }\left( \underline{S^{\Diamond }}\right) _{{\mathcal {D}}ominan \left( {\mathcal {T}}_{k}^{*},C\right) ^{+}}^{l\left( p\right) };$$
2. $$\sum \nolimits _{k=1}^{\left| D\right| }\left( \overline{S^{\Diamond }}\right) _{{\mathcal {D}}ominan\left( {\mathcal {T}}_{k}^{*},C\right) ^{+}}^{\beta \wedge l\left( p\right) }=\sum \nolimits _{k=1}^{\left| D\right| }\left( \overline{S^{\Diamond }} \right) _{{\mathcal {D}}ominan\left( {\mathcal {T}}_{k}^{*},C\right) ^{+}}^{\beta \left( p\right) }\cap \sum \nolimits _{k=1}^{\left| D\right| }\left( \overline{S^{\Diamond }}\right) _{{\mathcal {D}}ominan \left( {\mathcal {T}}_{k}^{*},C\right) ^{+}}^{l\left( p\right) };$$
3. $$\sum \nolimits _{k=1}^{\left| D\right| }\left( \underline{A}\right) _{{\mathcal {D}}ominan\left( {\mathcal {T}} _{k}^{*},C\right) ^{+}}^{\beta \wedge l\left( p\right) }=\sum \nolimits _{k=1}^{\left| D\right| }\left( \overline{A}\right) _{ {\mathcal {D}}ominan\left( {\mathcal {T}}_{k}^{*},C\right) ^{+}}^{\beta \wedge l\left( p\right) }=A;$$
4. $$\sum \nolimits _{k=1}^{\left| D\right| }\left( \underline{\emptyset }\right) _{{\mathcal {D}}ominan\left( {\mathcal {T}} _{k}^{*},C\right) ^{+}}^{\beta \wedge l\left( p\right) }=\sum \nolimits _{k=1}^{\left| D\right| }\left( \overline{\emptyset } \right) _{{\mathcal {D}}ominan\left( {\mathcal {T}}_{k}^{*},C\right) ^{+}}^{\beta \wedge l\left( p\right) }=\emptyset ;$$
5. $$\sum \nolimits _{k=1}^{\left| D\right| }\left( \underline{S^{\Diamond }}\right) _{{\mathcal {D}}ominan\left( {\mathcal {T}}_{k}^{*},C\right) ^{+}}^{\beta \wedge l_{1}\left( p\right) }\subseteq \sum \nolimits _{k=1}^{\left| D\right| }\left( \underline{S^{\Diamond }} \right) _{{\mathcal {D}}ominan\left( {\mathcal {T}}_{k}^{*},C\right) ^{+}}^{\beta \wedge l_{2}\left( p\right) },$$ $$\sum \nolimits _{k=1}^{\left| D\right| }\left( \overline{S^{\Diamond }}\right) _{{\mathcal {D}}ominan \left( {\mathcal {T}}_{k}^{*},C\right) ^{+}}^{\beta \wedge l_{2}\left( p\right) }\subseteq \sum \nolimits _{k=1}^{\left| D\right| }\left( \overline{S^{\Diamond }}\right) _{{\mathcal {D}}ominan\left( {\mathcal {T}}_{k}^{*},C\right) ^{+}}^{\beta \wedge l_{1}\left( p\right) },$$ if $$ l_{1}<l_{2}\in {\mathbb {N}} \cup \left\{ 0\right\} ;$$
6. $$\sum \nolimits _{k=1}^{\left| D\right| }\left( \underline{S^{\Diamond }}\right) _{{\mathcal {D}}ominan\left( {\mathcal {T}}_{k}^{*},C\right) ^{+}}^{\beta _{2}\wedge l\left( p\right) }\subseteq \sum \nolimits _{k=1}^{\left| D\right| }\left( \underline{S^{\Diamond }} \right) _{{\mathcal {D}}ominan\left( {\mathcal {T}}_{k}^{*},C\right) ^{+}}^{\beta _{1}\wedge l\left( p\right) },$$ $$\sum \nolimits _{k=1}^{\left| D\right| }\left( \overline{S^{\Diamond }}\right) _{{\mathcal {D}}ominan \left( {\mathcal {T}}_{k}^{*},C\right) ^{+}}^{\beta _{1}\wedge l\left( p\right) }\subseteq \sum \nolimits _{k=1}^{\left| D\right| }\left( \overline{S^{\Diamond }}\right) _{{\mathcal {D}}ominan\left( {\mathcal {T}}_{k}^{*},C\right) ^{+}}^{\beta _{2}\wedge l\left( p\right) },$$ if $$ \beta _{1}\le \beta _{2}\in \left( 0.5,1\right] ;$$
7. $$\sum \nolimits _{k=1}^{\left| D\right| }\left( \underline{S^{\Diamond }}\right) _{{\mathcal {D}}ominan\left( {\mathcal {T}}_{k}^{*},C\right) ^{+}}^{\beta \wedge l\left( p\right) }\cup \sum \nolimits _{k=1}^{\left| D\right| }\left( \underline{P^{\Diamond }} \right) _{{\mathcal {D}}ominan\left( {\mathcal {T}}_{k}^{*},C\right) ^{+}}^{\beta \wedge l\left( p\right) }\subseteq \sum \nolimits _{k=1}^{\left| D\right| }\left( \underline{S^{\Diamond }\cup P^{\Diamond }}\right) _{{\mathcal {D}}ominan\left( {\mathcal {T}} _{k}^{*},C\right) ^{+}}^{\beta \wedge l\left( p\right) },$$ for all $$ S^{\Diamond },$$ $$P^{\Diamond }\subseteq A;$$
8. $$\sum \nolimits _{k=1}^{\left| D\right| }\left( \overline{S^{\Diamond }}\right) _{{\mathcal {D}}ominan\left( {\mathcal {T}}_{k}^{*},C\right) ^{+}}^{\beta \wedge l\left( p\right) }\cup \sum \nolimits _{k=1}^{\left| D\right| }\left( \overline{P^{\Diamond }} \right) _{{\mathcal {D}}ominan\left( {\mathcal {T}}_{k}^{*},C\right) ^{+}}^{\beta \wedge l\left( p\right) }\subseteq \sum \nolimits _{k=1}^{\left| D\right| }\left( \overline{S^{\Diamond }\cup P^{\Diamond }}\right) _{{\mathcal {D}}ominan\left( {\mathcal {T}} _{k}^{*},C\right) ^{+}}^{\beta \wedge l\left( p\right) },$$ for all $$ S^{\Diamond },$$ $$P^{\Diamond }\subseteq A;$$
9. $$\sum \nolimits _{k=1}^{\left| D\right| }\left( \underline{S^{\Diamond }}\right) _{{\mathcal {D}}ominan\left( {\mathcal {T}}_{k}^{*},C\right) ^{+}}^{\beta \wedge l\left( p\right) }\cap \sum \nolimits _{k=1}^{\left| D\right| }\left( \underline{P^{\Diamond }} \right) _{{\mathcal {D}}ominan\left( {\mathcal {T}}_{k}^{*},C\right) ^{+}}^{\beta \wedge l\left( p\right) }\supseteq \sum \nolimits _{k=1}^{\left| D\right| }\left( \underline{S^{\Diamond }\cap P^{\Diamond }}\right) _{{\mathcal {D}}ominan\left( {\mathcal {T}} _{k}^{*},C\right) ^{+}}^{\beta \wedge l\left( p\right) },$$ for all $$ S^{\Diamond },$$ $$P^{\Diamond }\subseteq A;$$
10. $$\sum \nolimits _{k=1}^{\left| D\right| }\left( \overline{S^{\Diamond }}\right) _{{\mathcal {D}}ominan\left( {\mathcal {T}}_{k}^{*},C\right) ^{+}}^{\beta \wedge l\left( p\right) }\cap \sum \nolimits _{k=1}^{\left| D\right| }\left( \overline{P^{\Diamond }} \right) _{{\mathcal {D}}ominan\left( {\mathcal {T}}_{k}^{*},C\right) ^{+}}^{\beta \wedge l\left( p\right) }\supseteq \sum \nolimits _{k=1}^{\left| D\right| }\left( \overline{S^{\Diamond }\cap P^{\Diamond }}\right) _{{\mathcal {D}}ominan\left( {\mathcal {T}} _{k}^{*},C\right) ^{+}}^{\beta \wedge l\left( p\right) },$$ for all $$ S^{\Diamond },$$ $$P^{\Diamond }\subseteq A.$$

#### Definition 15

Let ${\mathcal {I}}{\mathcal {S}}^{*}=\left( A,C,D,E\right)$ be an incomplete information system and $S^{\Diamond }\subseteq A,$ $\beta \in \left( 0.5,1 \right]$ and $l\in {\mathbb {N}} \cup \left\{ 0\right\} .$ Then the approximate precisions $\sum \nolimits _{k=1}^{\left| D\right| }\left( S^{\Diamond }\right) _{ {\mathcal {D}}ominan\left( {\mathcal {T}}_{k}^{*},C\right) ^{+}}^{\rho \left( \beta \wedge l\right) \left( p\right) }$ and $\sum \nolimits _{k=1}^{\left| D\right| }\left( S^{\Diamond }\right) _{ {\mathcal {D}}ominan\left( {\mathcal {T}}_{k}^{*}, C\right) ^{-}}^{\rho \left( \beta \wedge l\right) \left( p\right) }$ of $S^{\Diamond }$ about ${\mathcal {D}}ominan\left( {\mathcal {T}}_{k}^{*},C\right) ^{+}$ and ${\mathcal {D}}ominan\left( {\mathcal {T}}_{k}^{*},C\right) ^{-}$ are defined by

$$\begin{aligned} \sum \limits _{k=1}^{\left| D\right| }\left( S^{\Diamond }\right) _{ {\mathcal {D}}ominan\left( {\mathcal {T}}_{k}^{*},C\right) ^{+}}^{\rho \left( \beta \wedge l\right) \left( p\right) }=\frac{\left| \sum \limits _{k=1}^{\left| D\right| }\left( \underline{S^{\Diamond }} \right) _{{\mathcal {D}}ominan\left( {\mathcal {T}}_{k}^{*},C\right) ^{+}}^{\beta \wedge l\left( p\right) }\right| }{\left| \sum \limits _{k=1}^{\left| D\right| }\left( \overline{S^{\Diamond }} \right) _{{\mathcal {D}}ominan\left( {\mathcal {T}}_{k}^{*},C\right) ^{+}}^{\beta \wedge l\left( p\right) }\right| } \end{aligned}$$

and

$$\begin{aligned} \sum \limits _{k=1}^{\left| D\right| }\left( S^{\Diamond }\right) _{{\mathcal {D}}ominan\left( {\mathcal {T}}_{k}^{*},C\right) ^{-}}^{\rho \left( \beta \wedge l\right) \left( p\right) }=\frac{\left| \sum \limits _{k=1}^{\left| D\right| }\left( \underline{S^{\Diamond }} \right) _{{\mathcal {D}}ominan\left( {\mathcal {T}}_{k}^{*},C\right) ^{-}}^{\beta \wedge l\left( p\right) }\right| }{\left| \sum \limits _{k=1}^{\left| D\right| }\left( \overline{S^{\Diamond }} \right) _{{\mathcal {D}}ominan\left( {\mathcal {T}}_{k}^{*},C\right) ^{-}}^{\beta \wedge l\left( p\right) }\right| }, \end{aligned}$$

where $S^{\Diamond }\ne \emptyset ,$ and $\left| \cdot \right|$ is the cardinality of a set.

Let $\sum \nolimits _{k=1}^{\left| D\right| }\left( S^{\Diamond }\right) _{{\mathcal {D}}ominan\left( {\mathcal {T}}_{k}^{*},C\right) ^{+}}^{\mu \left( \beta \wedge l\right) \left( p\right) }=1-\sum \nolimits _{k=1}^{\left| D\right| }\left( S^{\Diamond }\right) _{{\mathcal {D}}ominan\left( {\mathcal {T}}_{k}^{*},C\right) ^{+}}^{\rho \left( \beta \wedge l\right) \left( p\right) }$ and $\sum \nolimits _{k=1}^{\left| D\right| }\left( S^{\Diamond }\right) _{ {\mathcal {D}}ominan\left( {\mathcal {T}}_{k}^{*},C\right) ^{-}}^{\mu \left( \beta \wedge l\right) \left( p\right) }=1-\sum \nolimits _{k=1}^{\left| D\right| }\left( S^{\Diamond }\right) _{{\mathcal {D}}ominan\left( {\mathcal {T}}_{k}^{*},C\right) ^{-}}^{\rho \left( \beta \wedge l\right) \left( p\right) }.$ Then $\sum \nolimits _{k=1}^{\left| D\right| }\left( S^{\Diamond }\right) _{ {\mathcal {D}}ominan\left( {\mathcal {T}}_{k}^{*},C\right) ^{+}}^{\mu \left( \beta \wedge l\right) \left( p\right) }$ and $\sum \nolimits _{k=1}^{ \left| D\right| }\left( S^{\Diamond }\right) _{{\mathcal {D}}ominan\left( {\mathcal {T}}_{k}^{*},C\right) ^{-}}^{\mu \left( \beta \wedge l\right) \left( p\right) }$ are called the rough degrees of $S^{\Diamond }$ about ${\mathcal {D}}ominan\left( {\mathcal {T}}_{k}^{*},C\right) ^{+}$ and ${\mathcal {D}}ominan\left( {\mathcal {T}}_{k}^{*},C\right) ^{-}$ respectively.

By definition $0\le \sum \nolimits _{k=1}^{\left| D\right| }\left( S^{\Diamond }\right) _{{\mathcal {D}}ominan\left( {\mathcal {T}} _{k}^{*},C\right) ^{+}}^{\mu \left( \beta \wedge l\right) \left( p\right) }\le 1$ and $0\le \sum \nolimits _{k=1}^{\left| D\right| }\left( S^{\Diamond }\right) _{{\mathcal {D}}ominan\left( {\mathcal {T}} _{k}^{*},C\right) ^{-}}^{\mu \left( \beta \wedge l\right) \left( p\right) }\le 1.$

#### Definition 16

Let ${\mathcal {I}}{\mathcal {S}}^{*}=\left( A,C,D,E\right)$ be an incomplete information system and $S^{\Diamond }\subseteq A,$ $\beta \in \left( 0.5,1 \right]$ and $l\in {\mathbb {N}} \cup \left\{ 0\right\} .$ Then the *approximate quality* $\sum \nolimits _{k=1}^{\left| D\right| }\left( S^{\Diamond }\right) _{ {\mathcal {D}}ominan\left( {\mathcal {T}}_{k}^{*},C\right) ^{+}}^{\gamma \left( \beta \wedge l\right) \left( p\right) }$ and $\sum \nolimits _{k=1}^{\left| D\right| }\left( S^{\Diamond }\right) _{ {\mathcal {D}}ominan\left( {\mathcal {T}}_{k}^{*},C\right) ^{-}}^{\gamma \left( \beta \wedge l\right) \left( p\right) }$ of $S^{\Diamond }$ about ${\mathcal {D}}ominan\left( {\mathcal {T}}_{k}^{*},C\right) ^{+}$ and ${\mathcal {D}}ominan\left( {\mathcal {T}}_{k}^{*},C\right) ^{-}$ are defined by

$$\begin{aligned} \sum \limits _{k=1}^{\left| D\right| }\left( S^{\Diamond }\right) _{ {\mathcal {D}}ominan\left( {\mathcal {T}}_{k}^{*},C\right) ^{+}}^{\gamma \left( \beta \wedge l\right) \left( p\right) }=\frac{ \left| \sum \limits _{k=1}^{\left| D\right| }\left( \underline{ S^{\Diamond }}\right) _{{\mathcal {D}}ominan\left( {\mathcal {T}} _{k}^{*},C\right) ^{+}}^{\beta \wedge l\left( p\right) }\right| }{ \left| A\right| } \end{aligned}$$

and

$$\begin{aligned} \sum \limits _{k=1}^{\left| D\right| }\left( S^{\Diamond }\right) _{ {\mathcal {D}}ominan\left( {\mathcal {T}}_{k}^{*},C\right) ^{-}}^{\gamma \left( \beta \wedge l\right) \left( p\right) }=\frac{ \left| \sum \limits _{k=1}^{\left| D\right| }\left( \underline{ S^{\Diamond }}\right) _{{\mathcal {D}}ominan\left( {\mathcal {T}} _{k}^{*},C\right) ^{-}}^{\beta \wedge l\left( p\right) }\right| }{ \left| A\right| }. \end{aligned}$$

Clearly $$0\le \sum \nolimits _{k=1}^{\left| D\right| }\left( S^{\Diamond }\right) _{{\mathcal {D}}ominan\left( {\mathcal {T}} _{k}^{*},C\right) ^{+}}^{\gamma \left( \beta \wedge l\right) \left( p\right) }\le 1$$ and $$0\le \sum \nolimits _{k=1}^{\left| D\right| }\left( S^{\Diamond }\right) _{{\mathcal {D}}ominan\left( {\mathcal {T}} _{k}^{*},C\right) ^{-}}^{\gamma \left( \beta \wedge l\right) \left( p\right) }\le 1.$$

We now obtain three way decision rules using logical conjunction soft dominating pessimistic multigranulation rough sets:

$\left( \mathrm{i}\right)$ If there exists $k\in \left\{ 1,2,3,...,\left| D\right| \right\}$ such that $\left( \frac{ \left| \left[ b\right] _{{\mathcal {D}}ominan\left( {\mathcal {T}} _{k}^{*},C\right) }^{+}\cap S^{\Diamond }\right| }{\left| \left[ b\right] _{{\mathcal {D}}ominan\left( {\mathcal {T}}_{k}^{*},C\right) }^{+}\right| }\right) >\beta \wedge \left( \left| \left[ b\right] _{ {\mathcal {D}}ominan\left( {\mathcal {T}}_{k}^{*},C\right) }^{+}\right| -\left| \left[ b\right] _{{\mathcal {D}}ominan\left( {\mathcal {T}}_{k}^{*},C\right) }^{+}\cap S^{\Diamond }\right| \right) <l,$ decide $Pos\left( S^{\Diamond }\right) _{{\mathcal {D}}ominan \left( {\mathcal {T}}_{k}^{*},C\right) ^{+}}^{\beta \wedge l\left( p\right) };$

$\left( \mathrm{ii}\right)$ If there exists for all $k\in \left\{ 1,2,3,...,\left| D\right| \right\}$ such that $\left( \frac{ \left| \left[ b\right] _{{\mathcal {D}}ominan\left( {\mathcal {T}} _{k}^{*},C\right) }^{+}\cap S^{\Diamond }\right| }{\left| \left[ b\right] _{{\mathcal {D}}ominan\left( {\mathcal {T}}_{k}^{*},C\right) }^{+}\right| }\right)<1-\beta \wedge \left( \left| \left[ b\right] _{{\mathcal {D}}ominan\left( {\mathcal {T}}_{k}^{*},C\right) }^{+}\right| -\left| \left[ b\right] _{{\mathcal {D}}ominan\left( {\mathcal {T}}_{k}^{*},C\right) }^{+}\cap S^{\Diamond }\right| \right) <l,$ decide $Neg\left( S^{\Diamond }\right) _{{\mathcal {D}}ominan \left( {\mathcal {T}}_{k}^{*},C\right) ^{+}}^{\beta \wedge l\left( p\right) };$

$$\left( \mathrm{iii}\right) $$ Otherwise, decide $$\sum \nolimits _{k=1}^{\left| D\right| }\left( S^{\Diamond }\right) _{{\mathcal {D}}ominan\left( {\mathcal {T}}_{k}^{*},C\right) ^{+}}^{\beta \vee l\left( p\right) }.$$

Similarly, we now obtain three way decision rules using logical conjunction soft dominated pessimistic multigranulation rough sets:

$\left( \mathrm{i}\right)$ If there exists $k\in \left\{ 1,2,3,...,\left| D\right| \right\}$ such that $\left( \frac{ \left| \left[ b\right] _{{\mathcal {D}}ominan\left( {\mathcal {T}} _{k}^{*},C\right) }^{-}\cap S^{\Diamond }\right| }{\left| \left[ b\right] _{{\mathcal {D}}ominan\left( {\mathcal {T}}_{k}^{*},C\right) }^{-}\right| }\right) >\beta \wedge \left( \left| \left[ b\right] _{ {\mathcal {D}}ominan\left( {\mathcal {T}}_{k}^{*},C\right) }^{-}\right| -\left| \left[ b\right] _{{\mathcal {D}}ominan\left( {\mathcal {T}}_{k}^{*},C\right) }^{-}\cap S^{\Diamond }\right| \right) <l,$ decide $Pos\left( S^{\Diamond }\right) _{{\mathcal {D}}ominan\left( {\mathcal {T}}_{k}^{*},C\right) ^{-}}^{\beta \wedge l\left( p\right) };$

$\left( \mathrm{ii}\right)$ If there exists for all $k\in \left\{ 1,2,3,...,\left| D\right| \right\}$ such that $\left( \frac{ \left| \left[ b\right] _{{\mathcal {D}}ominan\left( {\mathcal {T}} _{k}^{*},C\right) }^{-}\cap S^{\Diamond }\right| }{\left| \left[ b\right] _{{\mathcal {D}}ominan\left( {\mathcal {T}}_{k}^{*},C\right) }^{-}\right| }\right)<1-\beta \wedge \left( \left| \left[ b\right] _{{\mathcal {D}}ominan\left( {\mathcal {T}}_{k}^{*},C\right) }^{-}\right| -\left| \left[ b\right] _{{\mathcal {D}}ominan\left( {\mathcal {T}}_{k}^{*},C\right) }^{-}\cap S^{\Diamond }\right| \right) <l,$ decide $Neg\left( S^{\Diamond }\right) _{{\mathcal {D}}ominan\left( {\mathcal {T}}_{k}^{*},C\right) ^{-}}^{\beta \wedge l\left( p\right) };$

$$\left( \mathrm{iii}\right) $$ Otherwise, decide $$\sum \nolimits _{k=1}^{\left| D\right| }\left( S^{\Diamond }\right) _{{\mathcal {D}}ominan\left( {\mathcal {T}}_{k}^{*},C\right) ^{-}}^{\beta \wedge l\left( p\right) }.$$

### Proposed Algorithm-$\mathrm{IV}$ for conflict analysis model

**Input:** Given information system ${\mathcal {I}}{\mathcal {S}}^{*}=\left( A,C,D,E\right) ;$

**Step** 1: Construct $\left( {\mathcal {T}}_{k}^{*},C\right)$ corresponding to the $D_{k}$ for all $k=1,2,...,\left| D\right|$;

**Step** 2: Construct ${\mathcal {D}}ominan\left( {\mathcal {T}} _{k}^{*},C\right) ,$ for all $k=1,2,...,\left| D\right|$;

**Step** 3: Construct $\sum \nolimits _{k=1}^{\left| D\right| }\left( \underline{S_{k}^{\Diamond }}\right) _{{\mathcal {D}}ominan\left( {\mathcal {T}}_{k}^{*},C\right) ^{+}}^{\beta \wedge l\left( p\right) }$ and $\sum \nolimits _{k=1}^{\left| D\right| }\left( \underline{S_{k}^{\Diamond }}\right) _{{\mathcal {D}}ominan\left( {\mathcal {T}}_{k}^{*},C\right) ^{-}}^{\beta \wedge l\left( p\right) }$, where $\beta \in \left( 0.5,1\right]$, $l\in {\mathbb {N}} \cup \left\{ 0\right\}$ and $S^{\Diamond }=\left\{ b\in A:g(b,D_{k})\ne 0\right\} ;$

**Step** 4: If $\sum \nolimits _{k=1}^{\left| D\right| }\left( \underline{S_{k}^{\Diamond }}\right) _{{\mathcal {D}}ominan\left( {\mathcal {T}}_{k}^{*},C\right) ^{+}}^{\beta \wedge l\left( p\right) }=\sum \nolimits _{k=1}^{\left| D\right| }\left( \underline{ S_{k}^{\Diamond }}\right) _{{\mathcal {D}}ominan\left( {\mathcal {T}} _{k}^{*},C\right) ^{-}}^{\beta \wedge l\left( p\right) }$ then $\delta _{k}=\sum \nolimits _{k=1}^{\left| D\right| }\left( \underline{ S_{k}^{\Diamond }}\right) _{{\mathcal {D}}ominan\left( {\mathcal {T}} _{k}^{*},C\right) ^{+}}^{\beta \wedge l\left( p\right) }$ or $\delta _{k}=\sum \nolimits _{k=1}^{\left| D\right| }\left( \underline{ S_{k}^{\Diamond }}\right) _{{\mathcal {D}}ominan\left( {\mathcal {T}} _{k}^{*},C\right) ^{-}}^{\beta \wedge l\left( p\right) }$ otherwise $\delta _{k}=\sum \nolimits _{k=1}^{\left| D\right| }\left( \underline{ S_{k}^{\Diamond }}\right) _{{\mathcal {D}}ominan\left( {\mathcal {T}} _{k}^{*},C\right) ^{+}}^{\beta \wedge l\left( p\right) }-\sum \nolimits _{k=1}^{\left| D\right| }\left( \underline{ S_{k}^{\Diamond }}\right) _{{\mathcal {D}}ominan\left( {\mathcal {T}} _{k}^{*},C\right) ^{-}}^{\beta \wedge l\left( p\right) };$

**Step** 5: $$\delta =\bigcap \nolimits _{k=1}^{\left| D\right| }\delta _{k};$$

**Step** 6: If $$\delta \ne \emptyset ;$$

         go to **output.**

         Otherwise,

         put $\beta ^{\prime }$ in **Step** 3, where $\beta ^{\prime }\le \beta ,$

or

         put $l^{\prime }$ in **Step** 3, where $l\le l^{\prime };$

**Output:** Feasible alternative(s) for feasible consensus strategy.

#### Example 5

(Continued from Example 1) Let ${\mathcal {I}}{\mathcal {S}}^{*}=\left( A,C,D,E\right)$ be an incomplete information system Applying the Algorithm-$\mathrm{IV}$, we have

rom Table 12 and Table 13 one get $\delta =\left\{ b_{7}\right\} .$ The obtained result shows that $b_{7}$ is the optimal decision medicine with decision precision 0.9.

**Table 12:** The lower approximations with $\beta =0.90, l=1$.

$S^{\Diamond }$	$\sum \limits _{k=1}^{\left| D\right| } \left( \underline{S_{k}^{\Diamond }}\right) _{{\mathcal {D}}ominan \left( {\mathcal {T}}_{k}^{*},C\right) ^{+}}^{\beta \wedge l\left( p\right) }$	$\sum \limits _{k=1}^{\left| D\right| } \left( \underline{S_{k}^{\Diamond }}\right) _{{\mathcal {D}}ominan \left( {\mathcal {T}}_{k}^{*},C\right) ^{-}}^{\beta \wedge l\left( p\right) }$
$S_{1}^{\Diamond }$	$\left\{ b_{2},b_{6},b_{7},b_{9},b_{12}\right\}$	$\left\{ b_{1},b_{4}\right\}$
$S_{2}^{\Diamond }$	$\left\{ b_{7},b_{8},b_{9},b_{10},b_{11},b_{12}\right\}$	$\left\{ b_{1},b_{2},b_{4},b_{5},b_{8},b_{9},b_{10},b_{11},b_{12}\right\}$
$S_{3}^{\Diamond }$	$\left\{ b_{6},b_{7},b_{9}\right\}$	$\left\{ b_{4}\right\}$
$S_{4}^{\Diamond }$	$\left\{ b_{7},b_{8},b_{9},b_{12}\right\}$	$\left\{ b_{1},b_{4},b_{5},b_{10}\right\}$

**Table 13:** Decision table with $\beta =0.90, l=0$.

$\delta _{k}$	$\sum \limits _{k=1}^{\left| D\right| }\left( \underline{S_{k}^{\Diamond }}\right) _{{\mathcal {D}} ominan\left( {\mathcal {T}}_{k}^{*},C\right) ^{+}}^{\beta \wedge l\left( p\right) }-\sum \limits _{k=1}^{\left| D\right| }\left( \underline{S_{k}^{\Diamond }}\right) _{{\mathcal {D}}ominan\left( {\mathcal {T}}_{k}^{*},C\right) ^{-}}^{\beta \wedge l\left( p\right) }$
$\delta _{1}$	$\left\{ b_{2},b_{6},b_{7},b_{9},b_{12}\right\}$
$\delta _{2}$	$\left\{ b_{7}\right\}$
$\delta _{3}$	$\left\{ b_{6},b_{7},b_{9}\right\}$
$\delta _{4}$	$\left\{ b_{7},b_{8},b_{9},b_{12}\right\}$

## Novelty and advantages

(i) The proposed model focusses on combining variable precision rough set model with graded rough set model by using logical operators in multigranulation approximate space. Further the proposed multigranulation rough set model which considers the relative and absolute quantitative information about the degree of overlapping between soft dominance classes and a concept set.
(ii) The proposed model answer questions posed by Deja.
(iii) Another advantage of the proposed model is that, it can be used for ranking the feasible alternatives/medicine.
(iv) The proposed model can be used for reduction of attributes.
(v) Using the proposed model, one can find two types of rough degree, precision and approximate quality and their relationship.
(vii) The significance of the proposed algorithms is that, these describe the validity and preferability of the proposed technique as seen in Examples 1, 2, 3, 4 and 5.
(viii) As a real world application, the proposed model can be applied to solve the problem of Middle East conflict analysis problem, determining the governor election results in Indonesia, selection process at university level, for selection of appropriate medicine for a disease.

## Conclusion

In this article we firstly initiated the notion of soft preference and soft dominance relation for the development of soft dominance rough set in an incomplete information system. Subsequently, several important structural properties and results of the proposed model are investigated. We observed that after employing soft dominance based rough set approach to it for any times, we can only get six different sets at most in an incomplete information system. That is, every rough set in a universe can be approximated by only six sets, where the lower and upper approximations of each set in the six sets are still lying among these six sets. The relationships among these six sets are established. Based on soft dominance relation, the logical disjunction/conjunction soft dominance optimistic/pessimistic multigranulation decision theoretic rough approximations in an incomplete information are established. Thereafter, a novel multi attribute with multi decision making approach based on logical disjunction/conjunction soft dominance optimistic/pessimistic multigranulation decision theoretic rough sets approaches are developed to select the medicine to treat the corona virus disease (COVID-19). The basic principle and the steps of the decision making model (algorithms) are presented in detail. To demonstrated the applicability and potentiality of the developed model, we presented a practical example of a medical diagnosis.

In future, the proposed model can be extended to some other fields such as risk evaluation, hotel location, project selection, Middle East conflict analysis problem, determining the governor election results in Indonesia, selection process at university level, for selection of appropriate medicine for a disease and other domains under conflict environments.
